# Robot leadership–Investigating human perceptions and reactions towards social robots showing leadership behaviors

**DOI:** 10.1371/journal.pone.0281786

**Published:** 2023-02-16

**Authors:** Jakub Edward Cichor, Sylvia Hubner-Benz, Tobias Benz, Franziska Emmerling, Claudia Peus

**Affiliations:** 1 Neurophysiological Leadership Lab, Technical University of Munich, TUM School of Management, Munich, Germany; 2 Faculty of Economics and Management, Free University of Bozen-Bolzano, Bozen-Bolzano, Italy; 3 Merkle DACH, Munich, Germany; Polytechnic Institute of Coimbra: Instituto Politecnico de Coimbra, PORTUGAL

## Abstract

Human-robot interaction research has shown that social robots can interact with humans in complex social situations and display leadership-related behaviors. Therefore, social robots could be able to take on leadership roles. The aim of our study was to investigate human followers’ perceptions and reactions towards robot leadership behavior, and differences based on the robot’s displayed leadership style. We implemented a robot to show either a transformational or a transactional leadership style in its speech and its movements. We presented the robot to university and executive MBA students (N = 29) and subsequently conducted semi-structured interviews and group discussions. The results of explorative coding indicated that participants differed in their perceptions and reactions based on the robot’s leadership style and based on their assumptions about robots in general. We observed that participants quickly imagined either a utopia or worried about a dystopia, depending on the robot’s leadership style and their assumptions, and that a subsequent reflection led to more nuanced views. We discuss the implications and recommendations for human-robot interaction and leadership research.

## Introduction

As digitalization proceeds, novel technologies represent both challenges and opportunities for organization [1]. Robots represent one promising technology; their utilization has doubled across domains and organizations between 2013 and 2018. Robots have demonstrated the ability to take over menial tasks from humans and to help in dangerous situations such as rescue missions. Due to their constantly improving social capabilities, robots are now also introduced into complex social situations [2]. One relevant complex social interaction for organizations is leadership; preliminary results have hinted towards the potential of social robots to partake in leadership interactions, for instance by motivating people to continue to work on a mundane task [3].

Although human-robot interaction (HRI) research provides first insights regarding boundary conditions of robot leadership, knowledge on how robot leaders should appear and behave in order to be accepted as leaders, and to be effective leaders, is lacking. Previous studies on robot leadership only considered a small subset of the behaviors that would be required from human leaders. To understand which behaviors are effective in robot leaders, knowledge on human perceptions and reactions towards specific robot behaviors is needed [4–6].

With our explorative, qualitative study, we investigate how human followers perceive and react to specific leadership behaviors when portrayed by a robot, i.e., when a robot takes on the leadership role. Our research integrates the robot-based findings from HRI research [2, 7], which investigated capabilities and perceptions of social robots, and the human-based findings from leadership and organizational behavior research [8, 9], which examined follower reactions to leadership behaviors in humans. We answer the question: ‘What are the perceptions and reactions a robot leader elicits in human followers, and how do these perceptions differ based on the robot’s leadership style?’

Our research contributes to the HRI and leadership literature. For HRI literature, our research adds an examination of human perceptions and reactions towards robots in a mainly unexplored complex social situation, specifically robot leadership. For the leadership literature, we provide an examination of the perceptions and reactions towards leadership behaviors in robots, which is a new perspective because prior leadership literature only considered human leadership.

We begin this paper with a theoretical background to demonstrate the current stage of research on robot leaders. We then elaborate on our method, and explain how we conducted and analyzed our interviews and group discussions. Then, we describe our results including the assumptions, reactions, ethical concerns, and future visions that we observed. Finally, we discuss our results, the study’s limitations, suggestions for future research, and then finish the paper with a conclusion.

### Theoretical background

HRI research showed that robots can be utilized successfully for specific leadership-related behaviors. Initial research on robots engaging in motivational behavior indicates that robots can use algorithms to identify the most motivated employees [10] and motivate participants to work on mundane tasks, where otherwise they would be likely to quit [3]. Thus, robots seem to be able to identify and increase motivation levels, which is an important component of leadership [11]. When humans work with a robot for a prolonged period of time, they have been found to even prefer robot leadership to human leadership when they perceive it to increase efficiency [4]. Thus, humans seem to accept social influence from robots [12–14]–an important prerequisite for robots to be taken seriously in leadership roles.

For robots to effectively take on leadership roles, robot acceptance and trust in the robot are crucial. Robot acceptance has been shown to be dependent on robot design and behavior. The specific aspects that are effective depend on task context. In casual social settings, for instance, playful humanlike robots are preferred over mechanical, serious ones [15]. In leadership roles, robots need to behave in a way that makes them appear trustworthy. Trust in robots is important for human-robot interaction [5], and trust in leaders is an important component in an effective follower-leader-relationship [9]. Therefore, trust in the robot leader is important for a collaborative work relationship between human followers and robot leaders [16]. Initial research on trust in robots suggests that the process through which trust between human and robot develops is very similar to how humans begin to trust one another [5]. Crucial in this process is mind attribution, defined as the potential of the robot to be seen as being capable of experience and agency. Mind attribution increases likability [17] as well as trust [18]. While trust in technologies can decrease after encountering technical errors [19], direct, prolonged interactions with technologies can increase acceptance and trust, and in turn mind attribution [20].

What humans expect from robots and how they interact with robots is also influenced by their assumptions [21]. More specifically, humans’ assumptions pertaining to robots [22], e.g., whether they see the robot as a tool or a social actor, influence the way human-robot interactions unfolds [23]. The importance of assumptions has been shown in HRI research; having a generally positive attitude while simultaneously attributing little agency to a robot was found to make interaction more likely [24]. In the context of robot leaders, interactions might be influenced by humans’ assumptions of what robot leadership behavior looks like. A robot leader might be envisioned as an emotionless machine and lacking personal connection; or ‘objective’ fair treatment and competent decision making might be attributed to a robot leader. Crucially, negative assumptions towards robots are likely to decrease after successful interactions with them [22]. Moreover, when human followers assume the robot leader is programmed and optimized to support them, they could perceive increased organizational support, which has been linked to well-being [25, 26]. The concrete content of such assumptions is relevant as it determines whether and, if so, how human followers engage in and react to interactions with a robot leader.

We suggest that human perceptions and reactions towards robot leadership are likely to depend on the robot’s leadership ‘style’. For human leaders, research has identified certain leadership styles as particularly effective. For example, transformational leadership, the most extensively studied leadership style, combines idealized influence, inspirational motivation, intellectual stimulation, and individualized consideration [27]. Transformational leadership has been linked to a multitude of positive effects on, for instance, organizational commitment, creativity, engagement, and trust [9, 28–31] in a variety of contexts [9, 32]. Transactional leadership, in contrast, is characterized by management-by-exception (active, passive) and contingent reward and is suggested to be particularly effective under time pressure and for tasks that do not require creativity [27]. To this date, only one study has been published that investigated transformational and transactional robot leadership. The researchers found that the transformational robot leader lead to higher engagement in trust, whereas the transactional robot leader increased performance in a manual tower building task [33]. However, the effects of a semi-humanoid social robot leader in a realistic leadership task are yet to be studied.

Our study explored the potential of implementing leadership behaviors in semi-humanoid social robots and the hopes and fears that followers associated with robot leadership. We programmed a social robot to display leadership behaviors. We investigated differential reactions to transformational and transactional robot leadership; we expected that a transformational–compared to a transactional–robot might have more positive effects on human followers, based on the positive effects transformational leadership has in human leaders [9, 28, 33].

## Method

### Research approach

As only little research has been conducted on leadership behaviors in robots so far, we chose a qualitative approach for our study. Participants either saw a transformational or a transactional version of the robot. In each version, they received a task from the robot, and subsequently worked on the task ‘for the robot.’ After that, we observed their perceptions and reactions. In the interview study, we informed participants orally that the participation is voluntary, that they can withdraw at any time, and that their answers will be recorded and evaluated to be used for scientific research purposes. For participants in the group discussions, written informed consent was obtained at the beginning of our study, where they were also informed that their participation is voluntary, that they can withdraw at any point, and that research notes taken during the group discussions will be used in research. As no ethics committee for behavioral experimental sciences existed at the institution at which this research was conducted, we carefully implemented ethical principles for research involving human participants as provided by the Declaration of Helsinki and the German Society for Psychology (DGPS).

**Fig 1:**
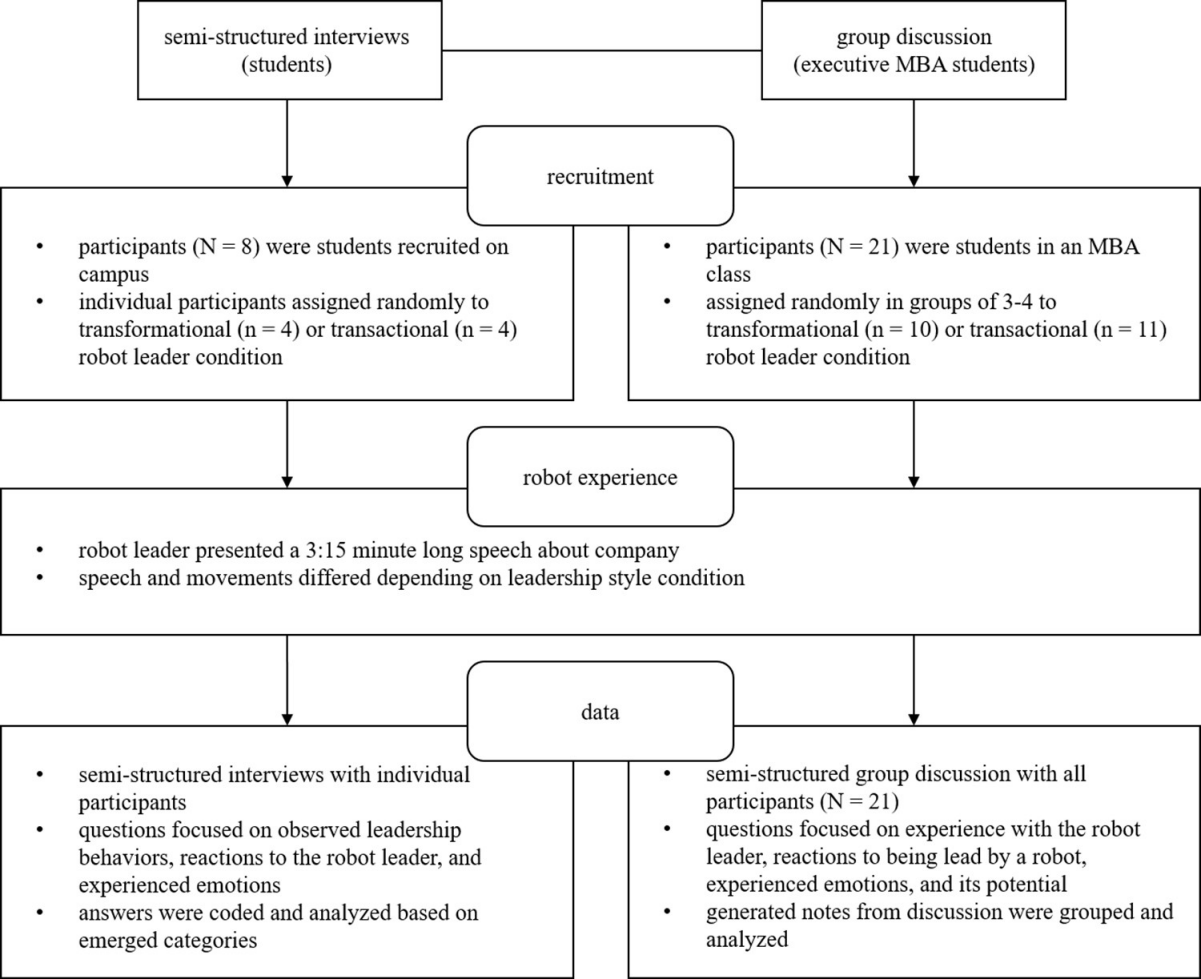
Overview of the procedures for both studies.

We integrated different data sources to get a differentiated picture of participants’ perceptions and reactions in two stages (see Fig 1). In the first stage, we conducted semi-structured interviews to obtain detailed insights into how our interviewees experienced the interaction with the robot leader. The interviews were conducted by a male Master’s student, who was supervised by the second author, was given detailed instruction before the study on how to conduct semi-structured interviews, and, subsequently, prepared the questions together with the authors. Only the student and the interviewee were present during the interviews and the interviews were recorded with an audio recorder. No relationship was established with participants prior to the interviews, as the participants were approached on campus to take part in the study. Participants were informed that they would experience an interaction with a robot and that an interview would be conducted afterwards, which would be a part of the student’s Master’s thesis. Each interview lasted around 45 minutes.

In the second stage, the first author oversaw semi-structured group discussions to observe controversial conversations between participants, which triggered in-depth reflections on the experience with the robot leader. The researcher had no prior interactions with the participants of the group discussions, but the participants received the researcher’s CV before the group discussions and were told that the session would be on the topic of robot leadership. Additionally, the participants knew that the study was being conducted as a part of the first author’s Ph.D. research. A research assistant and a lecturer were present during the discussions to take research notes and to keep track of time, resulting in discussions that lasted around 20 minutes.

In the interviews and group discussions, following a phenomenological approach, our aim was to understand what participants experience during a live interaction with a robot leader [34]. We asked questions and gave prompts related to working with a robot leader, conditions under which a robot leader would be promising, and in which aspects it was convincing in its leader role. We asked for participants’ perception of the robot, whether they thought the robot could be a leader, how they felt while working for the robot, and what they thought working for a robot in a real-life setting would look like. We also compared the insights from our interviews with the insights from our group discussion to investigate similarities and contradictions.

### Implementing leadership behaviors in a robot

We programmed the social semi-humanoid robot Pepper by SoftBank Robotics to act as either a transformational or a transactional leader during a presentation delivery, whereas each version took around 3 minutes and 15 seconds. The difference between leadership styles was emphasized by adjusting the robot’s spoken text and its movements. The transformational [27] version of the robot referred to its vision for its company, its confidence in its followers to complete the stated task, and in its enthusiasm for its work, while displaying large projecting movements away from its body (e.g. throwing its hands up in the air when displaying enthusiasm), which were based on the charismatic leadership tactics [35]. In comparison, the transactional [27] robot leader focused on rewards for completing the task, setting specific goals, and specifying concrete requirements, while showing movements closer to its body and by displaying more directive movements like shaking its head–representing counter-acting movements to charismatic leadership tactics [35]. Transformational and transactional robots were tested with leadership experts prior to the first session.

In both versions, the robot stated that it represented the head of a marketing department of a botanical company specializing in products that are supposed to help people with growing plants. After introducing the company and its product, the robot instructed participants to create a marketing strategy for the product, based on a set of guiding questions. The task of developing the marketing strategy aimed to create a realistic situation where participants feel like they are working ‘for the robot’. The study focused on the situation of a leader handing a task to subordinates (i.e., we here did not investigate the situation of subordinates presenting their work results to the robot leader).

### Data collection

#### Interviews

For our interviews, 8 participants were recruited on campus via the authors’ networks; participants were employees (academic or educational staff) or students at the university. The participants were presented with the robot’s presentation (4 participants with the transformational and 4 with the transactional version of the robot) and then completed the task ‘for the robot’. Subsequently, we conducted the in-depth interviews. In a semi-structured approach, we started with a set of relevant questions (sample questions in Table 1) and then diverged into additional topics whenever interviewees made an interesting statement. We aimed to establish a natural conversation. No participants dropped out of the study. We did not conduct repeat interviews and we did not give the recordings or transcripts to participants. Data collection was stopped when we started to find repeating themes in the participants’ responses.

**Table 1:** Questions stated during interviews and group discussions.

Data collection method	Question
Interviews	Transactional specific: How do you think that Pepper would react if you were to make a mistake?
Interviews	Transactional specific: What did you think when Pepper said “We do not tolerate any mistakes”?
Interviews	Transactional specific: Pepper said: “You should document what you do so that I am able to follow your steps and identify mistakes. This will allow me to give you exact feedback on your work.” Did you follow his steps exactly? Why?
Interviews	Transformational specific: What did you think when Pepper said: „My vision is that we, as the marketing department, can make people passionate about our product.“?
Interviews	Transformational specific: How well did Pepper define and present his vision for the future?
Interviews	Transformational specific: Pepper said: “As a leader, I always want to be a role model.”What do you think about that? To what degree do you think that Pepper could be a role model for you as an employee?
Interviews	Transformational specific: If you were now in a team with Pepper as a leader: Would you aim to reach the goals that Pepper sets out for you?
Interviews	Transformational specific: To what degree do you feel that Pepper would have high expectations for you?
Interviews	Transformational specific: To what degree do you believe that Pepper would support you appropriately as a team member? Why?
Interviews	Transformational specific: To what degree do you have the feeling that Pepper adjusts himself to you and others, for example, by paying attention to your personal needs?
Interviews	To what degree did Pepper inspire you to produce new and innovative ideas?
Interviews	Do you think that Pepper would question the status quo? To what degree?
Interviews	Would you do more than what is minimally expected to reach the goal of Pepper’s department?
Data collection method	Question
Interviews and group discussion	Which thoughts went through your head when Pepper was talking to you?
Interviews and group discussion	How did it feel when you were addressed by Pepper?
Interviews and group discussion	What was your overall impression of Pepper?
Interviews and group discussion	To what degree did you feel motivated by Pepper? Imagine that you are working in Pepper’s department: To what degree would you feel motivated by Pepper?
Interviews and group discussion	Do you feel appreciated by Pepper? Why?
Interviews and group discussion	Do you think that Pepper is fair to his team members? Why?
Interviews and group discussion	Can you trust Pepper as a leader? Why?
Interviews and group discussion	Which of his behaviors did you like? Why?
Interviews and group discussion	What did you not like about his behavior?
Interviews and group discussion	How would you feel when Pepper was your leader? What would work particularly well or particularly badly?
Interviews and group discussion	If Pepper gave you strict feedback: How would you take it?
Interviews and group discussion	How well do you think can Pepper create plans and set goals for the future of his team?
Interviews and group discussion	What would you expect: How successful would a team led by Pepper be? Why?

#### Group discussions

For the group discussions, 21 company leaders or prospective leaders participating in an executive education program were recruited. Due to their comprehensive work experience, their perspectives on robot leadership were particularly interesting as they could compare to their experiences with human leaders and their own leadership approach. The participants were presented with the robots’ presentations in groups of 3–4 (10 transformational and 11 transactional leadership), and then worked ‘for the robot’ individually. Subsequently, we conducted a focus group discussion with all 21 participants. The discussions were based on a set of pre-defined questions that overlapped with our interview questions. Throughout the discussion, multiple participants were encouraged to answer each question, and natural discussions between participants were welcome. All participants completed the entire study procedure, no repeat discussions were carried out, and participants did not comment on the research notes. The group discussion emphasized the exploration of themes related to robot leadership and facilitated an exchange between participants. Research assistants took detailed research notes pertaining to the points and themes participants made during the exchange. After we found that the group discussions did not introduce new themes when compared to the interviews, we stopped the data collection.

### Data analysis

**Fig 2:**
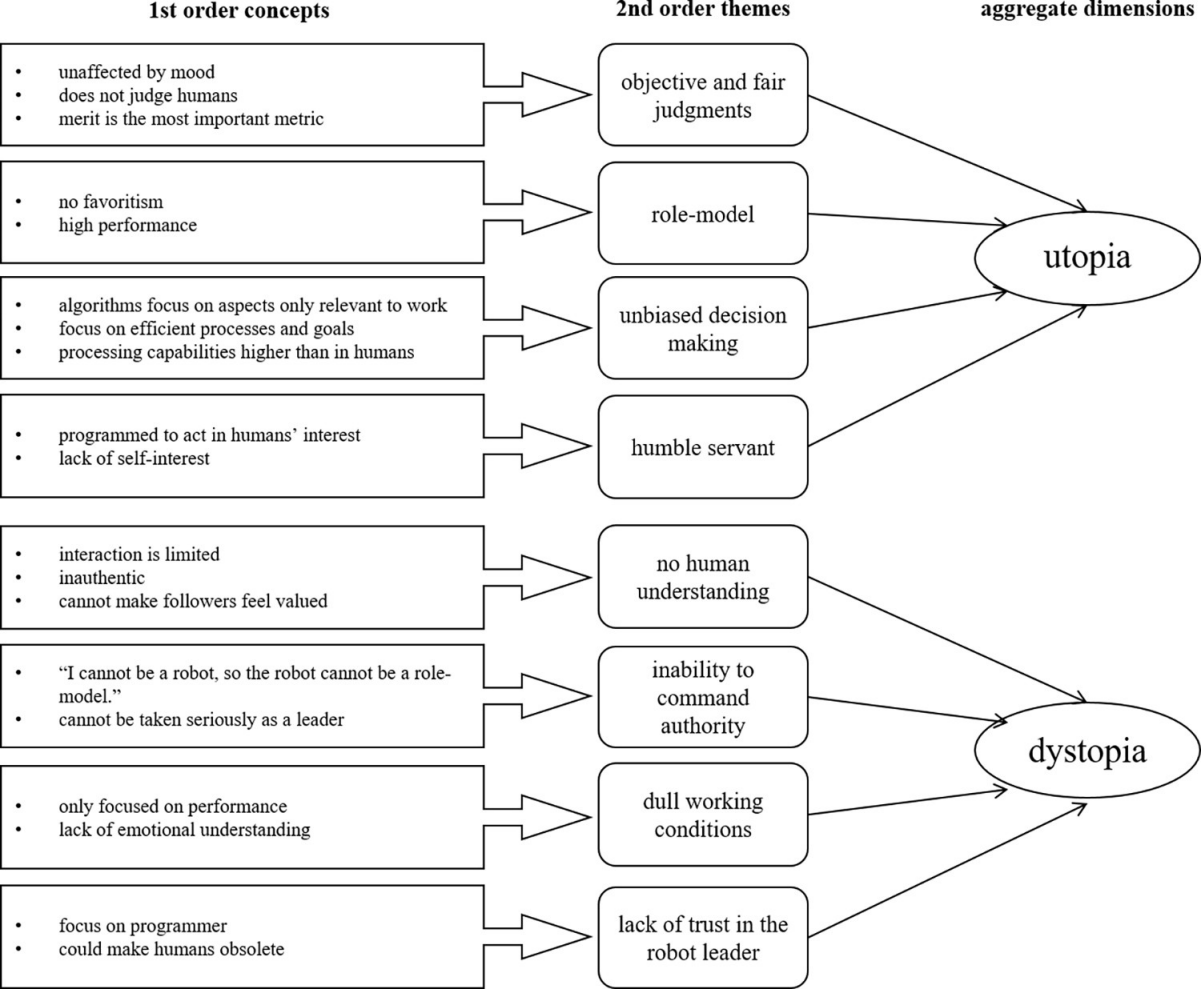
Gioia methodology based on the interviews and group discussions.

The first and second author coded the transcripts of the interviews based on an iterative process in the MAXQDA software by VERBI GmbH. First, we coded statements regarding robot perception. For example, we coded when participants stated that they found the robot inspiring or intimidating. In this step, we also coded the reasoning for whether participants thought the robot could be a leader. Second, we coded statements on how participants felt during working for the robot, e.g., whether participants found themselves engaged or bored with the task. Third, we coded what participants thought working for a robot in a real-life setting would look like. We explicated whether participants pictured similar situations and which concrete positive and negative aspects they mentioned. Fourth, we created codes for all emotions, fears, and envisioned scenarios that were mentioned by participants. After the coding procedure, we structured our findings according to four categories that emerged: i) general assumptions regarding robot leadership, ii) reactions to specific robot leadership behavior, iii) emotional reactions to the robot leader, and iv) future visions and ethical concerns. By having two raters iteratively code the interview statements and repeatedly comparing the agreement between raters, we were able to ensure interrater reliability in our coding process. How the participants’ ideas fit the individual categories is visualized in Fig 2. Most perceptions and reactions appeared in the interviews as well as the group discussions. The similarities indicated high ecological validity of our approach, especially as both data collection methods followed natural conversations and allowed for diverse perspectives. The similarities between findings from interviews and group discussions also emphasized high test-retest reliability. Interviews were originally conducted and coded in German. The authors subsequently translated selected statements into English to exemplify the participants’ key ideas in the paper.

## Results

**Fig 3:**
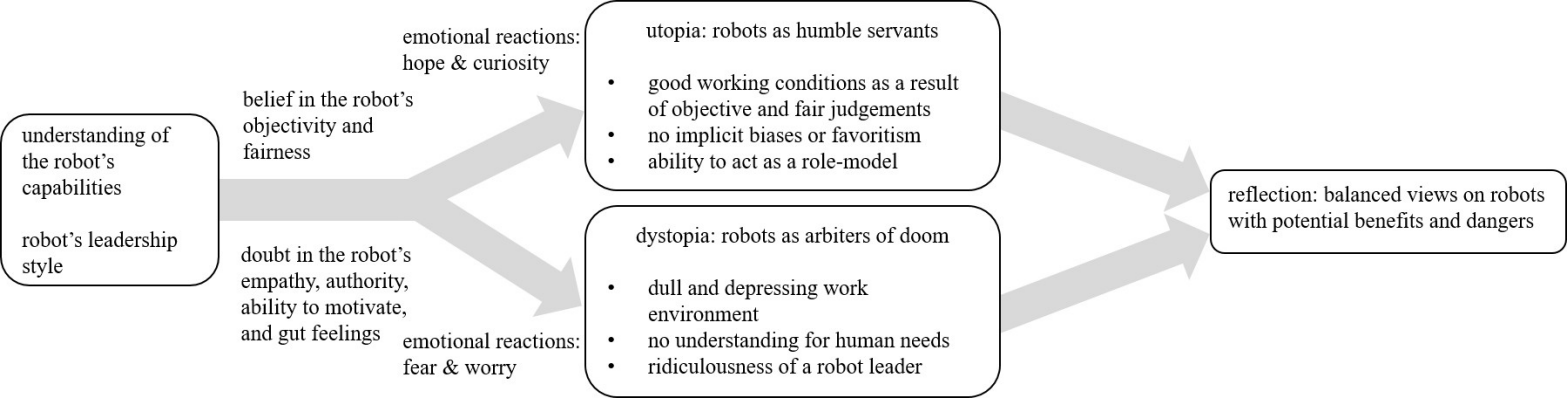
Synthesized overview of the participants’ ideas.

Data analysis showed that a combination of the encountered leadership style and the assumptions participants held with regard to robots influenced their subsequent perceptions. Depending on whether the participants had positive or negative assumptions, their emotional reactions were primarily characterized by either hope and curiosity or fear and worries. The emotional reactions subsequently lead to the participants imagining either a utopian fantasy where robot leaders would be humble servants to humans, or a dystopian scenario in which robot leaders would severely hurt our work environment as we know it. Additionally, the leadership style exhibited by the robot leader also affected the participants’ reactions. The transformational robot leader was generally judged as a more suitable leader and, hence, frequently resulted in a more positive outlook by the participants on the robot leader than the transactional robot. In Fig 3, we illustrate these results on how humans’ assumptions about robot leadership and the robots’ leadership styles affect humans’ perceptions, judgments, and reactions, and in turn stimulate utopian or dystopian scenarios.

### General assumptions regarding robot leadership

Participants assumed that a robot leader would predominantly be used to communicate tasks and to evaluate human performance. Participants expected that a robot leader would be capable of stating and monitoring goals based on quantifiable metrics. However, they also thought that robots’ personal interaction would fall short and could not envision ‘true’ interactions with the robot. One interviewee who interacted with the transactional robot for example said: “I believe that I would see Pepper as an instructor and evaluator who judges my performance fairly und relays the instructions to me. But not the interaction between [us].” (Participant #1) Such assumptions were also prevalent in our group discussions where participants saw potential in a robot in the role of an instructor. They saw its primary function in relaying instructions to employees but could not imagine the robot fully encompassing the role of a leader. However, as these assumptions were primarily stated by participants who engaged with the transformational robot, they could also be related to a future vision as a result of the interaction with the robot leader.

Interestingly, we observed that the participants’ judgment of whether a robot could be a suitable leader depended on their technological knowledge on robots in general and their assumptions regarding robots. For instance, when participants thought about the robot’s suitability as a leader (e.g., being empathic, showing authority, motivating employees), they frequently doubted its ability to display relevant leadership abilities–an assumption stated more frequently when interviewees encountered the transactional robot. As a result, they worried that robot leaders would have negative effects on organizations in the future. If, however, the participants had prior technological knowledge on robots and their capabilities, they saw potential in robot leaders. For instance, they expected that robot leadership would reduce favoritism, as one interviewee stated after interacting with the transformational robot: “Yes, I think that based on how he is programmed, he will not be unfair. And because he is not led by emotions but rather shows an objective approach, I believe that he is fair.” (Participant #2)

### Reactions to specific robot leadership behavior

The analysis of reactions to working for the robot shed light on the hopes and fears that humans associate with robots in leadership functions. Participants hoped to be challenged to do their best work, especially by the transformational robot. The transformational robot’s goals were taken seriously, participants imagined the robot could have positive motivational effects on them, and imagined they could use it as a de-facto “role-model” that guides them in their progress. One interviewee who saw the transformational robot stated: “Because [the robot] spoke with a certain emotion and also passion, I would say that he did it well and therefore also motivated me to develop good ideas.” (Participant #3)

The transactional robot, however, was generally seen to create a depressing and dull work environment. Participants found it unlikely that working for a transactional robot leader would enable them to learn and grow, and thought its leadership would be predominantly suited for tasks that do not rely on creativity. We found that–compared to the transactional robot leader–participants took the transformational robot leader more seriously. The transformational robot leader inspired hopes including increased motivation, fair judgment, and decision quality whereas the transactional robot leader stimulated worries and fears regarding considerations of human needs and room for creativity and mistakes.

### Emotional reactions to the robot leader

Irrespective of the encountered robot leadership style, the participants showed both hopes and worries after their encounter with the robot leader. On the one hand, hopes were often related to its lack of emotions, which some participants imagined would lead to unbiased decision making and a lack of favoritism. Additionally, due to the robot’s mechanical nature participants attributed efficiency and the ability for machine learning. On the other hand, we found that many participants doubted the robot’s ability to show and detect the range of emotions to be expected from a suitable leader. For instance, participants frequently identified the robot’s lack of empathy as a major problem and could not imagine how a robot could react empathically to their actions and needs. One interviewee who encountered the transformational robot stated for instance: “As I said, I would not be able to build an emotional connection [with the robot leader], so I would not feel appreciated.” (Participant #4)

Participants generally lacked trust towards the robot as they felt that for building trust, an interaction on an emotional and, therefore, necessarily human level would be required. This perception was more common after interactions with the transactional robot and it was subsequently confirmed in our group discussions, where participants expressed worries regarding the robot’s empathic capabilities and subsequently its fit as a leader. The lack of empathy was on the one hand related to clear communication, but on the other hand a lack of concern for personal development. For example, one interviewee stated after encountering the transactional robot: “And I would most likely be mad, because I currently do not see how [the robot leader] could help me for example in my career development.” (Participant #5) All in all, some participants saw the lack of emotions as a benefit for fairness and efficiency, while others worried about not being understood and having their needs as employees not met.

### Future visions and ethical concerns

We could identify two basic cognitive scenarios emerging from the encounter with robot leadership. We found that utopian fantasies are contrasted with dystopian fears.

If the participants took the positive perspective, they frequently stated how robot leaders would provide advantages pertaining to being more objective and fair leaders due to a lack of biases and emotions. Consequently, these participants’ emotional reactions were primarily characterized by hope and curiosity for the applications of robot leaders, which resulted in an idealization of robot leaders and a utopian vision. Participants believed that robot leaders would essentially be humble servants and therefore highly objective, unbiased, capable of competent decision making, and able to learn from their mistakes by virtue of machine learning. If, however, the participants had a negative initial view on robots, they immediately questioned the potential of robot leaders based on the attribution of a lack of empathy, authority, and motivational capabilities.

We encountered ethical considerations regarding leadership responsibility in robots and worries pertaining to the future. Participants questioned to what degree they were interacting with the robot directly, and not just the robot as a programmer’s vessel. They stated that they would need to know and trust the programmer in order to be able to trust the robot. One interviewee with the transactional robot for example said: “For that I would like to get to know the programmers first. Can I trust him? No.” (Participant #6) This concern was also pushed further in the direction of algorithms, as the participants worried they would only be led by whatever the programmer designed to be in the algorithm and not by an actual leader, which, in turn, would make them question to what degree there is any leadership involved in the first place. For instance, one interviewee stated after interacting with the transactional robot: “I always find [robot leaders] difficult, because it is a robot and someone has programmed it. And then the question is: Can I trust the programmer and not [just the robot leader]?” (Participant #7)

Some participants ascribed abilities and hopes to robot leaders that currently are, to the best of our knowledge, far beyond the actual technical specs of robots. In contrast, beyond doubting the robot on its leadership-related suitability, some participants worried that robots could soon dominate humans in various areas of life. We found that, depending on the participants’ assumptions and the leadership style exhibited by the robot, their subsequent judgment swiftly radicalized. The resulting emotional reactions, then, were permeated by fears and worries, which is why these participants were preoccupied by fears of an impending dystopia, in which robot leaders would have no understanding for human needs, work would change to require no creativity or originality, and robot leaders would not be able to lead effectively due to not being taken seriously by their followers.

Additionally, during the interviews, we noticed that many participants evolved in their evaluations of the robot as their reflection on the interaction progressed. Participants who were skeptical initially and doubted any usefulness of the robot leader often came, in the course of the interview, to a new conclusion. Sometimes even while answering one single question a shift in a participant’s evaluation of the robot leader could be observed. Participants’ assumptions seemed to have a major effect on their following judgments and reactions to robot leaders, causing them to quickly think of the best or worst imaginable scenarios. However, thinking about robot leaders and reflecting on their benefits and dangers made many participants reconsider their initial judgments and develop a more nuanced viewpoint on robot leaders by considering both positive and negative aspects of robot leadership.

## Discussion

In this study, we implemented leadership behaviors in social robots and investigated human perceptions and reactions to those behaviors. Based on our findings, we conclude that assumptions and specific robot leadership styles influence judgments and reactions towards robot leadership and determine future visions. Depending on their initial understanding of robot capabilities and on the robot’s leadership style participants evaluated robot leadership either positively or negatively. Two types of future visions with respect to robot leadership were identified; in utopian scenarios robot leaders are pictured as ‘humble servants’ to humans and in dystopian scenarios robot leaders are foreshadowed as ‘arbiters of doom.’

The ethical challenges surrounding the usage of robot leaders were central to all evaluations. Worries based on dystopian scenarios indicated that uncertainty regarding the programmer’s role in the robot leader’s behavior and fears on robots being unemphatic leaders created unanswered questions in our participants. Specifically, the possibility of humans having to deal with robot leaders that might not recognize human needs sparked resistance to the idea of robot leadership. Crucially, while initial ideas on robot leaders reverted around utopian or dystopian extremes, these ideas were tempered through reflective processes triggered by our interviews and group discussions, during which participants re-evaluated their initial reactions and developed more balanced views on the perks and perils of robot leadership. Participants frequently pointed out how they were hopeful as robots had the potential of being more objective and fairer than human leaders, while still considering that robot leaders might not be the solution to all problems traditionally attributed to human leaders.

### Implications

Our study is a first step towards understanding human judgments and reactions to robot leadership and contributes to the leadership as well as HRI literature in three ways. First, we were able to provide initial insights into the robot- as well as human-based factors influencing judgments and reactions to robot leadership behaviors. Participants could imagine being motivated by a robot leader and were willing to trust it under the right circumstances, which confirms findings from prior research [5, 10]. Our findings emphasize the central role of assumptions in human-robot interaction, which have been pointed out to substantially influence interactions between humans and robots in previous HRI studies [23]. We found, in the specific context of robot leadership, that initial positive or negative ideas on robots in general affected subsequent judgments regarding the potential of robot leadership and initial future visions.

Second, our findings indicate that human perception of a robot leader mirrors the perception of a human leader. Previous leadership literature showed that transformational leadership behaviors are generally preferred over transactional leadership behaviors [36]. In our study, participants frequently stated that the transformational robot leader was able to convey a compelling vision, expected it to support them in their growth by posing appropriate challenges, and saw it as a potential role-model they could learn from. Other participants stated that the transactional robot might be unempathetic and dull. These differences with respect to the leadership behaviors indicate that the transformational robot stimulated more positive reactions than the transactional robot.

Third, we determined that detailed reflections on the role of robot leaders were able to fundamentally affect whether humans adopt a balanced and nuanced view towards robot leaders. This finding indicates that balanced discussions and reflections are needed to develop nuanced viewpoints on robot leaders, especially regarding ethical concerns that are critical to the perception of robot leaders. Reflecting on own views and second-guessing own judgments, stimulates a realization that robot leaders provide certain advantages like a lack of bias and less favoritism, but at the same time lack empathy and are not able to take individual considerations into account making them unlikely to completely replace human leaders.

### Limitations and future research

Our study only included of a small sample and explorative analyses; future research needs to elaborate and replicate our results. Crucially, future research should aim to experimentally test leadership style differences in robot leaders, and differences due to human assumptions, to verify and extend the findings of our explorative, qualitative study. Moreover, further leadership styles–including not only transformational, transactional [27], but also further constructive as well as destructive styles [37] and their effects on followers’ task engagement and performance should be investigated. As our work predominantly focused on the perceptions and reactions to robot leaders, further research should explore how specific perceptions and reactions are formed and by which personal characteristics they are influenced. Additionally, investigating how the robot leader could react if participants hand-in their results for the robot to evaluate would also be a promising avenue for future research.

Accordingly, cultural differences with respect to exposure to robots and prior experience with human leaders should be taken into account by future research. Such research could indicate how robot leadership behaviors can be adapted to ensure fit to employees’ needs which may differ based on preferences, personality types and cultural backgrounds. Further studies should also look into the technological savviness of participants, for instance by measuring traits like affinity for technology interaction [38] and investigating its connection to assumptions and concerns about robots, which were beyond the scope of our study.

## Conclusion

We could observe an interplay between the robot’s leadership style and the human followers’ assumptions about robots in shaping the human followers’ perceptions and reactions towards robot leadership. When interacting with the transformational robot leader or having positive assumptions pertaining to robots, a positive view was adopted and the potential for robot leaders to be fairer and more competent than humans was salient. In contrast, when encountering the transactional robot leader or having negative assumptions or higher uncertainty about the robots’ capabilities, worries about the dangers of robot leaders and their potential negative effect on future society were common. Nevertheless, over the course of a subsequent reflection, when considering the negative and positive sides of robot leaders and thinking about the interaction in more detail, there seems to occur a more balanced view on what the utilization of robot leaders might bring for the future.

Our research enlightens how humans perceive and react to robot leaders and can, thus, open up novel vistas on how organizations can integrate robot leaders effectively and safely into their work environments. Whether ultimately robot leaders will be our humble servants or arbiters of doom, remains to be discovered. Evidence indicates, however, that hopes and fears emerging around robot leadership rely less on the technology in question and much more on us–the human individual interacting with the technology.

## Supporting information

S1 Checklist COREQ checklist to indicate where the information can be found in the manuscript. (PDF)

S1 Dataset Underlying dataset consisting of interviews and research notes. (ZIP)
