# Supplementary material for: Robot leadership–Investigating human perceptions and reactions towards social robots showing leadership behaviors
Source: PLoS One. 2023 Feb 16;18(2):e0281786. doi: 10.1371/journal.pone.0281786 (PMC9934409; doi:10.1371/journal.pone.0281786)
Supplement: S1 Dataset — (ZIP) [file pone.0281786.s002.zip › 190723_Group_Discussions.docx]

**Short Survey (per hand raise): “How successful would pepper act as a leader?”**

🡪 0 for successful

🡪 most of the participants: 1/10 successful

**Initial Feedback:**

- No interaction
- What are the movements there for?
- No chance to interact
- Why did we split the rooms? 🡪answer: for more realistic interaction with leader (in a small group)
- Expectation: interaction with Pepper 🡪 lack in clear expectations and definition what to do
- No connection seen between this lecture and the pepper sessions: don’t see the WHY?

**Scenario: “Imagine: Boss leaves and his successor would be Pepper”**

*Where do you see the interaction in five years? Expectations to robotics?*

- Expectation to a leader/boss:
- Sympathy
- Empathy
- Responsibility
- solving complex problems, which are unseen; rather rule-based problems (which can be programmed)

🡪 Problems mostly novel and not solvable by AI

- Missing empathy: possibility to be able to negotiate and reason with boss
- Do we get the answers we want to have? Which are not pre-defined?

🡪 Feedback and interactions are vital

🡪No clarity, because one cannot ask any questions

- Managers often get emotional

🡪 People need human interaction

🡪 lack in facial motions, discussion, showing emotions

🡪 like Sophia. Conceivable to work for her

- **However**: people who are more into clear commands
- For clear instructions, predefined problems, situations:

🡪 Pepper’s deployment as instructor conceivable; **but** **not** as a leader

- Different tasks as a leader: lead, manage, solve

🡪 pepper not versatile enough, especially when it comes to change of plans

🡪Written rules or given rules important?

- What is the point of having a roboter as a leader? Isn’t his task not to serve us?

🡪 Doesn’t make sense to replace something which actually works well: would it do a better job as we are doing?

🡪 missing passion

- Tries to be too human:
  - that’s why it will be compared to real humans 🡪 that’s why it fails
  - people won’t trust them nor interact with them
  - Not trustworthy

**Missing: Computation part in robotics**

- Two separate fields: robotics & AI
- Advanced possibilities of mechanics and software: software, AI, mechanics functions should be divided in aspects

🡪 concepts should be found: to work on movements

**Loss on human-leadership side:**

- how can we trust it when it come to the edge?

🡪 Leader has to be held responsible! How can pepper be held responsible for a target?

🡪 juridical-based question: Responsibility in case of mismanagement

- What makes human interactions so special?

🡪 evolution-based behavior

- In movies: just a voice and even a kind of connection, even though there was no physical body. 🡪 How can such a connection be built up to?
