# Supplementary material for: Robot leadership–Investigating human perceptions and reactions towards social robots showing leadership behaviors
Source: PLoS One. 2023 Feb 16;18(2):e0281786. doi: 10.1371/journal.pone.0281786 (PMC9934409; doi:10.1371/journal.pone.0281786)
Supplement: S1 Dataset — (ZIP) [file pone.0281786.s002.zip › Interview_Participant1.doc]

**11 Interview**

**Interviewer:**

Wenn du dir jetzt vorstellen würdest, du arbeitest für Pepper, jetzt so als Grundannahme. Was für Gedanken hast du dir jetzt gemacht, während Pepper mit dir geredet hat? #00:00:17#

**Interviewpartnerin:**

Befremdlich. Also ich habe mir erst gedacht, dass es doch merkwürdig ist. Er ist kindlich durch diesen Roboter, durch die Bewegungen und auch durch die Sprache. Ich glaube, was mich auch ein bisschen gestört hat, ist. Ich habe dann den einen oder anderen / Ich habe nicht alles ganz verstanden, weil ich das nicht irgendwie mitgekriegt habe und ich habe jetzt keine Möglichkeit gehabt oder das Gefühl, fragen zu können, ihn aufhalten zu können. Und wenn ich jetzt arbeiten würde tatsächlich eben: Ja, das wäre mega befremdlich. Ich weiß nicht, ob ich das annehmen könnte auf Anhieb, vor allen Dingen als Führungskraft halt, was er oder sie wäre. #00:01:06#

**Interviewer:**

Weißt du, woran es liegt? #00:01:10#

**Interviewpartnerin:**

Ja, da fehlt halt die Interaktion, die menschliche Interaktion. Und ja, dass er/es/sie halt klein und kindlich wirkt für mich. Ich glaube, das ist halt auch / #00:01:29#

**Interviewer:**

Liegt dann daran? Okay. Wie hat es sich für dich so angefühlt, als dich Pepper so angesprochen hat? #00:01:40#

**Interviewpartnerin:**

Also die Bewegungen haben mich irritiert erst. Das fand ich eigentlich ganz cool auch, also dass er sich überhaupt bewegt. Es kam mir halt eben mehr vor wie eine Präsentation, was es ja auch zum Teil war. Aber nicht jetzt konkret wie eine Anweisung, was es ja auch ein Stück weit war. Ja, also insgesamt, so hatte ich das Gefühl, ich erhalte jetzt eine Präsentation auf einem fancy Gerät. #00:02:08#

**Interviewer:**

Okay. Und die Bewegungen haben dich / also waren gut, aber gestört? #00:02:14#

**Interviewpartnerin:**

Die waren halt eben ungewöhnlich. Zum Teil fand ich es gut, dass er sich bewegt hat, aber es war halt nicht natürlich. #00:02:29#

**Interviewer:**

Was hast du dir gedacht, als Pepper „We do not tolerate any mistakes“ gesagt hat? #00:02:35#

**Interviewpartnerin:**

Ja (lacht). Ja, das habe ich mir auch gedacht: Na ja, du kannst es ja sagen. Dir kann man ja die Fehler programmieren oder ausmerzen durch Programmieren. Ja, klang, wie soll ich sagen? Klang für mich schon ein bisschen fordernd auf jeden Fall. #00:02:59#

**Interviewer:**

Fordernd. Aber jetzt vom Gefühl her: Wie / #00:03:05#

**Interviewpartnerin:**

Ja, also ich habe jetzt da mich nicht ängstlich gefühlt, sondern ich habe mir nur gedacht: Ja, mal schauen. Aber ich habe jetzt da keine Angst oder so verspürt, sondern eher so als eine sachliche Information: Wir dulden keine Fehler. Und habe mich dann halt nur gefragt, ob das auch so umsetzbar ist, so (unv. #00:03:26#)

**Interviewer:**

Was denkst du, wie Pepper auf Fehler reagieren würde? #00:03:32#

**Interviewpartnerin:**

Jetzt ist halt natürlich die Frage, gerade bei diesem Marketingding, wie denn da auch Fehler gemessen werden, denke ich mir halt. Weil das ist ja doch was Kreatives. Also woran mache ich jetzt fest, ob es richtig oder falsch ist? Das ist so der eine Punkt. Ich weiß / also ganz ehrllich, ob ich es dann so ernst nehmen kann. Ich hätte jetzt keine Gefühle, ich glaube, ich hätte keine Gefühle der Angst. Ich weiß nicht, je nachdem, welche Konsequenz jetzt daraus sich ergeben würde. Ja. #00:04:12#

**Interviewer:**

Keine Angst. Vielleicht ein bisschen Respekt oder das dann auch nicht? #00:04:19#

**Interviewpartnerin:**

Also ich glaube, ich würde / also im Gesamtkontext würde ich natürlich keine Fehler machen wollen bei einem Job, das ist ganz klar. Wenn ich dann drauf angesprochen werde, ich überlege halt gerade, wenn ich drauf angesprochen werde, egal von wem: Natürlich macht mir das erst mal zu schaffen. Ob das jetzt eine Pepper oder jemand anderes. Aber ob ich es da eher akzeptieren könnte und respektieren könnte? Ja, wie gesagt, ich glaube, wenn ich das immer noch so als dieses eher Kindliche mit dieser Stimme und mit dieser Größe, die der Roboter hat, verbinde, dann könnte es vielleicht abgemildert sein. #00:05:02#

**Interviewer:**

Pepper hat ja gesagt: „You should document what you do so that I am able to follow your steps and identify mistakes. This will allow me, go give you exact feedback on your work.” Hast du dich jetzt bewusst da dran gehalten bei der Aufgabe? #00:05:26#

**Interviewpartnerin:**

Ja, ich war halt ein bisschen irritiert, wie er mir Feedback geben will. Ich habe dann eher drüber nachgedacht bei dem Satz: Ja, werden wir jetzt die Sachen aufgenommen, gescannt? Also ich habe mich halt gefragt: Wie soll das passieren? Wie kann er oder sie das umsetzen? Das habe ich mich tatsächlich gefragt. Aber jetzt bei der Aufgabenbearbeitung, muss ich gestehen, habe ich jetzt einfach nur Brainstorming gemacht und wollte halt schnell irgendwie / Also da war ich einfach in der Aufgabe drin und habe mir jetzt ehrlich gesagt nicht mehr so viele Gedanken gemacht, wie ich es aufschreibe, dass es verständlich sein könnte. #00:06:03#

**Interviewer:**

Wenn er jetzt sehr strenges Feedback geben würde dir, wie würdest du damit umgehen? #00:06:14#

**Interviewpartnerin:**

Da muss man sich ja echt in die Lage hineinversetzen. Ich glaube, nur von Pepper alleine würde mich das / ich glaube, ich müsste schon wissen, was danach noch kommt, nach diesem Feedback. Also nur halt eben ein strenges Kritisieren von Pepper würde mich, glaube ich, nicht so irritieren, wie wenn ich jetzt einen menschlichen Chef oder Chefin vor mir hätte, definitiv. Also ich glaube, da würde es wirklich darauf ankommen: Was passiert mit dieser Kritik? Wird die jetzt irgendwo gespeichert (lacht kurz), irgendwo hinterlegt, wo sich das dann sammelt und dann von mir ein Bild gemacht wird? Wie wird meine Reaktion aufgenommen? Also ich denke jetzt mal laut, wie so was passieren könnte. Ich glaube, ich würde mir eher Sorgen machen, dass ich dann aufgenommen werde durch den Roboter, noch mehr analysiert werde mit Sachen, die ich vielleicht nicht wirklich bewusst wahrnehme und dass dann ein Gesamtbild über mich geschaffen wird. Aber nur die Kritik von Pepper, so intuitiv, glaube ich, und ich vergleiche ja da ständig mit einer Person, würde ich es halt nicht so, glaube ich, nicht – was heißt: nicht ernstnehmen? Eben wie gesagt, ernst nehmen je nachdem, was danach für eine Konsequenz ist. Aber vom Roboter selber wahrscheinlich nicht ganz so akzeptieren können. #00:07:42#

**Interviewer:**

Also würdest du die Kritik an sich nicht wirklich ernst nehmen, außer es hat halt irgendwie Auswirkungen, wird gespeichert, wird irgendwie / #00:07:51#

**Interviewpartnerin:**

Ich glaube, ja. Ich glaube, ja. Ich glaube, mir fehlt so diese Resonanz halt einfach. Wenn man halt jemandem sagt, wo ich das Gefühl habe, einer Person, die könnte jetzt drauf reagieren, die könnte jetzt grantig werden auf meine Reaktion. Oder die ist auf mich / Also ich glaube, die Emotionen fehlen. Also wenn mir dann jemand sagt: „Du, Jana, das fand ich jetzt echt richtig, richtig schlecht“ und dabei noch die Augenbrauen nach oben zieht oder was auch immer, dann: „(Erschreckenslaut) okay, das ist jetzt echt ernst.“ Aber wenn jetzt halt eben ein Roboter zu mir sagt: „Jana, das war jetzt echt richtig, richtig schlecht“, mit der süßen Stimme, dann, ja, würde ich das wahrscheinlich ein bisschen anders wahrnehmen. #00:08:36#

**Interviewer:**

Wie war dein Gesamteindruck von Pepper? #00:08:40#

**Interviewpartnerin:**

Also erst fand ich es sehr cool, dass Pepper, als ich mich da hingesetzt habe, also sich mit bewegt hat, reagiert hat. Also das fand ich sehr positiv, das hat mich irritiert und erfreut. Aber ja halt ein Roboter, kindlich, ein bisschen kindlich, ja. Also deswegen, im beruflichen Kontext, glaube ich, würde ich mir einen größeren Roboter vorstellen. #00:09:16#

**Interviewer:**

Okay. Also jetzt hauptsächlich von der Größe her oder auch vom Aussehen her? #00:09:20#

**Interviewpartnerin:**

Ja, es ist ein Roboter. Es ist halt nicht / Natürlich, hat keine Beine, aber ein Bein, zwei Arme und ein Gesicht, aber das bewegt sich nicht, macht keine Mimik. Die Augen blinken da ein bisschen, das sind halt keine menschlichen Emotionen. Also ich kann es nicht wirklich auf eine Ebene wie einen Menschen in irgendeiner Form stellen gerade. Noch zu befremdlich dafür. #00:09:48#

**Interviewer:**

Also liegt es eher am Roboter an sich, nicht wirklich die Größe oder / #00:09:52#

**Interviewpartnerin:**

Ja, genau, genau, genau: #00:09:53#

**Interviewer:**

Inwiefern hast du dich jetzt von Pepper motiviert gefühlt beziehungsweise wenn du jetzt bei ihm arbeiten würdest, inwiefern würdest du dich motivieren lassen von ihm? #00:10:07#

**Interviewpartnerin:**

Das ist jetzt echt eine gute Frage. Also ich gehe mal davon aus, wenn ich diesen Job machen möchte, dann brenne ich, also dann ist es auch etwas, was mir Spaß macht. Dann brauche ich ja eigentlich nur noch die Anweisungen und dann reicht mir das ja auch, von Pepper die Anweisungen zu bekommen, was zu tun ist. Inwieweit / Ja, Pepper hat halt jetzt nicht gesagt / Ja, schon diese Bewegungen, diese plötzlichen, das ist schon ganz cool (lacht): Er hört mir zu. Also so „Hey, Jana, wir haben schon in der Vergangenheit so tolle Sachen gemacht“ oder „Wir freuen uns, dass du an Bord bist“. Ja, da kamen jetzt keine Sprüche, die voller Elan waren oder so. Also zumindest ist es nicht so bei mir angekommen, sondern halt eben mehr so dieses Monotone. Und ich glaube, für die Motivation, sich jetzt an die Aufgabe ranzuschmeißen, hätte mir vielleicht so noch ein bisschen die Emotion in der Aussprache gefehlt. #00:11:08#

**Interviewer:**

So ein bisschen Enthusiasmus? #00:11:11#

**Interviewpartnerin:**

Ja, genau, genau. Ja, ich denke schon. Ja. #00:11:15#

**Interviewer:**

Aber an sich ist die Idee jetzt für dich nicht so fremd? Du könntest dir schon vorstellen, dass ein Roboter dir Anweisungen gibt? #00:11:25#

**Interviewpartnerin:**

Ja, das definitiv schon, weil er gibt mir eine Aufgabenstellung. Und das könnte ich schon akzeptieren. Aber ich weiß halt jetzt nicht, ob ich, nur weil ich die Anweisung kriege, ob ich es dann wirklich / ob ich ihn oder sie als Führungskraft empfinde. Ich glaube, das ist so der springende Punkt. Aber die Anweisungen würde ich schon / Dann weiß ich, was zu tun ist und mache das dann, ja. #00:11:55#

**Interviewer:**

Nicht als Führungskraft empfinden, eher so als / #00:11:59#

**Interviewpartnerin:**

Aufgabe. #00:12:01#

**Interviewer:**

Aufgabenträger, -präsentierer? #00:12:04#

**Interviewpartnerin:**

Ja. Ja. #00:12:07#

**Interviewer:**

Fühlst du dich von Pepper wertgeschätzt? #00:12:10#

**Interviewpartnerin:**

Ich fühle mich nicht einmal wirklich wahrgenommen. Also schon, dass ich da bin, aber ich glaube, also so: Das ist eine nette Kollegin, die mag ich. Ich glaube nicht, dass er oder sie so was denkt. Deswegen ist meine Auf / Ja, das kommt jetzt halt drauf an: Wenn ich jetzt meine Aufgabe ganz toll gemacht hätte und er oder sie analysiert das und sagt: „Hey, guten Job gemacht!“ Natürlich freue ich mich dann. Aber ob ich mich dann trotzdem so richtig gewertschätzt fühle? Weil es ist ja: Wie hat Pepper das ausgewertet? Wie gesagt, anhand irgendeines Algorithmus oder Analyse oder was auch immer und dann fehlt mir wahrscheinlich halt immer noch die menschliche Komponente dazu. #00:13:02#

**Interviewer:**

Denkst du, Pepper ist fair zu seinen Teammitgliedern? #00:13:10#

**Interviewpartnerin:**

Das denke ich ziemlich sicher oder kann ich mir sehr gut vorstellen, weil halt eben gerade die Emotionen fehlen. Weil es gibt keine Sympathie oder Antipathie oder „Ich bin heute mal mit dem schlechten Fuß aufgestanden“. Und dann sind sicherlich so Bewertungskriterien wie jetzt bei der Aufgabe sicherlich für alle gleich und können halt nicht beeinflusst werden. Und deswegen, ja, denke ich schon, dass es fairer laufen würde. Ja, definitiv. #00:13:45#

**Interviewer:**

Kannst du Pepper als Führungskraft vertrauen? #00:13:47#

**Interviewpartnerin:**

Vertrauen? Kommt halt drauf an, auf was. Ja, nicht in allen Belangen, denke ich. Wie gesagt, vielleicht eben, was fair zu bewerten – dann ja, wegen den genannten Kriterien vorhin. Aber zu wissen, wann was angebracht ist, wann wer zu motivieren ist, sich darauf zu verlassen, dann eher nein. #00:14:19#

**Interviewer:**

Halt bei der Bewertung, fair zu sein, schon, aber dann etwas / #00:14:26#

**Interviewpartnerin:**

Genau. Da, denke ich, da würde ich drauf vertrauen. Eben, das Thema wäre Vertrauen in Ihre Führungskraft, der ich vertrauen kann, dass sie das korrekt auswertet die Leistung und bewertet. Aber wenn es dann um so das Zwischenmenschliche geht, wir müssen jetzt das ganze Team motivieren, so aus der Luft gegriffen, glaube ich nicht, dass Pepper dann genau differenzieren kann, welches Teammitglied jetzt mehr an die Hand genommen werden muss und welches nicht, um den Projekterfolg zu steigern. #00:14:57#

**Interviewer:**

Dann vielleicht eher bei so eher dynamischen Situationen hat er dann / #00:15:02#

**Interviewpartnerin:**

Ja, ich glaube, das trifft es auch ganz gut: dynamische Situationen. Wie reagiert dann Pepper, wenn halt mal was nicht gut läuft? #00:15:09#

**Interviewer:**

Welche Verhaltensweisen waren dir sympathisch? #00:15:14#

**Interviewpartnerin:**

Ja, obwohl ich das vorhin so stark kritisiert habe, finde ich es trotzdem sehr sympathisch halt das Kindliche, das Süße irgendwie. Wie gesagt, da (unv. #00:15:28#) ich halt echt, wenn mich Pepper so anschaut / Und halt auch diese Bewegungen, die dazukommen; die Kopfbewegungen finde ich auch recht sympathisch, dass sie da auch bestimmte Richtungen, nicht nur oben-unten, sondern halt so ein bisschen schräg und so. Und die Stimme, ja, die ist halt nett, niedlich, sympathisch, ja. #00:15:49#

**Interviewer:**

Was hat dich an seinem Verhalten gestört? #00:15:52#

**Interviewpartnerin:**

Ja, dass ich das Gefühl hatte eben, ich kann jetzt nicht hinterfragen, sondern dass er halt oder sie so durchbrettert: Das ist jetzt halt das und jetzt arbeitet damit. Das empfand ich schon als störend, dass ich nicht finde, dass ich da eine Interaktion mit Pepper haben kann. #00:16:14#

**Interviewer:**

Okay. Wie würde es dir jetzt gehen / #00:16:20#

**Interviewpartnerin:**

Ach, und vielleicht, das ergänze ich noch. Manchmal waren die Armbewegungen dann doch streckenweise zu viel. Das hat mich, also oder hatte ich das Gefühl, das irritiert mich. Jetzt kann ich gar nicht zuhören, weil ich mir denke: Was macht er oder sie jetzt als Nächstes mit den Armen? Nur als Ergänzung. #00:16:35#

**Interviewer:**

Die Armbewegungen waren etwas / Wie würde es dir jetzt gehen, wenn Pepper dein Chef wäre? #00:16:41#

**Interviewpartnerin:**

(überlegt etwas) Ich glaube, zum jetzigen Zeitpunkt könnte ich es noch nicht so ernst nehmen. Also ich würde halt immer daran denken, glaube ich, dass im Hintergrund noch jemand da ist, dass noch ein Mensch da ist und dass Pepper jetzt ein Ausführer ist, also jemand, der die Anweisungen von einem menschlichen Chef ausführt. Verstehst du, wie ich meine? #00:17:15#

**Interviewer:**

Du stellst dir halt vor, dass hinter Pepper noch jemand da ist, der ihm sagt, was er machen soll. #00:17:21#

**Interviewpartnerin:**

Genau. Der oder die mir dann final einen auf den Deckel gibt (lacht), wenn ich die Sachen nicht ausführe, wie Pepper sagt. Also so als Zwischenglied eben. Ja. #00:17:41#

**Interviewer:**

Und was meinst du, was da gut oder schlecht funktionieren würde? #00:17:46#

**Interviewpartnerin:**

Also glaube, was ich mir echt gut vorstellen kann, ist, dadurch, dass Pepper eben so nüchtern ist, kann ich mir vorstellen, dass / Ich glaube, ich würde selbständiger arbeiten. Ich würde vielleicht nicht so viel hinterfragen, weil ich eben das Gefühl habe, da gibt es eh nicht so viel Interaktion. Ich glaube, ich würde Pepper halt eben als Anweisungsträger und Beurteiler sehen, der eben meine Leistungen fair beurteilt und mir die Anweisungen übermittelt. Aber nicht die Interaktion dazwischen. das heißt, wie ich jetzt was genau mache, würde ich, denke ich, da viel eher selber entscheiden als wenn ich jetzt eine menschliche Führungskraft vor mir hätte, wo ich mehr rückfragen würde. Das glaube ich jetzt so intuitiv. Das fände ich zum Beispiel dann ganz cool irgendwie, diese Selbständigkeit zu haben. Ja, aber halt eben, dass die Schattenseite / Aber ich weiß es nicht, vielleicht kann ja Pepper auch interagieren. Das weiß ich jetzt natürlich nicht. #00:18:57#

**Interviewer:**

Er kann schon interagieren. Momentan wegen Experimente müssen wir halt so ziemlich standardisiert das machen. #00:19:03#

**Interviewpartnerin:**

Ja. Aber wenn Pepper interagieren würde, ich glaube, ich würde halt so eine Umstellungszeit brauchen, um / Also ich würde mich auf jeden Fall drauf einlassen, aber es wäre natürlich mega ungewöhnlich, das ist jetzt nicht so: „Hier ist deine neue Chefin.“ Ja, das wäre für mich dann ganz natürlich, wenn es ein Mensch ist. Und das ist jetzt eine ganz neue Situation dann auch, da muss man sich drauf einlassen. #00:19:31#

**Interviewer:**

So vom Gefühl her würdest du nicht so viel nachfragen, sondern er gibt ein Ziel und dann würdest du halt mehr oder weniger autonom darauf hinarbeiten? #00:19:43#

**Interviewpartnerin:**

Wahrscheinlich, wahrscheinlich, ja. Aber ich kann mir halt auch vorstellen, dass Pepper durch die Büros fährt und Fragen stellt und „Wie geht es denn so?“. Also ich glaube, dass ich mich dann schon auf so eine Konversation einlassen würde. Ich würde Pepper jetzt da nicht irgendwie vor offener Tür dann da stehen lassen und gar nicht drauf reagieren, also überhaupt nicht. Da würde ich mich, glaube ich, schon drauf einlassen. Aber der Punkt ist halt eben der Punkt mit der Führungskraft, mit dem nach oben blicken, wenn ich doch eigentlich bei Pepper gerade mehr nach unten blicke. Wobei natürlich eben Führungskräfte ja heutzutage / ich schätze es ja auch sehr, wenn man natürlich auf Augenhöhe arbeitet und nicht hierarchisch. Das wäre dann eben so ein Punkt, jetzt denke ich halt so ein bisschen laut, wo Pepper vielleicht ein Vorteil wäre, weil man auf Anhieb denken würde: Wir sind auf Augenhöhe. Das könnte vielleicht ein wesentlicher Vorteil sein, dass man nicht dieses Chefgefühl tatsächlich hat, sondern da habe ich jetzt ein Teammitglied, einen Kooperationspartner, einer, der mit mir das erarbeitet. Ja. Könnte schon laufen, ja (lacht kurz). #00:21:01#

**Interviewer:**

Wie gut, meinst du, kann Pepper für die Zukunft seines Teams Pläne erstellen und Ziele setzen? #00:21:16#

**Interviewpartnerin:**

(überlegt etwas) Also das Gesamtziel und den Gesamtplan auf jeden Fall gut, wenn man ihn halt eben strukturiert erstellen kann. Ich glaube, das kann Pepper sicherlich sehr gut machen, sehr sukzessiv und pragmatisch und viel Drumherum zu überlegen, wie es vielleicht ein Mensch machen würde. Aber auch hier wieder eben das angepasst. Ich glaube nicht, dass Pepper eben sich an das einzelne Teammitglied so anpassen kann, sondern das als Gesamtes betrachtet und somit die Gesamtsituation gut erfassen kann, in Ziele formulieren kann. Aber dann individuell zu sagen: Okay, dieses Ziel passt am besten jetzt zur Biljana“, glaube ich nicht, dass das Pepper tun könnte. Und so ein gemeinsames Projekt ist ja nicht nur die Ziele abarbeiten als Ziel, sondern halt dann auch wie, also Weg, gemeinsamen. Und das könnte ich mir dann schwierig vorstellen. #00:22:28#

**Interviewer:**

Also kannst du dir auch vorstellen, dass er so einen Plan erstellt, so einen allgemeinen für das ganze Team. Aber dann die einzelnen Aufgaben aufteilen und vergeben, ist dann / #00:22:38#

**Interviewpartnerin:**

Ja, das kann er dann schon auch. Aber ob dann die Leute glücklich sind mit den Aufgaben, wie sie sind und vor allen Dingen, wie die Aufgaben laufen. Was ist denn, wenn es dann jetzt irgendwie Probleme gibt oder / Also nicht nur fachliche, sachliche Probleme wie zum Beispiel die Lieferung der Sensoren kommt nicht. Dann kann Pepper sicherlich reagieren. Aber was ist, wenn ich sage: „Boah, ehrlich gesagt, ich kann es nicht mehr sehen. Ich will mit diesem Einzelthema nichts mehr zu tun haben und ich habe irgendwelche Probleme zu Hause, die trage ich jetzt mit in die Arbeit.“ Und hier flexibel zu reagieren und zu schauen, dass die Teammotivation insgesamt und die Grundstimmung im Team passt mit der Aufgabenverteilung: Ich glaube, das kann Pepper nicht erfassen. #00:23:22#

**Interviewer:**

Das sind dann so die weichen Faktoren, die er dann / #00:23:23#

**Interviewpartnerin:**

Ja, genau, genau. #00:23:26#

**Interviewer:**

Okay. Was würdest du jetzt vermuten: Wie erfolgreich wäre ein Team, das von Pepper geführt wird? #00:23:36#

**Interviewpartnerin:**

Jetzt halt nach dem ganzen vielen Reden, das Für und Gegen: Ich wäre halt total gespannt, wie es funktioniert im Vergleich zu einem Menschen. Ich glaube, dass bestimmte Sachen besser laufen könnten, erfolgreicher und andere Sachen halt eben scheitern können. Also ich kann nicht sagen zum jetzigen Zeitpunkt, wird erfolgreicher laufen oder weniger erfolgreich. Wir vergleichen jetzt wieder mit einer menschlichen Führungskraft. Ich glaube, da kommt es komplett drauf an, welche Aufgaben es sind. #00:24:19#

**Interviewer:**

Okay. Was für Aufgaben meinst du, würden gut funktionieren, was eher schlecht? #00:24:23#

**Interviewpartnerin:**

Ja, zielorientiert, klare Aufgaben, die man halt eben auch klar definieren kann: Bis dahin ist es fertig und das abhaken. Dass Pepper dann eben vielleicht kontinuierlich das einfordert von den Mitarbeitern, diese Ziele zu erfüllen in einer nüchternen Art, kann vielleicht eben ein Vorteil sein. Statt eben, dass man diesen Druck empfindet von einer menschlichen Führungskraft. Glaube ich, das könnte ein Vorteil sein, dass man das eben ein bisschen besser erreicht, mit einem besseren Gefühl. Aber auch hier wieder: Woran könnte es scheitern? Dass Pepper sich halt nicht auf irgendwelche Emotionen einstellen kann. Pepper braucht keinen Schlaf und ist nicht müde in der Früh, aber es kann halt sein, dass ich manchmal die halbe Nacht wach war, weil irgendwas passiert ist und ich dann nicht die Leistung bringen kann. Eine menschliche Führungskraft kann das dann vielleicht verstehen, akzeptieren und dann halt spontan Aufgaben an Andere delegieren. Pepper würde das vielleicht nicht so tun. Ich glaube, das ist so dieser Punkt, der den Teamspirit dann beeinflussen kann und somit dann auch das Gesamtergebnis. #00:25:43#

**Interviewer:**

Also meinst du, er hätte dafür dann auch kein Verständnis, wieso man jetzt müde ist und / #00:25:49#

**Interviewpartnerin:**

Genau, das würde er oder sie gar nicht erkennen. Also das glaube ich auf gar keinen Fall, wenn ich es nicht aktiv anspreche. Aber manchmal sagt man halt nicht Sachen. Und Pepper würde diese vibes nicht erkennen, während dann eine menschliche Führungskraft (unv. #00:26:05#) „Boah, Biljana, heute bist du wohl anscheinend nicht so fit. Wir haben aber morgen eine Abgabe. Wenn es dir nicht gut geht, dann lass uns mal schnell umdisponieren, damit wir dann diesen Erfolg erzielen.“ Also das glaube ich halt eben: Solange es klar sachlich, fachlich ist und keine großen, ich sag jetzt mal, emotionalen Probleme auftreten in der Bearbeitung, glaube ich, ist Pepper weiter vorne. Aber sobald es dann eben solche emotionalen Schwankungen gibt, wo man eben Sachen anpassen muss, da könnte Pepper verlieren. #00:26:37#

**Interviewer:**

Das könnte er Probleme haben? #00:26:37#

**Interviewpartnerin:**

Ja. #00:26:38#

**Interviewer:**

Dann sind wir jetzt auch schon durch. Ein paar Fragen zu dir. Wie alt bist du? #00:26:45#

**Interviewpartnerin:**

36. #00:26:48#

**Interviewer:**

Arbeitest als? #00:26:52#

**Interviewpartnerin:**

Promotionsstipendiatin, Stipendiatin. #00:26:56#

**Interviewer:**

Was für einen Studiengang, was für eine Richtung? #00:27:02#

**Interviewpartnerin:**

Wirtschaftswissenschaften. #00:27:06#

**Interviewer:**

Hattest du schon mal Erfahrungen mit Robotern, AI, solche Sachen? #00:27:16#

**Interviewpartnerin:**

Nein. #00:27:19#

**Interviewer:**

Jetzt noch zum day briefing. Es gibt (unv. #00:27:24#) mal nicht.

**Interviewpartnerin:**

Bitte was? #00:27:25#

**Interviewer:**

Pocket gardener, diese startups gibt es nicht. #00:27:29#

**Interviewpartnerin:**

Okay, das dachte ich mir, ja. #00:27:29#

**Interviewer:**

Und ja, Pepper ist auch noch nicht so weit. Er kann eigentlich nur diese Präsentation bis jetzt. Er kann ganz einfache Fragen beantworten, funktioniert aber auch nicht immer. Und wir untersuchen halt, wie ein Roboter als Führungskraft wahrgenommen wird, ob es auch akzeptiert wird. Was gut funktionieren könnte, was schlecht, solche Sachen. #00:27:59#

**Interviewpartnerin:**

Ja, cool. Megaspannend. #00:28:01#

**Interviewer:**

Ja, schon. #00:28:01#

**Interviewpartnerin:**

Ja, ich bin mal echt gespannt. Heute habt ihr erst gestartet, oder? #00:28:05#

**Interviewer:**

Mit den Experimenten mit Pepper an sich schon. Ich habe davor noch mit einem Video von Pepper halt ein paar Experimente gemacht. Aber jetzt mit Pepper an sich ist es halt schon anders. #00:28:18#

**Interviewpartnerin:**

Oh, cool! Das machst du für deine Masterarbeit? #00:28:20#

**Interviewer:**

Nein, Bachelor. #00:28:23#

**Interviewpartnerin:**

Da hast du dir ein bisschen was vorgenommen. #00:28:25#

**Interviewer:**

Ja, schon, schon. #00:28:26#

**Interviewpartnerin:**

Aber mit Silvie hast du ja die perfekte Mentorin. Also machst du alles mit Silvie oder ist da noch jemand? #00:28:33#

**Interviewer:**

Nein, also ich mache nur mit Sylvia, aber das Projekt ist ja ein größeres Projekt. Das wird, ich glaube, mit Bundeswehruni zusammen gemacht. #00:28:43#

**Interviewpartnerin:**

Ach, irgendwas hat sie mal erwähnt, ja. Ja, das kann sein. Ach so, aber ich wusste nicht, dass es mit dem Roboter zu tun hat. Aber wo kommt der Roboter eigentlich her? #00:28:54#

**Interviewer:**

Also der Roboter an sich ist eine französisch-japanische Firma, die den produziert. Wird auch schon in Geschäften und so eingesetzt, ja so als Gag und Kundenservice und solche Sachen. #00:29:13#

**Interviewpartnerin:**

Ah, okay. Aber wo dann? Auch in Deutschland oder in Frankreich? #00:29:19#

**Interviewer:**

Also eher Japan, glaube ich. Japan ist ja eher mit Robotern. Ich habe schon gehört, dass er auf mehreren Messen unterwegs ist und dann halt Sachen präsentiert, so ungefähr wie jetzt. Und solche Sachen halt. #00:29:33#

**Interviewpartnerin:**

Aber so im Führungskontext wird da noch nichts groß ausprobiert? #00:29:36#

**Interviewer:**

Nein. #00:29:38#

**Interviewpartnerin:**

Ja, das denke ich mir auch. Ich bin sehr / #00:29:40#

**Interviewer:**

Ist auch von der Literatur her sehr / also gibt es eigentlich nichts. #00:29:43#

**Interviewpartnerin:**

Klar, ja, kann ich mir gut vorstellen, dass es da nichts gibt. Ja, megaspannend. Was ist deine Forschungsfrage, also insgesamt gerade? #00:29:51#

**Interviewer:**

Eigentlich eben die perceiption oder halt Akzeptanz von Robotern als Führungsperson. #00:30:00#

**Interviewpartnerin:**

Schwierig. #00:30:01#

**Interviewer:**

Ja, genau. Also das ist auch so die Richtung, wo wir gerade hingehen. #00:30:07#

**Interviewpartnerin:**

Die ihr auch hypothetiviert habt. #00:30:08#

**Interviewer:**

Ja, ist halt befremdlich ein bisschen und auch schwierig zum Vorstellen. #00:30:13#

**Interviewpartnerin:**

Ja, cool. Dann viel, viel Erfolg, viel Spaß. Ich werde bestimmt mitkriegen, wie es weiter läuft. Auf jeden Fall war es sehr kurzweilig, hat mir sehr viel Spaß gemacht. #00:30:26#

**Interviewer:**

Vielen Dank. #00:30:27#

**Interviewpartnerin:**

Ja, gerne.
