# Supplementary material for: Robot leadership–Investigating human perceptions and reactions towards social robots showing leadership behaviors
Source: PLoS One. 2023 Feb 16;18(2):e0281786. doi: 10.1371/journal.pone.0281786 (PMC9934409; doi:10.1371/journal.pone.0281786)
Supplement: S1 Dataset — (ZIP) [file pone.0281786.s002.zip › Interview_Participant2.doc]

**12 Interview**

**Interviewer:**

So als Prämisse: Stell dir vor, du arbeitest für Pepper jetzt. Was für Gedanken sind dir durch den Kopf gegangen, während Pepper jetzt mit dir geredet hat? #00:00:15#

**Interviewpartner:**

Zuerst, dass seine Stimme ziemlich unnatürlich klingt und er ist auch schwer zu verstehen, also man muss sich schon ziemlich anstrengen. Klar, es war jetzt auch auf Englisch, aber trotzdem war es schon schwer, ihn zu verstehen. Und ich habe dann auch irgendwie auf seine Augen geachtet, also die verändern sich halt gar nicht so wie bei einem Menschen. Und ja, insgesamt, es klingt halt schon alles noch sehr künstlich. #00:00:42#

**Interviewer:**

Also eher auf die Stimme geachtet? #00:00:44#

**Interviewpartner:**

Mhm (zustimmend). #00:00:46#

**Interviewer:**

Und so auf den Roboter, also auf ihn als Roboter. #00:00:50#

**Interviewpartner:**

Ja. #00:00:50#

**Interviewer:**

Wie hat es sich für dich angefühlt, während Pepper mit dir gesprochen hat? #00:00:55#

**Interviewpartner:**

Ja, irgendwie hätte ich gedacht, es wäre komischer, wenn ein Roboter vor mir steht und mit mir redet. Also meine Erwartungen waren jetzt eigentlich, dass es sich irgendwie anders anfühlt. Es war jetzt nicht so, dass ich mir gedacht habe: Boah, megakrass, da steht jetzt ein Roboter vor mir. Aber vielleicht auch, weil ich letztens so ein Video gesehen habe, da war halt ein Roboter in einem Hotel als Rezeptionist quasi und vielleicht habe ich, weil ich das vor kurzem erst gesehen habe, hat mich das irgendwie nicht so abgeschreckt. Aber irgendwie denkt man sich dann schon so / Man macht sich zumindest schon mal Gedanken und denkt sich so: Wie wäre es jetzt, wenn das wirklich so wäre, wenn eine Führungskraft oder mein Chef ein Roboter wäre? #00:01:38#

**Interviewer:**

Und wie meinst du, wie es für dich wäre? #00:01:41#

**Interviewpartner:**

Ja, ich habe dann gedacht: Wie wäre es , wenn ich zum Beispiel eine Aufgabe hätte und die nicht erledigen könnte und, keine Ahnung, dann hast du ja, wenn du jetzt eine wirkliche Person hast, dann hast du irgendwie so Gewissensbisse, wenn du jetzt was nicht erledigen kannst. Und bei so einem Roboter denkst du dir dann halt: Scheiß drauf, ist nur ein Roboter. Und, keine Ahnung, ich glaube, manchmal wäre das halt so eine Sache. Und ja, ich meine, es ist halt schon irgendwie komisch. Also ich kann mir nicht wirklich vorstellen, dass ich mal von einem Roboter kommandiert werde oder als Chef habe. #00:02:21#

**Interviewer:**

Also wenn er dir eine Aufgabe gibt, würdest du die also vielleicht schon machen, aber wenn es zu schwierig ist, dann auch nicht. #00:02:32#

**Interviewpartner:**

Ja, also ich glaube, einen Roboter könnte ich nicht so richtig ernstnehmen wie jetzt wirklich einen Menschen. #00:02:38#

**Interviewer:**

Weißt du, wieso? #00:02:44#

**Interviewpartner:**

Ja, zum Beispiel wenn du jetzt einen Menschen als Chef hast, der kann ja dann theoretisch auch zu einem Anderen gehen, den er kennt oder zum nächst höheren Vorgesetzten und kann sagen: „Ja, hier, der Herr Knoblich, der arbeitet aber gar nicht gut“ und so. Und ich weiß nicht, ob ein Roboter / Also ich glaube, den können auch andere Leute nicht so ernstnehmen, zumindest jetzt. Also vielleicht hat sich das in zehn oder 20 Jahren dann auch geändert, aber irgendwie ich kann es mir gerade nicht vorstellen. #00:03:11#

**Interviewer:**

Welche Gedanken hast du dir gemacht, als Pepper gesagt hat: „My vision ist hat we as them marketing department can make people appassionate about our product.“?

**Interviewpartner:**

Also ja, ich habe mir halt irgendwie gedacht: Okay, das kommt jetzt nicht wirklich von Pepper, sondern da hat halt irgend jemand da ihm quasi vorgegeben, dass er das sagen soll. Aber ich kann mir jetzt nicht so wirklich / Ja, so ein bisschen auswendig gelernt rüber kam es halt. Aber es war jetzt nicht so wirklich mit Leidenschaft. #00:03:45#

**Interviewer:**

Also wie er es gesagt hat, war es für dich halt etwas / nicht so leidenschaftlich? #00:03:54#

**Interviewpartner:**

Ja. Ja. #00:03:55#

**Interviewer:**

Wie gut hat Pepper seine Vision für die Zukunft definiert, meinst du? Oder wie gut kann er sie definieren? #00:04:06#

**Interviewpartner:**

Weiß ich gar nicht mehr genau, was er gesagt hat. #00:04:10#

**Interviewer:**

Es war ja das mit, dass er mit seinem Marketing Department „make people passionial about our products.” Und wie meinst du, wie er so eine Vision definiert, definieren kann? #00:04:30#

**Interviewpartner:**

Also an sich finde ich halt schon mal das Produkt / weiß ich nicht, was ich von dem Produkt halten soll, ehrlich gesagt, ob ich davon so überzeugt bin. Von daher fand ich es dann auch schwierig, an die Vision von Pepper zu glauben. #00:04:43#

**Interviewer:**

Okay. Und was meinst du: Kann er eine Vision für die Zukunft definieren? #00:04:51#

**Interviewpartner:**

Jetzt von sich aus, dass er von sich aus eine Definition oder eine Vision / #00:04:56#

**Interviewer:**

Ja. #00:04:57#

**Interviewpartner:**

Ja, also ich traue es ihm schon zu, dass er halt auf Basis von irgendwelchen Algorithmen oder so dann eine Vision formuliert, kann schon sein, ja. Kann ich mir vorstellen. #00:05:09#

**Interviewer:**

Pepper hat auch gesagt: „As a leader I always want to be a role model.“ Was hältst du davon? Inwiefern meinst du, dass er ein Vorbild für seine Mitarbeiter oder für dich sein kann? #00:05:21#

**Interviewpartner:**

Na ja, das glaube ich jetzt nicht wirklich. Also für mich ist ein role model halt auch gerade so im zwischenmenschlichen Bereich ein role model und einen Roboter kann ich mir da jetzt nicht vorstellen. #00:05:33#

**Interviewer:**

Also fehlt bei dir so das Zwischenmenschliche? #00:05:38#

**Interviewpartner:**

Ja, also stell dir mal vor, ein Roboter geht ja nicht mit dir in die Kantine oder so. Das ist ja / oder der Roboter erzählt dir nicht, was er am letzten Wochenende gemacht hat. Und ja, also gerade auch halt, wenn so / vielleicht könnte Pepper auch mal irgendwann Stresssituationen oder Konfliktsituationen im Unternehmen lösen. Aber ich meine, ich kann mich jetzt nicht an einem Roboter orientieren. Ich orientiere mich an anderen Menschen, aber nicht an einem Roboter. #00:06:11#

**Interviewer:**

Okay. Also auch nicht bei der Arbeit irgendwie, wie man arbeiten kann, soll? #00:06:19#

**Interviewpartner:**

Na ja, weiß ich nicht. Weil ich wahrscheinlich immer denken würde: Ja, das hat jetzt ein Roboter gemacht. Aber ich bin kein Roboter, von daher / also vielleicht kann man schon was von ihm lernen, aber so ein richtiges Vorbild, glaube ich, kann er für mich nicht sein, weil ich halt immer denke, ein Roboter ist halt kein Mensch und das ist ein Computer. Der wird nie müde, der kann auch, keine Ahnung, wenn er schon zehn Stunden gearbeitet hat, kann er die nächste Aufgabe immer noch perfekt machen wahrscheinlich. Und als Mensch kannst du es halt nicht und von daher ist er jetzt für mich auch nicht so ein role model. #00:06:51#

**Interviewer:**

Also für dich ist er zu unterschiedlich, zu / #00:06:52#

**Interviewpartner:**

Ja. #00:06:54#

**Interviewer:**

Wenn du jetzt in einem Team wärst mit Pepper als Führungskraft, würdet ihr gemeinsame Ziele anstreben, die von Pepper gesetzt werden oder eher nicht? #00:07:13#

**Interviewpartner:**

Ja, also ich kann mit schon vorstellen, dass wir dann gemeinsame Ziele anstreben. Aber ich würde halt immer irgendwie denken: Ja, hat er irgendwelche Fehler gemacht oder ist der falsch programmiert? Und haben wir irgendwas vergessen, woran Pepper jetzt nicht gedacht hat, einfach weil es halt ein Computer ist? Also ich würde immer noch so selber für mich überlegen: Ja, ist das alles richtig, was Pepper macht? #00:07:39#

**Interviewer:**

Also würdest du nicht wirklich vertrauen, dass er 100 % richtig ist? #00:07:44#

**Interviewpartner:**

Nein, 100 % nicht. #00:07:45#

**Interviewer:**

Inwiefern hast du das Gefühl, dass Pepper hohe Erwartungen an dich als Teammitglied oder an das Team hat? #00:07:56#

**Interviewpartner:**

Also dadurch, dass er halt wahrscheinlich auf Erfolg programmiert ist, denke ich schon, dass er hohe Erwartungen hat an die Teammitglieder. #00:08:08#

**Interviewer:**

In welchem Ausmaß glaubst du, dass Pepper dich als Teammitglied angemessen in deiner Arbeit unterstützen kann? #00:08:19#

**Interviewpartner:**

Ja – schwierig. Also ich weiß ja gar nicht, was der noch jetzt so weiter kann. Also wenn ich ihm jetzt zum Beispiel eine Frage stellen würde, weiß ich ja gar nicht, was er mir drauf antworten könnte. Oder wenn ich jetzt / Ich glaube, es kommt vielleicht auf die Frage an. Vielleicht fachliche Sachen kann er vielleicht ganz gut, aber wenn ich jetzt irgendwie auf der Arbeit wäre und fragen müsste: „Wer arbeitet gerade an welchem Projekt oder wenn kann ich dazu fragen?“ oder so, ich weiß nicht, ob er mir da halt / Ich denke, es gibt bestimmte Bereiche, wo er helfen kann, aber nicht so viele. #00:09:10#

**Interviewer:**

Also eher sachliche Sachen? #00:09:13#

**Interviewpartner:**

Ja. #00:09:15#

**Interviewer:**

Und wo könnte er gar nicht helfen? #00:09:23#

**Interviewpartner:**

Ich weiß nicht, zum Beispiel wenn man jetzt irgendwie was absprechen muss, sagen wir, so eine deadline, ob man die jetzt noch verlängern könnte. Manchmal muss man ja so Sachen einschätzen. Oder auch, wenn es ums Priorisieren geht und dann vielleicht mal sich auch so aufs Bauchgefühl / was jetzt wichtiger ist. Und ja, ich weiß nicht, ob er das so gut könnte zum Beispiel. #00:09:55#

**Interviewer:**

Also könnte er solche Sachen nicht wirklich einschätzen? #00:09:58#

**Interviewpartner:**

Ja. #00:10:00#

**Interviewer:**

Inwiefern hast du das Gefühl, dass sich Pepper auf dich und andere anpasst? #00:10:09#

**Interviewpartner:**

Also ich habe mit dem ja bis jetzt noch nicht gesprochen. Von daher, ich weiß es gar nicht. Spricht er nur Englisch? #00:10:20#

**Interviewer:**

Ja. Momentan ist er ziemlich eingeschränkt wegen einem Experiment, weil es halt standardisiert ist. Aber / #00:10:28#

**Interviewpartner:**

Ja, also ich denke schon, dass er lernfähig ist relativ schnell. Also ich glaube, da ist die Technik schon ziemlich weit, dass er sich anpasst. Also kann ich mir schon vorstellen, ja. #00:10:40#

**Interviewer:**

Und dann auch Rücksicht auf persönliche Bedürfnisse nimmt? Solche Sachen? #00:10:44#

**Interviewpartner:**

Was meinst du mit persönlichen Bedürfnissen? #00:10:49#

**Interviewer:**

Ich weiß nicht. Zum Beispiel, wenn ich mehr Betreuung brauche als ein Kollege oder ja, solche Sachen halt. Es ist ja jeder irgendwie individuell, jeder braucht was anderes. Meinst du, (unv. #00:11:05#)

**Interviewpartner:**

Ja, doch, denke ich schon, dass er das merkt dann nach einer Zeit. #00:11:09#

**Interviewer:**

Inwiefern hat dich jetzt Pepper angeregt, neue und innovative Ideen zu produzieren? #00:11:17#

**Interviewpartner:**

Selber zu produzieren nicht so, aber es ist auf jeden Fall so, dass ich jetzt mehr drüber nachdenke. Also ich habe vorher ja auch noch nie mit so einem Roboter zu tun gehabt, aber es hat mich auf jeden Fall jetzt schon mal so angeregt, irgendwie zu überlegen, ob das wirklich irgendwann mal kommt oder wie das für mich wäre. Von daher: Ein bisschen hat es mich schon beeinflusst. Aber ich glaube, so dass ich produktiver werde oder so / also ich meine, Roboter sind halt eben nicht so meins oder dieses Technische. Von daher, ja, also ich glaube, jetzt groß innovativer werde ich / Vielleicht wenn ich mehr Kontakt mit ihm hätte, aber das war halt jetzt auch relativ kurz. #00:12:04#

**Interviewer:**

Und wenn du dir jetzt vorstellst, du würdest jetzt für ihn arbeiten: Würdest du da dich angeregt fühlen, Ideen zu produzieren? Also wenn er dich was fragt, wärst du das kreativer, würdest du da, weiß nicht, also eher Neuigkeiten dir einfallen lassen? #00:12:30#

**Interviewpartner:**

Es kann, vielleicht, weil er vielleicht Fragen anders stellt als jetzt ein Mensch, einfach weil er anders programmiert ist. Kann sein, dass ich dann auch anders angeregt bin halt, mir Sachen zu überlegen. Aber ansonsten weiß ich nicht, ist schwierig. #00:12:51#

**Interviewer:**

Denkst du, Pepper würde dich ermutigen, den Status quo anzuzweifeln? Also wie momentan Sachen gemacht werden, vielleicht auch Arbeitsabläufe, solche Sachen. #00:13:07#

**Interviewpartner:**

Kann schon sein, weil bis jetzt habe ich es halt nicht angezweifelt oder man denkt sich halt so: Ja, okay, so wie es jetzt ist mit den Vorgesetzten und alles und Chefs, ist es halt so. Aber klar, wenn man jetzt mit so einem Roboter gesprochen hat, dann denkt man sich vielleicht: Na ja. Auch wenn ich mir jetzt nicht wirklich vorstellen könnte, mit einem Roboter zusammenzuarbeiten: Das Eine oder Andere kann man vielleicht trotzdem irgendwie übernehmen. #00:13:35#

**Interviewer:**

Und wenn du jetzt für ihn arbeiten würdest, würdest du da auch anfangen, vielleicht irgendwelche Arbeitsabläufe oder eine Art zu arbeiten, anzuzweifeln oder / #00:13:47#

**Interviewpartner:**

Kann schon sein, dass er zum Beispiel irgendwelche Projektmanagement oder so / dass er da Sachen anders angeht und dann würde ich mir vielleicht auch überlegen: Okay, vielleicht kann man das ja wirklich anders machen. #00:14:03#

**Interviewer:**

Würdest du für Pepper beziehungsweise um die Ziele von Peppers Abteilung zu erreichen, mehr als das zwingend Notwendige tun? Also würdest du nur das machen, was erwartet wird oder ein bisschen mehr? #00:14:23#

**Interviewpartner:**

Also ich glaube, ich würde es nicht für Pepper machen, aber ich würde es für die Abteilung machen. Weil Pepper ist halt nur ein Roboter. Selbst wenn der geschimpft bekommt, dann wäre es mir relativ egal, aber ich würde es halt für die Abteilung machen. #00:14:45#

**Interviewer:**

Also wären irgendwie Konsequenzen für den Roboter dir eigentlich ziemlich egal? #00:14:49#

**Interviewpartner:**

Ja. Ich glaube jetzt nicht, dass ich so eine emotionale Beziehung zu ihm aufbauen würde, dass es mir weh tun würde, aber für die Abteilung halt. #00:14:57#

**Interviewer:**

Damit die Abteilung halt erfolgreich wird? #00:15:00#

**Interviewpartner:**

Mhm (zustimmend). #00:15:01#

**Interviewer:**

Du bist dann eher ein Teamplayer? #00:15:04#

**Interviewpartner:**

Ja. #00:15:06#

**Interviewer:**

Wie war den Gesamteindruck von Pepper? #00:15:08#

**Interviewpartner:**

Ja, insgesamt glaube ich, dass er schon noch ein bisschen / es braucht noch ein bisschen Arbeit an Pepper, also Programmierung und so. Also hat er jetzt auch gerade immer wieder so dazwischengesprochen und so. Ja, also es ist halt auch allein schon, wenn man sich den so anguckt: Ich glaube, wenn man jetzt so einen Roboter bauen würde, der halt schon mal aussieht wie ein Mensch, dann würde schon mal viel wegfallen von diesem, dass man denkt: Okay, das ist halt jetzt irgendwas voll Künstliches. Und ja, wie der halt dasteht, sieht er eher aus wie aus so einem Science-Fiction-Film. Ja, insgesamt wirkt es für mich nicht so komplett ausgereift. #00:15:53#

**Interviewer:**

Also du würdest dir „wünschen“, dass er menschlicher aussieht? #00:16:00#

**Interviewpartner:**

Ja. #00:16:01#

**Interviewer:**

Inwiefern hast du dich von Pepper motiviert gefühlt? #00:16:07#

**Interviewpartner:**

Nicht so. Also ja, vielleicht lag es halt echt daran, dass seine Stimme noch relativ emotionslos war. Es klang irgendwie halt relativ so plastikmäßig, so künstlich und computermäßig. Deswegen war es jetzt für mich nicht so mega motivierend. #00:16:29#

**Interviewer:**

Liegt es hauptsächlich an der Stimme oder auch daran, dass er eben ein Roboter ist und es dir / #00:16:38#

**Interviewpartner:**

Ja, vielleicht. Wenn da jetzt eine hübsche, junge Dame stehen würde, dann wäre ich vielleicht anders motiviert. #00:16:45#

**Interviewer:**

Fühlst du dich von Pepper wertgeschätzt? #00:16:49#

**Interviewpartner:**

(zögernd) Ja. Ja, einigermaßen. Also für die relativ kurze Zeit, denke ich, hat er mir schon zumindest den Eindruck vermittelt, dass er mich so respektiert. #00:17:07#

**Interviewer:**

Und wie hat er das gemacht, also wieso hast du das Gefühl? #00:17:11#

**Interviewpartner:**

Weil ich glaube, er hat in der Ansprache ein paar Mal „we“ gesagt, also wir, so das Gemeinschaftsgefühl ein bisschen gestärkt. Von daher, ja, habe ich mich so ein bisschen angesprochen gefühlt. #00:17:23#

**Interviewer:**

Also dass er so inklusiv ist? #00:17:27#

**Interviewpartner:**

Mhm (zustimmend). #00:17:27#

**Interviewer:**

Denkst du, Pepper ist fair zu seinen Mitarbeitern? #00:17:32#

**Interviewpartner:**

ja, also ich glaube, er ist immer nur so fair, wie er programmiert ist. #00:17:38#

**Interviewer:**

Okay (lacht kurz). #00:17:40#

**Interviewpartner:**

Also ja, ich denke, auf der Basis halt wirklich, wie er programmiert ist, denke ich, / Ich glaube jetzt nicht, dass er ungerecht ist. Und er ist ja jetzt auch nicht von Emotionen geleitet, sondern er ist ja eigentlich nur rein auf der sachlichen Ebene. Von daher glaube ich schon, dass er fair ist, ja. #00:18:00#

**Interviewer:**

Also dadurch, dass er so nüchtern eigentlich ist? #00:18:03#

**Interviewpartner:**

Genau, ja. Vielleicht manchmal zu sachlich. #00:18:08#

**Interviewer:**

Wieso? #00:18:08#

**Interviewpartner:**

Na ja, ich meine, es gibt ja manchmal Entscheidungen, da muss man vielleicht das Sachliche irgendwie mal außen vor lassen und mehr so in die emotionale Schiene, da irgendwie Entscheidungen treffen. Und vielleicht ist er da manchmal zu sachlich und trifft dann die falschen Entscheidungen, wo man dann in der einen Situation besser die andere getroffen hat. #00:18:31#

**Interviewer:**

Hättest du da ein Beispiel oder nicht? #00:18:34#

**Interviewpartner:**

Ich weiß nicht, das ist jetzt nur so rein fiktiv und das wird wahrscheinlich auch nicht so sein. Aber wenn jetzt irgendwie, keine Ahnung, sagen wir mal, jemand hat einen Todesfall in der Familie und kann da nicht am Projekt mitarbeiten, obwohl am nächsten Tag halt Abgabe ist. Und Pepper sagt dann: Pech gehabt quasi, du musst jetzt aber trotzdem hier mitarbeiten. Obwohl die Person halt eigentlich gar nicht mehr arbeiten kann. Und irgend so was halt. Das ist jetzt nicht so das beste Beispiel, aber halt irgend so was. #00:19:09#

**Interviewer:**

Dass er wenig Rücksicht nimmt auf so, wenn man halt einen schlechten Tag hat. Dann erwartet er immer noch / #00:19:17#

**Interviewpartner:**

Ja, so was. #00:19:20#

**Interviewer:**

Okay. Kannst du Pepper als Führungskraft vertrauen? #00:19:25#

**Interviewpartner:**

Ja, also ich finde, zu meiner Führungskraft ist halt zum Beispiel auch immer so ein bisschen zum Beispiel der Austausch auch mit anderen Führungskräften, dass die an irgendwelchen Meetings teilnehmen, dass die wissen, was so in der ganzen Firma vor sich geht, auch in anderen Bereichen. Und dann halt auch, dass die dadurch die eigene Abteilung und das eigene Arbeiten besser einschätzen können. Und ich finde, / also ich weiß nicht, ob Pepper da auch auf so Meetings gehen würde und ob die anderen Führungskräfte quasi zu ihm hingehen und sagen: „Hier, Pepper, hast du das schon gehört? Da und da passiert jetzt das.“ Also die würden ja nie zu dem hingehen und irgendwelche Geschichten erzählen, die jetzt passiert sind oder halt so informelle Sachen, die aber trotzdem irgendwie eine Rolle spielen auf der Arbeit. Also inside stories quasi. Und da kann man dem ja keinen Vorwurf machen, aber ich glaube, das fehlt manchmal dann, dass er so gewisse Informationen nicht hat, die jetzt eine normale Person schon hat. #00:20:30#

**Interviewer:**

Das ist interessant. Also so diese informellen Informationen, die so an der Kaffeemaschine weitergegeben werden? #00:20:35#

**Interviewpartner:**

Ja, genau. #00:20:36#

**Interviewer:**

Die fehlen dann. #00:20:37#

**Interviewpartner:**

Ja. #00:20:38#

**Interviewer:**

Welche Verhaltensweisen waren dir sympathisch? #00:20:43#

**Interviewpartner:**

Dass er so Gesten gemacht hat; also er hat die Arme immer ausgebreitet. Also das ist mir aufgefallen. Ansonsten, wie gesagt, die Augen sind halt irgendwie noch so ein bisschen starr und künstlich und das Lachen ist jetzt auch noch nicht so natürlich. #00:21:02#

**Interviewer:**

Okay. Ja, das waren dann auch schon die Sachen, die dich gestört haben, oder? Oder gibt es noch was? #00:21:08#

**Interviewpartner:**

Ja, und halt dieses künstliche Aussehen insgesamt und die künstliche Sprache. #00:21:13#

**Interviewer:**

Wie würde es dir gehen, wenn Pepper jetzt dein Chef wäre? #00:21:19#

**Interviewpartner:**

Also da müsste ich mich auf jeden Fall erst mal umstellen, umgewöhnen. Ich würde mich wahrscheinlich auch mal so mit den Kollegen unterhalten, was die so davon denken. Aber letzten Endes, wenn es halt so wäre, dann wäre es so. Ich meine, ich wäre ja dann nicht der Einzige, der mit dem Pepper klarkommen müsste. Von daher, ja, gut. Ich meine, in einer Abteilung von 20Leuten oder so wäre der Pepper immer noch der einzige Roboter. Von daher ist er immer noch in der Minderheit und ich glaube, von daher fände ich es jetzt nicht so schlimm. Aber es wäre auf jeden Fall eine Umstellung. #00:21:55#

**Interviewer:**

Aber es wäre eine Minderheit, die ja immerhin / der er Aufgaben gibt, Anweisungen gibt. #00:22:03#

**Interviewpartner:**

Ja, schon so. Ja, deswegen halt auch die Sache, ob man den halt so wirklich ernstnehmen kann, weil die Aufgaben, die er mir gibt, die hat er ja wahrscheinlich von irgend jemand anderem und irgend jemand muss den ja so programmiert haben oder eingestellt haben, dass er mir Aufgaben gibt. Von daher denke ich mir dann: Ja, dahinter steckt ja eigentlich doch wieder eine normale, reale Person. #00:22:29#

**Interviewer:**

Aber ich meine, ist ja ähnlich bei normalen Führungskräften, die haben ja in der Regel eine Ebene drüber. #00:22:35#

**Interviewpartner:**

Ja, ich frage mich halt, inwiefern der Pepper so wirklich auch strategisch denken kann und zum Beispiel neue Projekte anfangen kann. Oder ob er die einfach nur so quasi so Standard-, Tagesgeschäftsaufgaben geben kann á la: „Hier, ich habe jetzt eine Mail und kannst du die mal beantworten und bearbeiten?“ Aber ob es jetzt halt so wirklich große Sachen sind, weiß ich halt nicht, ob er das so machen kann. #00:23:08#

**Interviewer:**

Also zum Einen so Projekte, größere Projekte aufsetzen kann? #00:23:12#

**Interviewpartner:**

Ja, genau. Klar, er ist schon mir vorgesetzt, aber ich meine, er würde ja zum Beispiel auch gar nicht mitkriegen, wenn ich jetzt zum Beispiel mich in einen Nachbarraum hocken würde, wo er gar nicht ist und ich nichts machen würde. Ich meine, irgendwann am Ende würde es dann halt vielleicht rauskommen, aber ansonsten würde er ja gar nichts mitbekommen. #00:23:41#

**Interviewer:**

Okay. Meinst du nicht, er läuft ab und zu durch und guckt? #00:23:48#

**Interviewpartner:**

Ja, schon. Aber ich weiß nicht, wie er es dann beurteilen will. Ich meine, ich kann ja da sitzen und auch irgendwas anderes machen einfach vor meinem PC. #00:23:56#

**Interviewer:**

Und vorhin hast du ja gemeint, er könnte wahrscheinlich so Routineaufgaben gut abarbeiten, aber irgendwie so innovative, neue Ideen vorzuschlagen und solche Sachen dann eher nicht? #00:24:13#

**Interviewpartner:**

Ja, ich glaube, innovative Ideen könnte er vielleicht sogar schon vorschlagen ,weil er halt irgendwie durch den PC vielleicht Sachen verknüpfen kann, die man als Mensch jetzt nicht so unbedingt verknüpft. Aber halt, ja, ob er so mega strategisch denken kann? Also ich vergleiche es jetzt halt immer mit irgendwelchen Sachen, die ich auf der Arbeit selber mal gemacht habe und ich frage mich jetzt halt, ob er so Projekte selber initiieren würde oder ob das halt einfach dann gar nicht gestartet werden würde. #00:24:51#

**Interviewer:**

Also so Projekte starten, solche Sachen? #00:24:55#

**Interviewpartner:**

Mhm (zustimmend). #00:24:55#

**Interviewer:**

Wenn er sehr strenges Feedback dir gibt, wie würdest du damit umgehen? #00:25:03#

**Interviewpartner:**

Ja, schwierig. Ich weiß ja gar nicht, inwiefern man mit dem diskutieren kann. Und dann würde ich mir halt / also heißt streng jetzt, dass es auch ungerechtfertigt ist? #00:25:16#

**Interviewer:**

Nein, aber halt sehr genau und eigentlich alles, was für ihn zumindest falsch ist, dir dann auch sagt und halt streng ist. #00:25:29#

**Interviewpartner:**

Ja, ich glaube, ich würde es mir nicht so zu Herzen nehmen, weil ich irgendwie immer noch denken würde: Ja gut, ist halt ein Roboter und er ist vielleicht auch so programmiert, dass er einfach so ein bisschen streng programmiert ist. Dass er halt nur wirklich dann auf den Output am Ende guckt und wenn der halt nicht so ganz stimmt, dass er dann halt streng ist. Ja, ich meine, man kann ja selber seine Leistung auch schon so ein bisschen einschätzen. Von daher, wenn man dann merkt: Ich bin eigentlich schon zufrieden mit meiner Leistung und das von Pepper ist jetzt vollkommen ungerechtfertigt. Ja gut, dann ist es halt so. Das einzig Blöde ist halt dann, wenn quasi die Bewertung von Pepper auch sich auf das Gehalt dann auswirkt. Das ist halt dann schon ärgerlich. Ja, dann denkt man sich wieder: Wie wäre das jetzt halt, wenn ich einen richtigen Vorgesetzten hätte, würde er mich halt anders beurteilen? #00:26:25#

**Interviewer:**

Okay. Also siehst du schon ein bisschen die Gefahr, dass es ein bisschen Unmut bei dir schaffen würde? #00:26:33#

**Interviewpartner:**

Ja, ich glaube schon. Also ich denke, ich würde mir dann schon überlegen. Wie wäre das jetzt, wenn da halt jemand Richtiges sitzen würde? #00:26:39#

**Interviewer:**

Wie gut meinst du, kann Pepper für die Zukunft seines Teams Pläne erstellen und Ziele setzen? #00:26:48#

**Interviewpartner:**

Also ich glaube, kurzfristig könnte ich mir vorstellen, schon ganz gut, dass er da so ein paar Tools an der Hand hat, so ein paar Konzepte, wie man jetzt bestimmte Sachen angehen kann und dass er das dann halt auch plant. Langfristig: Also ich glaube, es kommt halt immer drauf an, wie der auch immer die Informationen bekommt. Weil er muss ja eigentlich immer irgendwoher Informationen bekommen, weil als Mensch oder als Mitarbeiter im Unternehmen bekommst du ja auch immer Informationen irgendwoher; wenn du das Intranet liest oder halt Zeitung liest oder auch wenn du mit Leuten sprichst, bekommst du auch immer was mit. Das heißt, er muss ja eigentlich permanent auch mit Informationen gefüttert werden, damit er halt auch sich so langfristig Gedanken machen kann. Und ich finde, davon hängt es halt echt ab. wenn er immer diese Informationen bekommt und halt so, wie ein richtiger Mitarbeiter über alles im Unternehmen Bescheid weiß, dann ja, denke ich, kann er schon auch langfristig Pläne machen. Aber es ist dann halt auch immer, wie gesagt, diese Sache mit den Informationen, die man so unter der Hand bekommt. Und manchmal sind die auch wichtig und die hat er dann nicht. Von daher finde ich es schwierig, dann so eine langfristige Planung wirklich nur aufgrund von Pepper zu machen. #00:28:32#

**Interviewer:**

Du hast ja jetzt ein paar Mal gemeint, dass Informationen halt wichtig sind, dass du Informationen kriegst. Meinst du nicht, dass er dadurch, dass er eine Maschine ist, mehr Informationen als Input nehmen kann und auch verarbeiten kann? #00:28:48#

**Interviewpartner:**

Glaube ich schon, ja. Aber viele Informationen heißt ja nicht immer / also Quantität ist ja nicht immer Qualität. Von daher, wenn es jetzt halt viele Informationen sind, die aber nicht so viel weiterhelfen, dann bringt es halt nichts. Und manchmal ist ja so eine Information wichtiger als zehn Informationen. Von daher, wenn ihm dann das Entscheidende fehlt, dann ist es halt schwierig. #00:29:16#

**Interviewer:**

Hast du eine Idee, was für Informationen ihm fehlen könnten? Also wir haben ja einmal diese informellen Informationen gemeint. Hättest du da noch was? #00:29:25#

**Interviewpartner:**

(überlegt länger). Ich denke, hauptsächlich halt schon diese informellen Informationen, die jetzt einfach noch nicht im Unternehmen sich herumgesprochen haben oder halt noch nicht / #00:29:42#

**Interviewer:**

Nicht dokumentiert / #00:29:45#

**Interviewpartner:**

Dokumentiert, öffentlich irgendwie gemacht worden sind und die halt so ein bisschen auch Gerüchte sind und dann aber meistens sich doch als wahr erweisen. Ja, so was vielleicht. #00:29:57#

**Interviewer:**

Auch so Anekdoten wahrscheinlich, die (unv. #00:30:00#)

**Interviewpartner:**

Ja. #00:30:01#

**Interviewer:**

Was würdest du vermuten, wie erfolgreich wäre ein Team, das von Pepper geführt wird? #00:30:08#

**Interviewpartner:**

Gute Frage. Also ich glaube, gerade halt jetzt am Anfang nicht so erfolgreich wie ein Team, das von einem Mensch geführt worden wäre. Aber ich weiß halt auch nicht genau, wie gut er jetzt schon programmiert ist. Also ich kann mir halt jetzt vorstellen, dass es am Anfang noch nicht so gut programmiert ist und er sich auch nicht auf alle Situationen einstellen kann. Aber dass er dann halt wahrscheinlich mit der Zeit relativ schnell lernt. Ich denke, der hat eine ganz gute Lernkurve und dann wird es wahrscheinlich das Ergebnis auch verbessern. Aber die Sache ist ja auch immer die: Wie autonom denkt jetzt die Abteilung, unabhängig vom Chef? Also ich meine, da sitzen ja immer noch Leute, die ihren eigenen Verstand haben und die dann auch irgendwelche Sachen von Pepper in Frage stellen können. Und dann, wenn es überhaupt keinen Sinn macht, kann man ja immer noch eine andere Entscheidung treffen. Ich meine, dann ist halt gegen den Chef. Aber es macht ja jetzt keinen Sinn, wenn Pepper sagt: „Baut jetzt da eine Anlage für 20 Millionen Euro“ und man kann aber von vorneherein sehen, dass es null Sinn macht. Dann kann ja die Abteilung auch schon ihren Verstand nutzen. Also ich glaube, der Pepper ist zwar der Chef, aber er ist nicht so, dass er komplett über das ganze Wohl von der Abteilung entscheidet. Von daher. #00:31:48#

**Interviewer:**

Also zum Einen, dass der Pepper zwar der Chef ist, aber nicht volle Entscheidungsmacht hat. Also dass das Team schon ein bisschen mitredet. #00:31:59#

**Interviewpartner:**

Mhm (zustimmend). #00:31:59#

**Interviewer:**

Und das Andere war ja, dass du meinst, am Anfang wackelt es noch ein bisschen, aber halt mit der Zeit kann er relativ schnell viel lernen und in der Zukunft könnte es schon ziemlich erfolgreich werden. #00:32:15#

**Interviewpartner:**

Ja, und die Leute, die er halt unter sich hat, sind ja keine Marionetten. Also die können ja schon selber denken. Von daher glaube ich, ist das jetzt nicht so abhängig von Pepper. m#00:32:15#

**Interviewer:**

Okay. Sehr gut.
