# Supplementary material for: Robot leadership–Investigating human perceptions and reactions towards social robots showing leadership behaviors
Source: PLoS One. 2023 Feb 16;18(2):e0281786. doi: 10.1371/journal.pone.0281786 (PMC9934409; doi:10.1371/journal.pone.0281786)
Supplement: S1 Dataset — (ZIP) [file pone.0281786.s002.zip › Interview_Participant3.doc]

**16 Interview**

**Interviewer:**

So als Prämisse, stell dir vor, du arbeitest jetzt in einem Team mit Pepper als Führungskraft. Welche Gedanken sind dir durch den Kopf gegangen, während Pepper mit dir geredet hat? #00:00:16#

**Interviewpartner:**

Als Erstes ist mir aufgefallen, dass die Stimme noch relativ unmenschlich klingt. Das hat mich ein bisschen gestört, muss ich sagen. Von der Gestik fand ich es relativ gut. Es sah relativ human aus. Sonst? Ich habe auch allgemein Probleme gehabt, den ein bisschen zu verstehen von der Deutlichkeit. So vom äußeren Erscheinungsbild wirkt er relativ, ja, freundlich. Ja. #00:00:57#

**Interviewer:**

Wie hat es sich für dich angefühlt, von Pepper angesprochen zu werden? #00:01:02#

**Interviewpartner:**

Es war relativ normal. Wie gesagt, man hat sofort gemerkt, es ist halt ein Computer oder eine Computerstimme und somit, dass man einfach wie von einem PC angesprochen wird. #00:01:23#

**Interviewer:**

Okay. Und wie fandest du das? #00:01:25#

**Interviewpartner:**

Ja, also wenn man es jetzt so betrachtet als Führungskraft, würde ich sagen, eher ein bisschen schwierig, weil da so noch keine Vertrauensbasis sozusagen da ist. Aber das wäre auch zum Beispiel, wenn das jetzt eine normale Person gewesen wäre, wäre es genauso, weil da noch nichts aufgebaut ist. #00:01:46#

**Interviewer:**

Okay. Dadurch, dass du davor keine Erfahrung mit ihm gemacht hast? #00:01:49#

**Interviewpartner:**

Ja, genau. #00:01:50#

**Interviewer:**

Welche Gedanken hast du dir gemacht, als Pepper gesagt hat: „My vision ist hat we as them marketing department can make people passional about our product.“? #00:01:59#

**Interviewpartner:**

Ja, man hat sozusagen versucht, dass er das mit Überzeugung und auch Enthusiasmus rüberbringt und mit den Gesten. Das fand ich sogar einigermaßen gut gemacht. #00:02:17#

**Interviewer:**

Okay. Also wie er es rübergebracht hat? #00:02:20#

**Interviewpartner:**

Genau. #00:02:20#

**Interviewer:**

Das (war) gut? #00:02:22#

**Interviewpartner:**

Ja. #00:02:23#

**Interviewer:**

Und wie gut hat Pepper seine Vision da für die Zukunft definiert? #00:02:30#

**Interviewpartner:**

Jetzt müsste ich mich dran erinnern. (überlegt) Relativ gut. Wie ist genau die Frage gemeint? Was er genau gesagt hat oder / #00:02:48#

**Interviewer:**

Also wie er es gesagt hat und was er gesagt hat: War es für dich eine stimmige Vision? War es für dich / #00:02:58#

**Interviewpartner:**

Ja, relativ, müsste ich sagen. Also wie er es rübergebracht hat, fand ich ganz gut. Vom Inhalt her, da fand ich immer, wie ich es schon gesagt habe, die Sprache war das ein bisschen ein Hindernis, weil es immer noch sehr roboterhaft klang und so gehackt, finde ich; keine flüssige Sprache war. #00:03:19#

**Interviewer:**

Und hast du ihm geglaubt, dass es seine Vision ist oder eher nicht? #00:03:24#

**Interviewpartner:**

Eher ja. #00:03:27#

**Interviewer:**

Pepper hat auch gesagt: „As a leader I always want to be a role model.“ Was hältst du davon? Inwiefern meinst du, dass Pepper ein Vorbild für seine Mitarbeiter sein kann? #00:03:37#

**Interviewpartner:**

Da ist dann halt die Frage, wie weit ein Roboter ein Bewusstsein hat und man muss ja immer im Hintergrund haben, dass es eigentlich ja nur eine Programmierung ist und dementsprechend zu glauben, dass er das wirklich meint, ist halt schwierig, dann zu sagen: Ja, er steht da mit voller Überzeugung dahinter. Weil es ist halt nur ein Programm und da zu sagen: Ja, der meint das mit voller / aus innerem Herzen wie ein Mensch, geht halt nicht. Und dass er eigentlich einem nur was vorspielen / #00:04:17#

**Interviewer:**

Meinst du aber, er könnte irgendwie ein Vorbild für Mitarbeiter sein? #00:04:22#

**Interviewpartner:**

Das auf jeden Fall. Wenn der noch ein bisschen überzeugender wird, was Gestik und die Sprache betrifft, dann könnte es auf jeden Fall zumindest in der Zukunft überzeugender werden. #00:04:34#

**Interviewer:**

Okay. Meinst du, er kann so ganzheitlich als Vorbild sein oder nur in irgendwelchen Bereichen? #00:04:42#

**Interviewpartner:**

Also ich glaube eher, dass er, wenn es jetzt darum geht / wenn es um spezifische Fragen, die ein Computer besser beantworten kann wie ein Mensch, dass ich vielleicht da dem Roboter eher vertrauen würde wie einem Menschen. Aber wenn es um emotionale Sachen geht, also wenn ich mich zum Beispiel irgendwo beschweren möchte, dass es da dann eher schwieriger wird beim Roboter. #00:05:09#

**Interviewer:**

Okay. Wieso? #00:05:09#

**Interviewpartner:**

Weil momentan sehe noch nicht so wirklich, ob er mich dann auch verstehen könnte. #00:05:17#

**Interviewer:**

Inhaltlich, was du sagst oder irgendwie dich / #00:05:24#

**Interviewpartner:**

Ja, eher inhaltlich. #00:05:26#

**Interviewer:**

Also ob er auch versteht, was du sagst? #00:05:27#

**Interviewpartner:**

Genau. #00:05:30#

**Interviewer:**

Wenn ich dir jetzt sage, er versteht, das du sagst? #00:05:35#

**Interviewpartner:**

Dann ist halt die Frage: Auch wenn er es versteht, wie kann er es bearbeiten? Also wenn er wirklich so weit wäre, dass er auf mich vollkommen rational reagieren könnte, dann sähe ich es vielleicht sogar als gute Alternative zu einem menschlichen Chef. #00:06:01#

**Interviewer:**

Also wenn er dich versteht und es auch gut bearbeiten kann / #00:06:08#

**Interviewpartner:**

Verarbeiten kann, ja. #00:06:08#

**Interviewer:**

Dann wäre es gut? Okay. Wenn du jetzt in einem Team wärst mit Pepper als Führungskraft, würdet ihr gemeinsame Ziele anstreben, die von Pepper gesetzt werden? #00:06:22#

**Interviewpartner:**

Na ja, wenn er meine Führungskraft ist und er sozusagen das vorgibt, dann wäre es auf jeden Fall logisch, wenn man dann auch als Team zusammenarbeitet, also mit den anderen Mitgliedern. Das schon. #00:06:39#

**Interviewer:**

Also ja? #00:06:42#

**Interviewpartner:**

Ja. #00:06:42#

**Interviewer:**

Also würdet ihr das auch so akzeptieren? #00:06:44#

**Interviewpartner:**

Ja. #00:06:45#

**Interviewer:**

Okay. Inwiefern hast du das Gefühl, dass Pepper hohe Erwartungen an euch als Team hätte? #00:06:51#

**Interviewpartner:**

Nachdem er das schon mit so einer gewissen Leidenschaft rübergebracht hat und es kam auch so eine gewisse Erwartungshaltung rüber, dass dann auch was von einem selber kommen sollte und da fand ich, das war schon relativ gut gemacht. Aber auch, ja, in gewissem Maße auch leicht fordernd das rübergebracht. #00:07:16#

**Interviewer:**

Also hätte er schon relativ hohe Erwartungen? #00:07:19#

**Interviewpartner:**

Ja. #00:07:20#

**Interviewer:**

In welchem Ausmaß, glaubst du, dass Pepper dich als Teammitglied angemessen in deiner Arbeit unterstützen kann? #00:07:32#

**Interviewpartner:**

(überlegt etwas) Also ich kenne ja jetzt seine Fähigkeiten noch nicht so genau, aber wenn es jetzt zum Beispiel um Datenaufbereitung oder solche Sachen geht, könnte ich mir das dann gut vorstellen. Ob ich mir jetzt so eine direkte Zusammenarbeit mit Menschen vorstellen könnte, weiß ich noch nicht. Also da ist dann auch immer die Frage, wie gut er einen verstehen kann. Aber wenn er auf einem, ich sag mal, menschlichen Niveau ist, dann wäre das für mich kein Problem, mit ihm auch genauer zusammenzuarbeiten. #00:08:10#

**Interviewer:**

Also wenn er einen Dialog halten kann und dann könnte er dich auch unterstützen in deiner Arbeit? #00:08:17#

**Interviewpartner:**

Ja, das auf jeden Fall. #00:08:19#

**Interviewer:**

Okay. Würdest du nicht nur auf Daten aufbereiten und solche Sachen beschränken? #00:08:26#

**Interviewpartner:**

Ja, das kommt halt auf die Qualität von ihm an. Wenn er wirklich auch gute, eigene Ideen hat, dann würde ich die auf jeden Fall auch annehmen. #00:08:38#

**Interviewer:**

Inwiefern hast du das Gefühl, dass sich Pepper auf dich und Andere anpasst? #00:08:44#

**Interviewpartner:**

So zumindest kam es mir jetzt nicht so vor, als hätte er mich speziell beobachtet oder speziell seine Mimik und Gestik angepasst. Also für mich kam es eher so vor, als wäre das so eine vorprogrammierte Sequenz gewesen, die immer gleich ist. Also spezielle Anpassung habe ich jetzt für mich nicht erfahren. #00:09:09#

**Interviewer:**

Und wenn du dir jetzt vorstellst, du würdest jetzt für ihn arbeiten: Meinst du, er würde sich irgendwie anpassen, zum Beispiel auch Rücksicht auf persönliche Bedürfnisse nehmen oder solche Sachen? #00:09:23#

**Interviewpartner:**

Eher weniger. Also mir kam es zumindest jetzt in dem kurzen Gespräch nicht so vor, als würde er auf individuelle Bedürfnisse eingehen. Aber das ist jetzt nach dem kurzen auch ein bisschen schwer zu sagen. #00:09:36#

**Interviewer:**

Meinst du, er könnte sich anpassen, zum Beispiel wenn jemand oder du einen schlechten Tag hast: Würde er das merken und dann sich anpassen oder eher nicht? #00:09:52#

**Interviewpartner:**

Das ist halt dann immer eine Frage, inwiefern er eine Wahrnehmung hat von einer Person. Es ist bestimmt technisch möglich, dass er erkennt, dass jemand nicht so einen guten Tag hat oder auch, wenn man ihm das einfach sagt. Das ist halt dann die Frage, inwieweit er drauf eingeht. Eher weniger, würde ich sagen. #00:10:16#

**Interviewer:**

Also würdest du sagen, er nimmt da wenig Rücksicht? #00:10:20#

**Interviewpartner:**

Ja. #00:10:20#

**Interviewer:**

Und macht sein Ding? #00:10:21#

**Interviewpartner:**

Ja. #00:10:23#

**Interviewer:**

Inwiefern hat dich Pepper angeregt, neue und innovative Ideen zu produzieren? #00:10:29#

**Interviewpartner:**

Also dadurch, dass er es schon mit einer gewissen, ja, Emotion, aber auch Leidenschaft rübergebracht hat, würde ich schon sagen, dass er es auf einem guten Niveau gemacht hat und dadurch auch mich schon angeregt hat, da gute Ideen zu bringen. #00:10:53#

**Interviewer:**

Denkst du, Pepper würde dich ermutigen, den Status quo anzuzweifeln? #00:10:59#

**Interviewpartner:**

Was ist der Status quo? #00:11:01#

**Interviewer:**

Zum Beispiel bei der Arbeit so Arbeitsabläufe oder Prozesse, halt wie man arbeitet. #00:11:09#

**Interviewpartner:**

Ja, also das auf jeden Fall. Wenn da zum Beispiel Vorschläge kämen, wie man was verbessern kann und das von ihm (unv. #00:11:17#) und die sinnvoll wären, auf jeden Fall. #00:11:21#

**Interviewer:**

Und er könnte dich dann auch anregen, selbst darüber nachzudenken? #00:11:30#

**Interviewpartner:**

Ja. #00:11:31#

**Interviewer:**

Würdest du für Pepper beziehungsweise für die Ziele oder das Ziel von Peppers Abteilung mehr als das zwingend Notwendige tun? #00:11:47#

**Interviewpartner:**

(überlegt kurz). Also es ist halt so eine persönliche Einstellung, wie man allgemein in der Arbeit ist. Aber ich würde sagen, das ist jetzt genauso wie bei einem menschlichen Chef, dass ich auch schon ein bisschen mehr geben würde als nur das Nötigste. #00:12:08#

**Interviewer:**

Aber du meinst, das liegt dann eher / #00:12:10#

**Interviewpartner:**

An der persönlichen Einstellung, ob das jetzt ein Roboter oder ein Mensch ist. #00:12:14#

**Interviewer:**

Wie war dein Gesamteindruck von Pepper? #00:12:22#

**Interviewpartner:**

Gesamteindruck? Dass es schon relativ gut gemacht ist. Wie ich schon angesprochen habe, dass man dieser Stimme noch ein bisschen arbeiten müsste, dass die ein bisschen menschlicher ist. Gestik und, ja: Mimik hat er ja nicht. Aber das wäre vielleicht auch noch ein Schritt, dass da auch noch ein bisschen Mimik eingebaut wird. Aber von der Gestik schon relativ gut, jedoch noch ein bisschen so abgehackt; also keine flüssigen Bewegungen, sondern immer noch ein bisschen stückhaft. Aber so im Gesamteindruck ein annehmbarer Chef (lacht). #00:12:59#

**Interviewer:**

Inwiefern hast du dich von Pepper motiviert gefühlt? #00:13:05#

**Interviewpartner:**

Ja, auch auf einem guten Niveau. #00:13:11#

**Interviewer:**

Okay. Also vielleicht auch , wenn du dir vorstellst, du würdest für ihn arbeiten: Inwiefern würde er dich motivieren? #00:13:17#

**Interviewpartner:**

Ja, also ich würde jetzt nicht in die Lüfte springen vor Freude, aber ja, also er motiviert. Aber jetzt auf keinem überdurchschnittlichen Niveau. #00:13:32#

**Interviewer:**

Vielleicht wenn du es mit einem Menschen vergleichen würdest? #00:13:37#

**Interviewpartner:**

Tendenziell eher / Also kommt natürlich auf den Menschen an, weil da jeder unter / Aber tendenziell eher ein bisschen unter einem Menschen. #00:13:44#

**Interviewer:**

Und weißt du, wieso? #00:13:46#

**Interviewpartner:**

Weil ich finde, ein Mensch kann doch noch auf einen persönlich mehr eingehen wie jetzt der Roboter, der jetzt eigentlich nur die Sequenz runterspielt. #00:13:58#

**Interviewer:**

Okay, also meinst du, ein Roboter oder Pepper jetzt wäre nicht so in der Lage, auf dich einzugehen und zu gucken, was für dich eher funktioniert? #00:14:09#

**Interviewpartner:**

Da wäre ein Mensch, glaube ich, eher fähig. Also zumindest ein Großteil der Menschen. #00:14:15#

**Interviewer:**

Fühlst du dich von Pepper wertgeschätzt? #00:14:18#

**Interviewpartner:**

(überlegt etwas). Also da doch keine wirkliche Reaktion jetzt von ihm kam, kam jetzt auch keine wirkliche Wertschätzung rüber. Also er hat das so vorgetragen, aber jetzt so individuell auf mich gesehen, habe ich das jetzt nicht gefühlt. #00:14:41#

**Interviewer:**

Und wenn du dir jetzt wieder vorstellst, du würdest für ihn arbeiten: Meinst du, er würde dich wertschätzen? #00:14:46#

**Interviewpartner:**

(überlegt etwas). Eher nein. Mir kam es jetzt nicht so vor, als würde er auf die individuellen Wünsche eingehen. #00:15:02#

**Interviewer:**

Okay. Aber würde er deine Arbeit wertschätzen? #00:15:07#

**Interviewpartner:**

(überlegt etwas) Jein. Also ich denke, er könnte, wenn die Arbeit gut gemacht ist, sagen: „Das passt.“ Aber ich weiß nicht, ob es darüber hinausgehen könnte. Also eher nicht. #00:15:23#

**Interviewer:**

Also meinst du, er würde dich dann auch nicht loben oder / #00:15:32#

**Interviewpartner:**

Eher nicht. #00:15:35#

**Interviewer:**

Und wenn er dich loben würde, würdest du das irgendwie positiv wahrnehmen oder wäre es für dich eher einfach nur so „Ja.“ #00:15:45#

**Interviewpartner:**

Man hat halt immer noch im Hinterkopf: Es ist nur von dem Roboter gesagt. Und deswegen ist es, glaube ich, nicht ganz so wertschätzend wie wenn es jetzt ein Mensch zu einem sagen würde. #00:15:56#

**Interviewer:**

Was meinst du, woran liegt das? #00:15:58#

**Interviewpartner:**

Wenn man da halt immer so im Hinterkopf hat: Ja, ein Roboter sagt halt das, weil es ihm vorprogrammiert ist oder ein Mensch würde es eher sagen von sich aus. Und beim Rob / ja, ist schwierig. Da ist halt die Frage, wie weit er das schaffen könnte, mich zu beurteilen, ob das wirklich eine gute Arbeit ist oder er das nur sagt, weil es so im Programm vorgeschrieben ist. #00:16:34#

**Interviewer:**

Okay. Also meinst du, er würde vielleicht nicht ganz so gut das beurteilen können, ob das eine gute Arbeit oder eine schlechte Arbeit ist? #00:16:45#

**Interviewpartner:**

Ja. #00:16:46#

**Interviewer:**

Denkst du, Pepper ist fair zu seinen Teammitgliedern? #00:16:52#

**Interviewpartner:**

Ich würde fast sagen, dass jetzt Pepper vielleicht das sogar rationaler alles ansieht und somit auch die Arbeit rationaler und fairer somit aufteilt, als ein Mensch. Also von dem her gesehen wäre er da vielleicht sogar im Vorteil. #00:17:11#

**Interviewer:**

Also dadurch, dass er das rational dann macht, ist er fairer? #00:17:17#

**Interviewpartner:**

Ja. #00:17:17#

**Interviewer:**

Ich weiß nicht, wenn du dir überlegst: Es gibt ja Menschen, die dann vielleicht mehr Zuwendung brauchen, Menschen, die weniger Zuwendung brauchen. Da ist da natürlich Definitionssache, was fair ist. Also wenn du vielleicht noch mal das überlegst: Ist er dann fair? #00:17:44#

**Interviewpartner:**

Ja, also (überlegt kurz) er könnte halt dann wenig auf die individuellen Bedürfnisse eingehen, aber trotzdem würde ich es fast fairer finden, wenn es rationaler verteilt ist. #00:17:57#

**Interviewer:**

Also objektiv fairer wäre es dann? #00:18:00#

**Interviewpartner:**

Ja. #00:18:00#

**Interviewer:**

Kannst du Pepper als Führungskraft vertrauen? #00:18:03#

**Interviewpartner:**

Also in der kurzen Zeit konnte ich jetzt keine wirkliche Vertrauensbasis jetzt sozusagen aufbauen. Aber wenn ich jetzt ihn noch näher kennenlernen würde, könnte ich es mir gut vorstellen, dass da schon ein gewisses Vertrauen da ist. #00:18:21#

**Interviewer:**

Inwiefern würdest du ihm da vertrauen? #00:18:24#

**Interviewpartner:**

Da jetzt auch wieder, wie schon gesagt, eher in rationalen Sachen, wo alles eher datenbasiert ist, würde ich ihm eher vertrauen vielleicht wie einem Menschen. Wenn es jetzt aber um, sag ich jetzt mal, Bauchentscheidungen geht, würde er sich wahrscheinlich eher schwerer tun. (lacht) #00:18:44#

**Interviewer:**

Okay. Also würdest du ihm vertrauen, dass er die richtigen Daten gibt, dass er die richtig bearbeitet? #00:18:51#

**Interviewpartner:**

Ja, das auf jeden Fall. #00:18:52#

**Interviewer:**

Aber dann so, weiß nicht, kreativere Sachen oder Bauchgefühlsentscheidungen sind dann schwierig? #00:19:01#

**Interviewpartner:**

Ja. #00:19:02#

**Interviewer:**

Welche Verhaltensweisen waren dir sympathisch? #00:19:05#

**Interviewpartner:**

Sympathisch? Ja, wie auch schon angesprochen, sein Enthusiasmus, wie er Sachen rüberbringt, war sehr gut. So vom Erscheinungsbild wirkt er jetzt, ich sag mal, nicht einschüchternd, sondern ganz, ja, relativ human und, ja, also vom Erscheinungsbild passt es auch. #00:19:33#

**Interviewer:**

Und was hat dich an seinem Verhalten gestört? #00:19:38#

**Interviewpartner:**

Da komme ich noch mal auf die Sprache zurück. Dann auch, dass er halt keine oder nur geringe Mimik hat. Das finde ich dann ein bisschen schwierig: Die Sprache kommt raus, aber es kommt halt irgendwo aus dem Lautsprecher raus und es wird nicht so wirklich klar, dass es von ihm wirklich ist. Dieser Bildschirm vorne drauf macht es jetzt auch nicht viel besser. #00:20:12#

**Interviewer:**

Okay. Wieso? #00:20:12#

**Interviewpartner:**

Es macht ihn halt eher noch mehr zum Roboter, wenn das jetzt so ein Teil vorne draufklebt. Und dann seine komischen Zuckungen. Also wenn er jetzt so im Raum steht und dann immer wieder so zuckt, das macht ihn irgendwie nicht menschlicher. #00:20:33#

**Interviewer:**

Wenn er ab und zu so (unv. #00:20:35#)

**Interviewpartner:**

Ja. Es ist wahrscheinlich beabsichtigt, dass er jetzt so diese Finger bewegt und so, aber ich finde, das macht ihn irgendwie / es bewirkt eher das Gegenteil, immer mal wieder so sich bewegt. Also wenn er ruhig dort stehen würde, wäre es mir fast lieber (lacht). #00:20:51#

**Interviewer:**

Wie würde es dir gehen, wenn Pepper jetzt dein Chef wäre? #00:20:55#

**Interviewpartner:**

Also beim momentanen Stand der Technik wäre mir immer noch ein Mensch lieber, muss ich ehrlich sagen. Aber ich sage mal, wenn es nicht anders geht, würde ich es akzeptieren oder mir bliebe wahrscheinlich nichts anderes übrig, als es zu akzeptieren. Aber ein Mensch wäre mir trotzdem lieber. #00:21:17#

**Interviewer:**

Also momentan wäre dir ein Mensch lieber, aber du würdest auch einen Roboter / #00:21:22#

**Interviewpartner:**

Ja, würde ich auch akzeptieren, wenn es mir vorgeschrieben werden würde. #00:21:27#

**Interviewer:**

Und in der Zukunft? #00:21:30#

**Interviewpartner:**

Also wenn er noch weitere Fortschritte macht, vor allem dann auch im Analysieren von meinen Sachen, was ich ihm an Informationen gebe und wie er die auswertet, dann würde ich es auf jeden Fall zumindest als eine gute Ergänzung sehen zu einem menschlichen Chef. Also ob ich ihn jetzt vollwertig als Ersatz sehen würde, sehe ich auch noch kritisch. Aber so als Ergänzung zu einem menschlichen Chef auf jeden Fall. Aber das ist halt dann schwierig, inwieweit dann die Aufteilung ist. #00:22:08#

**Interviewer:**

Okay. Also wäre es dir gewissermaßen lieber, wenn er dann so / #00:22:14#

**Interviewpartner:**

Eher unterstützende Maßnahme wie jetzt als leitender Chef. #00:22:18#

**Interviewer:**

Okay. Als Assistent zum Chef praktisch? #00:22:21#

**Interviewpartner:**

Genau. #00:22:22#

**Interviewer:**

Wenn er jetzt dein Chef wäre, was würde gut oder was nicht so gut funktionieren? #00:22:27#

**Interviewpartner:**

Also allein die Interaktion würde ich schwierig halt finden, mit einem Roboter jetzt zu reden oder auch meine Argumente rüberzubringen. Da würde ich immer es schwierig finden, ob er überhaupt versteht, was ich meine. Aber wenn er mir jetzt Anweisungen gibt, hätte ich wahrscheinlich eher weniger Probleme damit. #00:22:57#

**Interviewer:**

Also meinst du, bei der Interaktion oder Dialog, da gäbe es ein paar Schwierigkeiten? #00:23:06#

**Interviewpartner:**

Ja. Also das wird halt wahrscheinlich eine Zeit brauchen, bis man da rausfindet, was er versteht, was nicht. Das wird wahrscheinlich einfach eine gewisse Zeit brauchen, bis man sich auch drauf einlässt. #00:23:20#

**Interviewer:**

Aber mit der Zeit, meinst du / #00:23:22#

**Interviewpartner:**

Mit Zeit und wenn man merkt, es funktioniert gut, dann könnte ich es mir schon gut vorstellen. #00:23:27#

**Interviewer:**

Wenn er sehr strenges Feedback gibt, wie würdest du damit umgehen? #00:23:34#

**Interviewpartner:**

Das wäre mir fast lieber wie wenn jetzt ein Mensch so um den heißen Brei rumredet. Da wüsste ich genau, wo es hakt und wo nicht und dementsprechend wäre mir das eigentlich ganz recht. #00:23:49#

**Interviewer:**

Also würdest du es gut finden ,dass er so direkt alles sagt? #00:23:53#

**Interviewpartner:**

Ja. #00:23:53#

**Interviewer:**

Und du würdest dann auch daran arbeiten, was er dir so sagt, was nicht so gut war? #00:23:59#

**Interviewpartner:**

Ja, relativ sicher. #00:24:01#

**Interviewer:**

Wie gut, meinst du, kann Pepper für die Zukunft seines Teams Pläne erstellen oder auch Ziele setzen? #00:24:10#

**Interviewpartner:**

Da sehe ich halt noch ein bisschen so die Gefahr, da das noch fehlt, dass dieses vorausschauende Denken vielleicht noch nicht so vorhanden ist, dass er vielleicht eher nur kurzfristig fixiert ist. Er hat zwar in seinem Vortrag so ein bisschen die Vision rübergebracht, aber ich weiß jetzt nicht, ob das wirklich so ernst gemeint war oder halt nur, ob das die Vorgabe war. #00:24:40#

**Interviewer:**

Also meinst du, er kann momentan noch nicht so weit in die Zukunft sehen? #00:24:45#

**Interviewpartner:**

Ich sehe es halt eher so, dass er halt jetzt so die Aufgaben abwickelt. Aber ob er wirklich schon so Visionen erstellen kann, bezweifle ich. #00:24:55#

**Interviewer:**

Was meinst du, wie es dann in der Zukunft sein könnte? #00:24:58#

**Interviewpartner:**

Da könnte ich es / Ja, es kommt halt auf den Stand der Technik drauf an. Aber ich könnte mir dann schon vorstellen, dass das Bewusstsein dann sich auch entwickelt und somit dann er auch ein strategisches Denken sozusagen entwickelt; auch: Wie ist der aktuelle Stand der Technik und wie müssen wir dann unser Produkt anpassen? Dass er da so eine gewisse Wahrnehmung entwickelt, könnte ich mir gut vorstellen. #00:25:26#

**Interviewer:**

Letzte Frage: Was würdest du vermuten, wie erfolgreich wäre ein Team, das von Pepper geführt wird? #00:25:34#

**Interviewpartner:**

Also aktuellen Stand, so wie er jetzt ist, würde ich jetzt mal behaupten, dass es eher weniger performed wie jetzt ein Team mit einem Menschen. Weil ich glaube, die Interaktion noch nicht so vollkommen ausgearbeitet ist. Aber wenn er sich noch weiterentwickelt, dann könnte ich mir schon vorstellen, dass irgendwann mal das auch eine Führungskraft ersetzen kann, die menschlich ist und somit auch zu einer besseren Performance führen könnte. #00:26:10#

**Interviewer:**

Also kurzfristig eher schlechter als ein Mensch, aber langfristig dann schon besser? #00:26:17#

**Interviewpartner:**

Ja.

**Interviewer:**

Sehr gut.
