# Supplementary material for: Robot leadership–Investigating human perceptions and reactions towards social robots showing leadership behaviors
Source: PLoS One. 2023 Feb 16;18(2):e0281786. doi: 10.1371/journal.pone.0281786 (PMC9934409; doi:10.1371/journal.pone.0281786)
Supplement: S1 Dataset — (ZIP) [file pone.0281786.s002.zip › Interview_Participant4.doc]

**14 Interview**

**Interviewer:**

So als Prämisse: Stell dir vor, du arbeitest jetzt in einem Team, das von Pepper geführt wird. Welche Gedanken sind dir durch den Kopf gegangen, während Pepper mit dir geredet hat? #00:00:19#

**Interviewpartnerin:**

Die Stimme ist sehr unnatürlich, ist eine komische Tonlage von der Stimme. Ich habe mich gefragt, wo die Stimme herkommt. Ob die aus dem Mund sozusagen dann kommt oder irgendwie so aus den Ohren. Ich weiß nicht, ich habe am Anfang dem gar nicht in die Augen geschaut. Ich habe auf den Bildschirm geschaut, auch wenn nichts passiert ist. Am Ende ist mir dann aufgefallen, dass ich ihm nicht in die Augen schaue, dann habe ich ihm in die Augen geschaut, habe so getan, als wäre es sozusagen ein Mensch. Aber ich finde es nach wie vor ein bisschen unheimlich (lacht) irgendwie. (unv. #00:00:53#) nicht angesprochen gefühlt (lacht). #00:00:56#

**Interviewer:**

Wie hat es sich für dich angefühlt, von Pepper angesprochen zu werden? #00:01:03#

**Interviewpartnerin:**

Also nachdem / Also wenn die Stimme halt menschlicher gewesen wäre, dann hätte es mich wahrscheinlich eher irritiert als wenn halt so im Gesamtkonzept halt man gemerkt hat, dass es ein Roboter ist sozusagen. Dass man halt nicht versucht hat, so wirklich ganz den Menschen zu 100 % nachzumachen. Es gibt ja zum Beispiel auch so Puppen, die haben dann auch so eine Haut, die dann so menschlich aussieht. Insofern war das eigentlich voll okay eigentlich. #00:01:37#

**Interviewer:**

Also fandest du es gut, dass es nicht zu menschlich aussieht und sich auch nicht zu menschlich anhört? #00:01:41#

**Interviewpartnerin:**

Es muss halt passen. Wenn du weißt, du redest mit einem Roboter / Nein, wenn du / Wie ich soll denn das sagen? Zum Beispiel, wenn du einen Chatbot hast auf einer Webseite, dann sollte irgendwo dastehen, dass du mit einem Chatbot redest, weil wenn du sagst hinterher, es ist ein Mensch und er macht irgendwelche komische Fehler oder so, dann ist es irgendwie irritierend. Und so meine ich bei dem auch irgendwie. Wenn es halt nicht zusammenpasst, so die Eigenschaften, die er hat, zum Beispiel wenn die Stimme halt so menschlich wäre, dann wäre es halt irgendwie irritierend oder – ich weiß nicht. #00:02:17#

**Interviewer:**

Also findest du es gut, dass man klar erkennt, es ist ein Roboter? #00:02:20#

**Interviewpartnerin:**

Ja. Wenn seine Funktion ein Roboter sein soll, dann ja. #00:02:26#

**Interviewer:**

Und wenn es so menschenähnlich sein sollte, würdest du es dir dann eher wünschen, dass es auch wie ein Mensch aussieht? #00:02:38#

**Interviewpartnerin:**

Also ich weiß nicht genau, was seine Funktion ist. Du hast ja gesagt, das ist irgendwie so Leadership, oder er hat es ja auch gesagt. Ich weiß nicht, wenn der jetzt irgendwie so einen Vortrag halten würde oder so was, dann fände ich es komisch. Noch während er geredet hatte, dachte ich mir so: Okay, irgend jemand muss ja trotzdem diesen Text da eingespeichert haben, also ist ja doch irgendwo ein Mensch dahinter, also eine reale Person. Was war noch mal die Frage? #00:03:03#

**Interviewer:**

Wie es sich für dich angefühlt hat, dass er mit dir geredet hat oder auch eben, ob / wenn er jetzt als Führungskraft auftritt, sollte er menschlicher aussehen oder ist er so okay? #00:03:20#

**Interviewpartnerin:**

Nein, ich finde das so okay. Er ist ja kein Mensch. #00:03:25#

**Interviewer:**

Wäre sonst unheimlich, wenn es so / #00:03:27#

**Interviewpartnerin:**

Ja. #00:03:29#

**Interviewer:**

Welche Gedanken hast du dir gemacht, als Pepper gesagt hat „My vision ist hat we as them marketing department can make people appassionate about our product.“? #00:03:40#

**Interviewpartnerin:**

Ich weiß nicht. Vielleicht habe ich mir da auch Gedanken darüber gemacht, dass es ja irgend jemand so geschrieben haben muss. Er hat auch am Ende gesagt irgendwie so: „Ich hoffe, dass ihr genauso viel Engagement in dem Projekt zeigt wie ich auch in meinem Job zeige“ oder so. Da dachte ich mir auch sehr: Komm! Als ob / das ist halt kein Mensch so, der Engagement zeigen kann. Er zeigt mir so viel Engagement, wie man es ihm beigebracht hat sozusagen. Deswegen kann ich das, glaube ich, nicht sehr ernstnehmen, weil er halt einfach / es ist halt kein Mensch, der Erfahrung hat und der auch dann / der einen quasi die / also ich weiß nicht. Ich habe nicht das Gefühl, dass ich mit einem Menschen geredet hatte. Aber ich kann das halt nicht so ganz ernst nehmen. #00:04:38#

**Interviewer:**

Okay, aber ich meine / #00:04:40#

**Interviewpartnerin:**

Also jedes Mal, wenn er irgendwas gesagt hat, was so menschlich ist sozusagen, wie dieses mit dem Marketingdepartment oder was du da gerade gesagt hast, dann habe ich (unv. #00:04:50#) so gedacht: Mhm, genau. Weil ich das halt nicht ernst nehmen konnte. #00:04:55#

**Interviewer:**

Nicht ernst genommen wieso? Weil es ein Roboter ist? #00:04:59#

**Interviewpartnerin:**

Mhm (zustimmend). #00:05:00#

**Interviewer:**

Und das traust du ihm nicht zu oder / #00:05:03#

**Interviewpartnerin:**

Mhm (zustimmend). #00:05:05#

**Interviewer:**

Okay. Wie gut hat Pepper seine Vision für die Zukunft definiert und auch jetzt präsentiert? #00:05:14#

**Interviewpartnerin:**

Ich fand so die Gestik gut, also sehr realistisch irgendwie, wie halt jemand, der gut präsentieren kann, es vielleicht präsentieren würde. Ja, also Mimik gibt es ja nicht wirklich, da ist halt ein bisschen der Nachteil, weil Mimik spielt ja auch irgendwie eine wichtige Rolle. Aber mit der Gestik hat er das ein bisschen wettgemacht sozusagen. #00:05:49#

**Interviewer:**

Also hat er es gut präsentiert? #00:05:50#

**Interviewpartnerin:**

Er hat es eigentlich ganz gut präsentiert. #00:05:52#

**Interviewer:**

Und wie gut, meinst du, hat er die Vision definiert? #00:05:58#

**Interviewpartnerin:**

(überlegt etwas). Ich kann mich ehrlich gesagt nicht mehr so ganz dran erinnern (lacht). #00:06:10#

**Interviewer:**

Okay (lacht). #00:06:11#

**Interviewpartnerin:**

Falls das auch eine Antwort ist. #00:06:12#

**Interviewer:**

Pepper hat auch gesagt: „As a leader I always want to be a role model.“ Was hältst du davon? Inwiefern meinst du, dass Pepper ein Vorbild für seine Mitarbeiter sein kann? #00:06:25#

**Interviewpartnerin:**

Na ja, er ist ja ein Roboter. Sein einziges Ding ist ja, zu optimieren, oder? Also er bekommt ja verschiedene Einflüsse oder ich weiß nicht genau, wie das funktioniert. Aber ich stelle es mir halt so vor, dass er halt eben verschiedenen Einflüssen ausgesetzt wird oder Situationen und die analysiert und dann halt immer den besten Weg sucht oder so was. Und wenn man das oft genug macht, dann trifft er auf jeden Fall mit größerer Wahrscheinlichkeit halt richtige Entscheidungen und insofern kann er schon irgendwo ein role model sein. Aber die Frage ist, ob man ihn dann jemals erreichen könnte sozusagen? Weil er halt immer weniger Fehler machen würde sozusagen. Und ich glaube, ein normaler Mensch könnte das halt nicht. #00:07:21#

**Interviewer:**

Also würdest du sagen, er kann schon ein Vorbild sein? #00:07:24#

**Interviewpartnerin:**

Aber unerreichbar. #00:07:26#

**Interviewer:**

Okay. Aber halt in seiner Arbeit, dass er halt sehr gut arbeitet und das wäre dann als Vorbild zwar unerreichbar, aber immerhin Vorbild für die Mitarbeiter? #00:07:37#

**Interviewpartnerin:**

Ja, vielleicht ist Vorbild auch das falsche Wort. (überlegt länger) Also ich weiß nicht. Vorbild hört sich nicht so richtig an dafür. #00:07:54#

**Interviewer:**

Sondern? #00:07:54#

**Interviewpartnerin:**

Ich überlege gerade noch. Ja, keine Ahnung. Vorbild geht in die richtige Richtung, aber Vorbild ist auch jemand für mich, von dem ich weiß: Wenn ich nur stark genug wollen würde, dann könnte ich genauso werden. Aber ich kann ja niemals ein Roboter werden. #00:08:20#

**Interviewer:**

Also dadurch, dass es nicht erreichbar ist, ist er auch nicht wirklich ein Vorbild? #00:08:25#

**Interviewpartnerin:**

Das ist nicht so krass motivierend, muss ich sagen, wenn ich weiß, ich kann eh nie so gut sein wie er. #00:08:30#

**Interviewer:**

Ja, dann siehst du, wie gut er arbeitet und sagst: Ja, schaffe ich eh nicht. #00:08:38#

**Interviewpartnerin:**

Also motivierend, weiß ich nicht, ob das für mich motivierend wäre. Ich glaube, eher nicht. #00:08:43#

**Interviewer:**

Dadurch, dass es nicht erreichbar ist? #00:08:44#

**Interviewpartnerin:**

Ja. #00:08:46#

**Interviewer:**

Wenn du jetzt in einem Team wärst mit Pepper als Führungskraft: Würdet ihr gemeinsame Ziele anstreben, die von Pepper gesetzt werden? Würdet ihr das so aktiv machen oder eher / #00:09:08#

**Interviewpartnerin:**

Also Pepper ist quasi der Teamlead? #00:09:10#

**Interviewer:**

Genau. Und er würde dann für das ganze Team Ziele setzen. Meinst du, ihr würdet dann drauf hinarbeiten oder eher / #00:09:20#

**Interviewpartnerin:**

Ich denke schon. Er weiß ja, was er tut. Im Grunde genommen ist auch nur wieder irgendwie ein Algorithmus dahinter, der das dann rausfindet, was man tun sollte. Und Pepper ist halt jetzt einfach nur eine Darstellungsform, um das halt zu präsentieren. Man könnte es auch einfach am Bildschirm anschauen sozusagen, denke ich mal; die Ergebnisse, was man halt tun sollte. #00:09:47#

**Interviewer:**

Also würdest du sagen, dadurch dass es durch einen Algorithmus oder was Ähnliches / #00:09:55#

**Interviewpartnerin:**

Dadurch, dass es halt / Deswegen meinte ich vorhin das auch so mit Robotern und Menschen so: Dadurch, dass ich halt / also ich persönlich weiß ja, dass es halt kein Mensch ist sozusagen, der da drin ist und sich / dass da halt wirklich irgendwie ein Algorithmus dahintersteckt, der halt auch die richtigen Ergebnisse liefern kann. Mir persönlich ist es egal, ob ich das jetzt am Bildschirm sehe oder mit so einem Roboter. #00:10:19#

**Interviewer:**

(unv. #00:10:22#)

**Interviewpartnerin:**

Ja. #00:10:22#

**Interviewer:**

Aber du glaubst halt, dadurch dass es eine Maschine errechnet hat, diese Ziele, dann sind das auch die richtigen Ziele und dann würdet ihr auch darauf hinarbeiten? #00:10:32#

**Interviewpartnerin:**

Ich denke schon . Also in dieser spezifischen Situation ohne irgendwelche anderen Anforderungen oder so was oder Einflüsse, ja. #00:10:42#

**Interviewer:**

Inwiefern glaubst du, dass Pepper hohe Erwartungen an euch als Team hätte? #00:10:49#

**Interviewpartnerin:**

(überlegt etwas). Du meinst, ob er hohe Erwartungen hätte oder / #00:10:59#

**Interviewer:**

Also, ja. Also inwiefern, wie hoch wären die Erwartungen? #00:11:05#

**Interviewpartnerin:**

Ich weiß nicht. Es ist ja eigentlich egal, weil er kann eh nicht bestrafen, oder? Wenn die Erwartungen nicht erfüllt werden, was passiert dann? #00:11:18#

**Interviewer:**

Ich meine, wie ein Chef würde er dann wahrscheinlich auch Krit / #00:11:23#

**Interviewpartnerin:**

Ausrasten (lacht). #00:11:27#

**Interviewer:**

Vielleicht nicht ausrasten, aber schon Kritik äußern und es stehen ihm wahrscheinlich auch dieselben Bestrafungsinstrumente, die einem anderem Führer oder halt Leader zur Verfügung stehen. #00:11:41#

**Interviewpartnerin:**

Ich glaube, er würde schon tendenziell hohe Ziele setzen oder Erwartungen haben. #00:11:57#

**Interviewer:**

Wieso? #00:11:58#

**Interviewpartnerin:**

Was bringt es, wenn man niedrige Erwartungen hat oder Ziele setzt? Und ich denke halt, wie gesagt, es ist halt / wenn der Algorithmus halt so die Situation halt optimieren möchte, dann wird er auf jeden Fall irgendwo auch berücksichtigen, dass es halt mehr Sinn macht, höhere Ziel oder Erwartungen zu stellen als niedrige. #00:12:26#

**Interviewer:**

Also dadurch, dass er immer versucht, die Situation zu optimieren, hat er auch entsprechend hohe Erwartungen? #00:12:33#

**Interviewpartnerin:**

Mhm (zustimmend). #00:12:34#

**Interviewer:**

In welchem Ausmaß, glaubst du, dass Pepper dich als Teammitglied angemessen in deiner Arbeit unterstützen kann? #00:12:43#

**Interviewpartnerin:**

(überlegt länger) Also ich glaube, ich könnte jetzt keine emotionale irgendwie Bindung zu ihm aufbauen. Insofern wird das mit der Motivation ein bisschen schwierig. Wenn ich jetzt irgendwelche harte Fakten oder Zahlen oder so was sehen würde als Ergebnis meiner Arbeit und mir dann von ihm gesagt wird: „Ja, du musst“, keine Ahnung, „nur noch fünf Apps verkaufen, dann haben wir das Verkaufsziel erreicht“, sage ich jetzt mal. Das vielleicht schon, aber alles, was dann halt so auf emotionaler Ebene passiert: Ich glaube nicht, dass er das rüberbringen könnte, um mich dahingehend zu unterstützen. Ich glaube, ich habe deine Frage nicht ganz beantwortet, also bin nicht so ganz drauf eingegangen. #00:13:42#

**Interviewer:**

Also wenn er dir jetzt eine Aufgabe geben würde, könnte er dich dabei auch unterstützen? #00:13:49#

**Interviewpartnerin:**

Ich glaube, tendenziell schon. #00:13:57#

**Interviewer:**

Bei was könnte er dich nicht unterstützen? #00:14:00#

**Interviewpartnerin:**

Also wie gesagt, wenn es halt irgendein Problem gibt, zum Beispiel ich kann irgendwas nicht ausrechnen oder so, dann kann er mich sicherlich unterstützen. Aber wenn ich jetzt irgendwie so voll fertig bin oder was, emotional, und deswegen die Aufgabe nicht lösen kann, dann weiß ich nicht, ob er mich dabei unterstützen könnte wie ein normaler Mensch. #00:14:22#

**Interviewer:**

Meinst du, er würde darauf irgendwie Rücksicht nehmen? Meinst du, er merkt: Okay, die Simone hat heute Nacht so schlecht geschlafen; dann muss man sie nicht so nerven oder so, solche Sachen. #00:14:41#

**Interviewpartnerin:**

Sicherlich möglich. Also ich weiß nicht, wie weit jetzt er programmiert ist. Oder so was ist sicherlich möglich, dass man das erkennen kann. #00:14:52#

**Interviewer:**

Also grundsätzlich möglich? #00:14:53#

**Interviewpartnerin:**

Grundsätzlich möglich. #00:14:55#

**Interviewer:**

Das ist eigentlich auch schon ein bisschen die nächste Frage: Inwiefern hast du das Gefühl, dass Pepper sich an dich anpasst? Also zum Beispiel eben Rücksicht auf persönliche Bedürfnisse nimmt? Vielleicht auch, wenn ein Mitarbeiter mehr Unterstützung braucht als ein anderer. Solche Sachen halt. #00:15:19#

**Interviewpartnerin:**

Also ich denke, dass es schon möglich ist, dass es halt grundsätzlich funktionieren kann. Also dass man anhand der Körpersprache des Anderen halt dann oder anhand der Mimik dann halt erkennen kann, wie er grade so drauf ist. Ob jemand halt viel lacht oder halt irgendwie so komisch dasitzt und nach vorne gebeugt oder ganz aufrecht dasitzt oder so. Ich denke, daran / Also ich denke, das kann man dann schon irgendwie erkennen und dann kann man das irgendwie berücksichtigen. #00:15:54#

**Interviewer:**

Wäre also auch möglich, dass er da Rücksicht nimmt. Inwiefern hat dich Pepper angeregt, neue und innovative Ideen zu produzieren? #00:16:04#

**Interviewpartnerin:**

(lacht leise) Gar nicht. Keine Ahnung. Nicht mehr, als irgendwas anderes auch. Ich kann dazu nicht so viel sagen (lacht), glaube ich. #00:16:20#

**Interviewer:**

Okay. (unv. #00:16:22#9

**Interviewpartnerin:**

Also die Aufgabe zum Beispiel, also wenn es halt / wenn ich das halt einfach nur gelesen hätte, was er jetzt gesagt hat oder so, dann hätte ich, denke ich, das Gleiche geantwortet. #00:16:34#

**Interviewer:**

Okay. Und in Kombination auch mit diesen neuen und innovativen Ideen: Denkst du, Pepper würde ich ermutigen, den Status quo anzuzweifeln? Jetzt zum Beispiel, wenn du für ihn arbeiten würdest, so Arbeitsprozesse anzuzweifeln oder halt, wie man arbeitet. Solche Sachen. #00:16:59#

**Interviewpartnerin:**

Das sicherlich. Aber ich weiß nicht, ob das nur ist, weil er ein Roboter ist oder ob das nicht halt durch irgendwas anderes auch ausgelöst werden kann. #00:17:12#

**Interviewer:**

Also meinst du, schon dadurch, dass er ein Roboter ist, fängt man an, so ein bisschen die Sachen zu hinterfragen? #00:17:21#

**Interviewpartnerin:**

Ja, aber ich bin mir nicht sicher, ob / Also ich würde nicht ausschließen, dass es andere Dinge nicht auch könnten. Also wenn ich jetzt irgendwie einen inspirierenden Menschen oder von (unv. #00:17:30#) redet, ob mich das nicht auch zum Umdenken bringen könnte oder zum Anzweifeln oder so was. Das würde ich nicht ausschließen. #00:17:38#

**Interviewer:**

Würdest du für Pepper beziehungsweise für das Ziel, das von Pepper definiert wurde, mehr als das zwingend Notwendige tun oder einfach nur das, was erwartet wird? #00:17:56#

**Interviewpartnerin:**

Also ich würde auf jeden Fall / Also ich glaube, das bin aber dann wahrscheinlich nur ich, dass ich auf jeden Fall versuche, so ein bisschen mehr zu geben sozusagen als erwartet wird. Ja. #00:18:18#

**Interviewer:**

Also schon mehr Einsatz zeigen und ein bisschen mehr / #00:18:22#

**Interviewpartnerin:**

Ja, aber wie gesagt, ich weiß halt nicht, ob das jetzt genau ist, weil er das so präsentiert hat oder so. #00:18:29#

**Interviewer:**

Wie war dein Gesamteindruck von Pepper? #00:18:33#

**Interviewpartnerin:**

Nett und sympathisch, aber ein bisschen gruselig, wenn er so rumsteht und was macht oder gar nichts macht. Keine Ahnung. Eigentlich schaut er auch voll süß aus wegen den großen Augen. #00:18:47#

**Interviewer:**

(lacht kurz) #00:18:50#

**Interviewpartnerin:**

Das hilft jetzt wahrscheinlich nicht so wahnsinnig (lacht). #00:18:53#

**Interviewer:**

Nein, das ist gut. Ja, ich meine, vom Aussehen ist er halt süß und sympathisch. #00:19:01#

**Interviewpartnerin:**

Ja. #00:19:01#

**Interviewer:**

Inwiefern hast du dich von Pepper motiviert gefühlt? #00:19:05#

**Interviewpartnerin:**

(überlegt länger) Ja, also ich fand, was er am Ende gesagt hat, fand ich ganz gut. Also das mit dem „Ich hoffe, dass ihr genauso viel Anstrengungen zeigt, wie ich halt auch zeige“ und so, was er am Ende gesagt hat. Das davor war halt nur reine Information. Insofern hat mich das / die Worte haben mich jetzt schon motiviert so. #00:19:37#

**Interviewer:**

Du glaubst halt auch, eher die Worte (unv. #00:19:43#)

**Interviewpartnerin:**

Ja. #00:19:43#

**Interviewer:**

Das, was er gesagt hat. Fühlst du dich von Pepper wertgeschätzt? #00:19:50#

**Interviewpartnerin:**

Nein (lacht). Ich fühle mich auch nicht so, als ob er mich so wirklich anschauen würde. Also irgendwie schaut er einen an, aber irgendwie auch nicht, weil halt keine Augenbewegung da ist. Er kann ja nur seinen Kopf bewegen und irgendwie, weiß ich nicht. Wie gesagt, ich könnte halt nicht irgendwie eine emotionale Verbindung aufbauen zu ihm, also auch nicht irgendwie so das Gefühl der Wertschätzung bekommen. Ich glaube nicht, nein. #00:20:21#

**Interviewer:**

Auch wenn du für ihn arbeiten würdest, meinst du, er würde irgendwie wertschätzen die Arbeit, die du machst oder auch nicht? #00:20:31#

**Interviewpartnerin:**

Ich weiß nicht, vielleicht tut er es. Aber ich glaube, ich würde nicht das Gefühl bekommen, dass ich oder dass meine Arbeit wertgeschätzt wird. #00:20:40#

**Interviewer:**

Denkst du, Pepper ist fair zu seinen Teammitgliedern? #00:20:45#

**Interviewpartnerin:**

Mhm (zustimmend). #00:20:47#

**Interviewer:**

Ja? #00:20:47#

**Interviewpartnerin:**

Ja. #00:20:48#

**Interviewer:**

Wieso? #00:20:49#

**Interviewpartnerin:**

Ich denke schon, weil er unterscheidet ja nicht, wen er vor sich hat. Jedes Objekt ist erst mal ein Objekt und jeder Mensch ist erst mal ein Mensch, oder, bei ihm? #00:21:01#

**Interviewer:**

Ja klar. #00:21:02#

**Interviewpartnerin:**

Und wahrscheinlich wird er auch nur auf Basis von Leistungen bewerten können. Insofern wird es schon irgendwo fair sein. #00:21:09#

**Interviewer:**

Also dadurch, dass er nüchtern einfach nur guckt, wie deine Leistung ist im Vergleich zu anderen Mitarbeitern, ist er so weit fair? Hat auch keine Lieblingsmitarbeiter oder solche Sachen? #00:21:21#

**Interviewpartnerin:**

Nein. Also ich denke schon, dass er weitestgehend fair wäre. #00:21:26#

**Interviewer:**

Kannst du Pepper als Führungskraft vertrauen? #00:21:29#

**Interviewpartnerin:**

(etwas zögerlich) Ja (lacht), irgendwie schon bis zu einem gewissen Grad, aber irgendwie, ja, es ist schon komisch. Ich denke mir, dass er weiß, was er tut sozusagen. Insofern kann ich schon vertrauen. Aber – ja, ja, geht so wahrscheinlich. #00:22:10#

**Interviewer:**

Also du gehst schon davon aus, dass das, was er macht, richtig ist und du würdest ihm dann auch da vertrauen, was er sagt? #00:22:20#

**Interviewpartnerin:**

Ich denke schon. Ich wäre wahrscheinlich immer skeptisch, ich hätte immer Zweifel oder so. Aber letzten Endes, wenn er so die Führungskraft wäre, dann / Was ist das überhaupt für eine Führungskraft? Also sorry. Du stellst da einfach so einen Roboter hin, anstatt jemand, der halt sich so vielleicht hochgearbeitet hat oder der halt zumindest so von der Wahrnehmung her mehr Ahnung hat als so ein Roboter. Weil theoretisch weiß der Roboter ja, wie gesagt, nur so viel, wie du ihm halt vorher beigebracht hast oder gesagt hast oder so. #00:23:08#

**Interviewer:**

Ja. #00:23:08#

**Interviewpartnerin:**

Und deswegen, ich weiß nicht, ob / Also den könntest du halt als Führungskraft hinstellen, den könntest du aber auch als normalen Mitarbeiter hinstellen sozusagen. Der ist austauschbar, weißt du. Und Menschen sind nicht austauschbar. #00:23:22#

**Interviewer:**

Ich meine, auch ein Führungskraft, eine menschliche, weiß ja eigentlich auch nur das, was sie gelernt hat. #00:23:30#

**Interviewpartnerin:**

Aber da gibt es so einen Lebenslauf dahinter. Ich glaube, das ist was, was den Mensch als Führungskraft sympathischer macht als den Roboter als Führungskraft, dass das wirklich irgendwo was dahintersteht, was man auch selber nachvollziehen kann, anstatt einer Technologie. Dass da halt auch jemand mit so echten Erfahrungen dahintersteht. #00:23:55#

**Interviewer:**

Also wenn er jetzt dein Chef wäre, würdest du so ein bisschen, weiß nicht, die Lebensgeschichte dahinter / würde dir fehlen so? #00:24:09#

**Interviewpartnerin:**

Mhm (zustimmend). Deswegen meinte ich vorhin auch so mit dem Ernstnehmen. Irgendwie ist es halt ein Roboter. Einfach niemand, der so / Niemand, wenn man jetzt von einer Person sprechen könnte, niemand, der scheinbar wirklich eine Ahnung hat von dem, was er tut. Natürlich, ich bin mir sicher, dass er Ahnung hat von dem, was er tut. Aber deswegen das Vertrauen auch so, ja, bis zu einem gewissen Grad schon. Aber ich weiß nicht. Du vertraust auch nicht jedem Menschen, nur weil er das Richtige macht oder so. #00:24:40#

**Interviewer:**

Ja. #00:24:42#

**Interviewpartnerin:**

Also nur, weil jemand immer das Richtige macht, heißt das nicht, dass du ihm bedingungslos vertraust oder zu 100 % vertraust und so wird es da auch sein. #00:24:50#

**Interviewer:**

Aber ich meine, wenn du für ihn arbeitest und er länger und er eigentlich immer die richtige Entscheidung trifft, würde das für dich nicht irgendwie Vertrauen aufbauen? #00:25:06#

**Interviewpartnerin:**

Wie gesagt, Vertrauen in die Sache an sich, weil er halt sicherlich sehr, sehr oft die richtigen Entscheidungen treffen wird oder Vorgaben machen wird. In der Hinsicht schon. Aber so irgendwie / Ich weiß nicht, man kann den Begriff „Vertrauen“ jetzt natürlich ziemlich weit fassen. Alles, was darüber hinausgeht, wahrscheinlich eher nicht. #00:25:32#

**Interviewer:**

Welche Verhaltensweisen waren dir sympathisch? #00:25:38#

**Interviewpartnerin:**

Die Körperbewegungen von Kopf und Armen und nach vorne beugen und wieder zurück. Also das Gesicht sieht halt schon relativ menschlich aus mit den Augen, Nase und Mund. Wie gesagt, also die Augen sind halt groß, das ist so süß einfach irgendwie. Wenn irgendwas süß aussieht, dann ist es nicht unsympathisch oder so. Und irgendwie lächelt er ja auch die ganze Zeit. Von daher. #00:26:13#

**Interviewer:**

Ja, stimmt. Was hat dich an seinem Verhalten gestört? #00:26:16#

**Interviewpartnerin:**

Am Verhalten jetzt? #00:26:23#

**Interviewer:**

Mhm (zustimmend). Oder auch an ihm. #00:26:26#

**Interviewpartnerin:**

Also wie gesagt, die Stimme war ein bisschen störend und anstrengend zum lange Zuhören. Aber ich schätze, das hat man gemacht, damit man halt weder eine Männer-, noch eine Frauenstimme hört. Und was hat mich noch gestört an seinem Verhalten? Also jetzt richtig gestört, wäre jetzt irgendwie falsch gesagt. Aber was halt so gefehlt hat, war halt wie gesagt, die Mimik. #00:26:55#

**Interviewer:**

Also ein Gesicht? #00:26:56#

**Interviewpartnerin:**

Ja. #00:26:59#

**Interviewer:**

Wie würde es dir jetzt gehen, wenn Pepper dein Chef wäre? #00:27:02#

**Interviewpartnerin:**

Wie es mir jetzt gehen würde? #00:27:04#

**Interviewer:**

Also wie es dir gehen würde? #00:27:07#

**Interviewpartnerin:**

(überlegt länger) Weiß ich nicht. Er ist halt nicht so der typische Chef. Also er ist halt erst mal viel zu klein meiner Meinung nach und sieht viel zu süß aus wegen den großen Augen. Okay, das stört mich vielleicht noch an ihm (lacht etwas). So nach dem Vortrag zum Beispiel, wie es mir gehen würde? #00:27:45#

**Interviewer:**

Wenn du dir jetzt vorstellst, du bist in Peppers Abteilung, Pepper ist jetzt dein Chef. Vielleicht auch, was gut funktionieren würde, was schlecht funktionieren würde. #00:27:58#

**Interviewpartnerin:**

Ich kann mir das schwer vorstellen, ehrlich gesagt. Ich glaube, ich fände es erst mal richtig komisch. Also es braucht wahrscheinlich eine lange Eingewöhnungszeit bis es halt voll akzeptiert wird. Wie gesagt, dieses Vertrauen da ist. Es müssen halt immer wieder die richtigen Ergebnisse kommen. Das dauert halt auch seine / braucht auch seine Zeit. Ich glaube, ich wäre erst mal richtig misstrauisch. Ja. Und skeptisch. Ich würde es wahrscheinlich ausprobieren wollen, aber ich wäre sehr misstrauisch. Und das würde sich wahrscheinlich auf meine Arbeitsweise darstellen irgendwie, dass ich halt / Also es ist halt / Nehmen wir an, ich komme jetzt neu in die Abteilung oder er kommt neu in die Abteilung und verteilt so Aufgaben oder so was. Und es ist halt was Neues, es ist etwas komplett Anderes irgendwie und man versucht ja immer so alles, was neu ist, erst mal so wegzustoßen und irgendwie schlecht zu machen und so, weil so, wie es jetzt ist, ist es ja eh am besten. Genau. Deswegen wird wahrscheinlich die Performance auch, schätze ich mal, vom Team halt sinken. Also stark sinken, denke ich. Und dann, erst wenn man so merkt: Ja, der kann doch irgendwie was sozusagen. Dann fängt auch an, die Teamperformance wieder zu steigen und auch vielleicht die eigene Motivation für die Aufgaben, die man bekommt. Also ich glaube, ich für mich selber, ich wäre erst mal / würde erst mal schlechter performen auch und dann wieder besser, wenn ich sehe, dass es was bringt. #00:30:26#

**Interviewer:**

Also mit der Zeit, wenn er weiterhin gut arbeitet und richtige Entscheidungen trifft, dann würde man sich dann auch wieder mehr performen. #00:30:35#

**Interviewpartnerin:**

Ja. #00:30:36#

**Interviewer:**

Und was meinst du, was mit Pepper als Chef schlecht funktionieren würde? Also wo könnten seine Limitationen sein? #00:30:47#

**Interviewpartnerin:**

Also wie gesagt, ich kann mir vorstellen, dass halt, wenn es schlecht läuft, dass er dann nicht so gut umgehen kann oder gar nicht damit umgehen kann, weil bei so bestrafen, ich weiß nicht: Bestrafen ist immer so ein bisschen heikel für mich, weil ganz oft muss man halt noch ganz viele andere Dinge mit einbeziehen als nur die Leistung, die halt nicht erbracht wurde oder ungenügend erbracht wurde. Und ich glaube, das kann er nicht. #00:31:18#

**Interviewer:**

Was für Faktoren dann? Irgendwie Lebenssituation oder / #00:31:24#

**Interviewpartnerin:**

Ich weiß nicht, manchmal hat man ja auch einfach nur so ein Gefühl sozusagen. Weißt du, was ich meine? Manchmal hat man irgendwie so ein Gefühl, dass man eine bestimmte Sache machen sollte oder nicht machen sollte und man kann es als Mensch vielleicht selber auch gar nicht mal erklären. Und ich glaube halt, das kann er nicht in solchen Situationen das / #00:31:42#

**Interviewer:**

Also so ein Bauchgefühl? #00:31:42#

**Interviewpartnerin:**

Ja, sozusagen. Oder so Intuition, oder man kann es nennen, wie man will. #00:31:49#

**Interviewer:**

Das würde dann fehlen? #00:31:50#

**Interviewpartnerin:**

Mhm (zustimmend). #00:31:51#

**Interviewer:**

Wenn er sehr strenges Feedback gibt, wie würdest du damit umgehen? #00:32:02#

**Interviewpartnerin:**

Ich glaube, es würde mich irgendwo doch motivieren, noch besser zu werden (lacht). Ich glaube schon. #00:32:18#

**Interviewer:**

Also würdest du es schon annehmen und dann dran arbeiten? #00:32:22#

**Interviewpartnerin:**

Ja. #00:32:24#

**Interviewer:**

Wie gut, meinst du, kann Pepper für die Zukunft seines Teams Pläne erstellen und Ziele setzen? #00:32:29#

**Interviewpartnerin:**

Ganz gut. #00:32:32#

**Interviewer:**

Okay. Wieso? #00:32:33#

**Interviewpartnerin:**

Er weiß doch, was er tut, oder? (lacht) Ich denke mal, er wird schon die ganzen Daten richtig verarbeiten. #00:32:53#

**Interviewer:**

Also jetzt auch zukunftsgerichtet, dass er so ein bisschen planen kann? #00:32:58#

**Interviewpartnerin:**

Mhm (zustimmend). #00:33:00#

**Interviewer:**

Würde er schon schaffen? #00:33:03#

**Interviewpartnerin:**

Ja. So grundsätzlich schon. #00:33:06#

**Interviewer:**

Die letzte Frage: Was würdest du vermuten, wie erfolgreich wäre ein Team, das von Pepper geführt wird? #00:33:13#

**Interviewpartnerin:**

Also ich glaube, es würde nicht überdurchschnittlich / Also wie gesagt, ich glaube, so über die Zeit gesehen wird es eher durchschnittlich sein, aber nur, weil am Anfang halt die Performance runtergeht und dann wieder hoch. Ich glaube halt, er kann bessere Vorhersagen treffen, als irgendwie ein Mensch, sofern sich nicht selbst dann auch irgendwelcher Algorithmen oder Programme bedient. Irgendwann wird es vielleicht überdurchschnittlich sein, die Leistung oder die Performance. Aber so, wenn man die gesamte Zeitspanne nimmt, dann wahrscheinlich doch eher wieder durchschnittlich. #00:33:57#

**Interviewer:**

Also am Anfang erst mal unterdurchschnittlich und dann über die Zeit immer besser? #00:34:02#

**Interviewpartnerin:**

Immer besser und dann vielleicht sogar überdurchschnittlich. #00:34:05#

**Interviewer:**

Also braucht er etwas Zeit zu lernen? #00:34:07#

**Interviewpartnerin:**

Mhm (zustimmend). Auch das Team. #00:34:11#

**Interviewer:**

Auch das Team, sich an ihn anzupassen? #00:34:12#

**Interviewpartnerin:**

Genau und sich an ihn zu gewöhnen. #00:34:17#

**Interviewer:**

Okay.
