# Supplementary material for: Robot leadership–Investigating human perceptions and reactions towards social robots showing leadership behaviors
Source: PLoS One. 2023 Feb 16;18(2):e0281786. doi: 10.1371/journal.pone.0281786 (PMC9934409; doi:10.1371/journal.pone.0281786)
Supplement: S1 Dataset — (ZIP) [file pone.0281786.s002.zip › Interview_Participant5.doc]

**15 Interview**

**Interviewer:**

So als Prämisse stelle dir jetzt vor, du arbeitest in einem Team mit Pepper als Führungskraft. Welche Gedanken sind dir durch den Kopf gegangen, während Pepper mit dir geredet hat? #00:00:16#

**Interviewpartner:**

Ziemlich innovatives Konzept, Körpersprache und / Ja, Körpersprache vor allem ist ziemlich ausgeprägt schon, was ziemlich cool ist. Sprache an sich war ehrlich gesagt rein von den anderen AIs draußen oder von den anderen, ja, wie Alexa, Google Home und so weiter, ziemlich zurückgeblieben. Also das ist ziemlich undeutlich noch gewesen. Was vorteilhaft war, war natürlich der Display. Da hat man relativ gut folgen können. Ja, das ist mir durch den Kopf gegangen. Was ist mir sonst noch durch den Kopf gegangen? Ja, das war es eigentlich. #00:01:01#

**Interviewer:**

Wie hat es sich jetzt für dich angefühlt, von Pepper angesprochen zu werden? #00:01:06#

**Interviewpartner:**

Ja, im ersten Moment bist du natürlich überrascht, aber ich bin mittlerweile schon so gewohnt, dass irgendwelche Computer oder Maschinen mit mir reden, also war es jetzt nicht wirklich so: Oh mein Gott! Jetzt redet ein Roboter mit mir! Sondern du kommst eigentlich relativ schnell rein, wenn du es einigermaßen gewohnt bist. #00:01:23#

**Interviewer:**

Okay. Was für Maschinen: Handy / #00:01:25#

**Interviewpartner:**

Handy, Alexa. Ich nutze Alexa, glaube ich, was weiß ich, 30mal am Tag. Also bin ich es eigentlich eh gewohnt, dass einfach irgendwelche Computer mit mir reden. Und ich habe auch meine ganzen Applikationen / lasse ich eigentlich mittlerweile per Steuerung steuern, also per Sprachsteuerung steuern oder per irgendwelche smart touches. Also ist das eigentlich nicht so drastisch jetzt für mich gewesen. Ich bin das so eigentlich ziemlich gewohnt. #00:01:49#

**Interviewer:**

Was hast du gedacht, als Pepper „We do not tolerate any mistakes“ gesagt hat? #00:01:53#

**Interviewpartner:**

Mein Gott, das ist jetzt Einstellung, wie man sich (unv. #00:02:00#) für sich selber ausmachen. Das mache ich jetzt nicht an der Pepper fest. Also da ist mir eigentlich egal, ob mir das jetzt ein Mensch sagt oder sie. Also das ist viel mit Einstellungssache. Wenn halt das Team das so meint, dann muss halt das Team damit klarkommen, dass keine Fehler toleriert werden, was nicht unbedingt die beste Einstellung ist. #00:02:20#

**Interviewer:**

Okay. Aber ist für dich eher dann organisationsabhängig? #00:02:25#

**Interviewpartner:**

Das ist organisationsabhängig. Da kann jetzt Pepper nichts dafür. #00:02:29#

**Interviewer:**

Was denkst du, wie Pepper reagieren würde, wenn du einen Fehler machst? #00:02:34#

**Interviewpartner:**

(überlegt etwas) Ich glaube, wahrscheinlich weniger einfühlsam wie normale Führungskräfte. Und, ja, das ist jetzt schwierig, weil ich nicht weiß, wie es programmiert ist. Aber wahrscheinlich würde ich mir ehrlich gesagt schlechter vorkommen wie wenn ein Mensch das (gesagt? #00:03:03#) hätte. Also ich würde wahrscheinlich versuchen, weniger Fehler zu machen und weniger experimentierfreudig zu sein, als / #00:03:10#

**Interviewer:**

Okay. Wieso? Meinst du, er ist dann / #00:03:13#

**Interviewpartner:**

Weil Fehler passieren ja nicht einfach so, sondern der hat ja relativ viele Einflussfaktoren. Ich glaube ehrlich gesagt nicht, dass die AI von Pepper schon so weit ausgereift ist, dass sie das komplexe Problem versteht, warum ich jetzt den Fehler begangen habe. #00:03:27#

**Interviewer:**

Okay. Also meinst du, er kann nicht wirklich nachvollziehen, wieso du den Fehler gemacht hast? Okay. #00:03:34#

**Interviewpartner:**

Weil ich mache keine leichten Fehler. #00:03:37#

**Interviewer:**

Pepper hat auch gesagt: „You should document what you do so that I am able to follow your steps and identify mistakes. This will allow me, go give you exact feedback on your work.” Hast du dich bewusst an diese Vorgabe gehalten? #00:03:51#

**Interviewpartner:**

Nein, weil ich das nie mache. #00:03:56#

**Interviewer:**

Okay. Wieso hast du dich nicht / #00:03:59#

**Interviewpartner:**

Weil ich nie alles von mir dokumentiere, weil es mir viel zu viel Zeit wegnimmt. #00:04:05#

**Interviewer:**

Okay. Einfach zu viel Aufwand? #00:04:06#

**Interviewpartner:**

Das wäre für mich zu viel Aufwand einfach. Das mache ich aber auch / Also das macht jetzt nicht nur / auch wenn mir mein Vorgesetzter sagt, ich soll alles dokumentieren, mache ich das nicht. Das hat jetzt nichts mit Pepper oder so zu tun, das ist einfach eine persönliche Arbeitseinstellung, weil ich es nicht einsehe, dass ich so viel Zeit für so was verwende, was ich dann sowieso wieder verschmeiße am Ende. #00:04:24#

**Interviewer:**

Wie war dein Gesamteindruck von Pepper? #00:04:26#

**Interviewpartner:**

Eher leicht positiv. Also ich finde es nicht schlecht, dass man über so was nachdenkt. Ich bin mir vielleicht nicht sicher, ob es wirklich der ideale Weg ist sozusagen, komplette Führungskraft vom Marketing oder so zu ersetzen. Aber so als Assistenz oder so, könnte ich mir durchaus vorstellen. #00:04:50#

**Interviewer:**

Okay. Und jetzt wieso gerade vom Marketing? #00:04:54#

**Interviewpartner:**

Marketing, finde ich, ist relativ / Das ist nicht analytisch getrieben. Marketing ist eine Sache, wo du relativ viel Fingerspitzengefühl haben musst, viel Erfahrung haben musst und da ist AI einfach noch nicht so weit. Es gibt viele andere Sachen wie Business Devolopment, Finances, da hätte ich gar kein Problem, weil das einfach nur stupide irgendwelche Sachen ausrechnen ist. Es sind irgendwie KPIs, vergleiche ich, irgendwie schauen, wie ich es runterrechnen kann. Das kannst du mit big data im Hintergrund alles machen. Aber Marketing ist so eine Sache, da spielen so viele Einflussfaktoren eine Rolle, dass ich es relativ schwierig finde, mit einem, also zu dem derzeitigen Zeitpunkt noch, AI das (unv. #00:05:36#)

**Interviewer:**

Also würdest du es eher bei so analytischen Sachen sehen? #00:05:39#

**Interviewpartner:**

Ich würde es bei analytischen Sachen sehen, ja. #00:05:40#

**Interviewer:**

Inwiefern hast du dich von Pepper motiviert gefühlt? #00:05:44#

**Interviewpartner:**

Inwiefern habe ich mich von Pepper motiviert gefühlt? Na ja, wenn ich ehrlich bin, nicht wirklich. #00:05:57#

**Interviewer:**

Okay. Wieso? #00:06:00#

**Interviewpartner:**

Ja, sie sagt halt: „Mach das, mach das, mach das, mach das, mach das.“ Aber ich habe jetzt nicht irgendwie gesehen, was ich für Vorteile draus bekommen hätte. Aber das weiß ich jetzt nicht, ob das wirklich an Pepper lag oder einfach an dem / Wenn mir jetzt ein Mensch das gesagt hätte, glaube ich, wäre ich auch nicht motiviert gewesen. Also mir hat jetzt die intrinsische Motivation gefehlt, weil ich jetzt nicht gesehen habe, was ich für einen Vorteil habe, wenn ich jetzt da ein Marketingkonzept für die aufschreibe. Oder (unv. #00:06:29#) beschäftigt. #00:06:32#

**Interviewer:**

Okay. Und wenn du dir jetzt vorstellst, du wärst in seinem Team: Würdest du dich von ihm motiviert fühlen oder / #00:06:37#

**Interviewpartner:**

Nein, immer noch nicht. Weil ich jetzt nicht sehe, was für mich da rausspringen würde. #00:06:43#

**Interviewer:**

Okay. Also fehlt dir da so ein bisschen / #00:06:45#

**Interviewpartner:**

Ich bin relativ egozentrisch getrieben und (unv. #00:06:48#) wenn ich ihm jetzt das liefere und er mir irgendwelche Bilder sagt. Toll, was habe ich jetzt davon? #00:06:53#

**Interviewer:**

Okay. Fühlst du dich von Pepper wertgeschätzt? #00:06:58#

**Interviewpartner:**

Ich habe jetzt bisher noch kein Feedback von ihr erhalten, also das ist relativ schwierig. Sie weiß ja nicht, wie ich bin. #00:07:07#

**Interviewer:**

Okay. Und wenn du dir jetzt wieder vorstellst, du würdest für ihn arbeiten: Meinst du, er kann dich wertschätzen? #00:07:14#

**Interviewpartner:**

Glaube ich schon. Aber das kommt jetzt echt drauf an. Also Wertschätzung verdient man sich, das kann man jetzt nicht nach einem Gespräch in dem Sinn festmachen. Wertschätzung verdient man sich. #00:07:27#

**Interviewer:**

Verdient man sich wie? #00:07:27#

**Interviewpartner:**

Also indem man halt Leistung abliefert. Und wenn jetzt Pepper bei mir sieht, dass ich besser bin wie der Rest, dann wird sie mich sicherlich auch mehr wertschätzen. #00:07:34#

**Interviewer:**

Okay. Also vielleicht, wenn du es mit einem Menschen vergleichst: Meinst du, er kann da genauso gut dich wertschätzen? #00:07:44#

**Interviewpartner:**

Vielleicht nicht genauso gut, weil ich auch relativ viel gute soft skills habe. Aber im allgemeinen von meiner Arbeit her: Ich glaube schon. #00:07:52#

**Interviewer:**

Denkst du, Pepper ist fair zu seinen Teammitgliedern? #00:07:56#

**Interviewpartner:**

Boi, das sind Fragen! Pepper ist fair zu seinen Teammitgliedern. Nein. #00:08:07#

**Interviewer:**

Wieso nicht? #00:08:08#

**Interviewpartner:**

Weil Pepper ja am Schluss eigentlich nur die ganzen steps sieht und wie das Ergebnis ist sozusagen. Also sie sieht jetzt zum Beispiel nicht, wenn wir jetzt zum Beispiel im Team zusammen sind, wenn zum Beispiel jetzt einer 20 gute Ideen bringt für die Anderen und die die alle benutzen, dann sieht Pepper das nicht. Aber wenn du jetzt ein Team mit 20 Mitgliedern hast zum Beispiel und einer bringt zum Beispiel jetzt für die anderen 19 die guten Ideen und die bauen die dann eigentlich nur noch aus und finalisieren sie und deine eigene Idee ist gerade Scheiße und dein Thema gerade nicht läuft, dann sieht halt Pepper eigentlich am Schluss nur, dass du eine Scheiße abgeliefert hast und die anderen besser waren. Obwohl du eigentlich für die anderen 19 der Mainschreiber warst. (unv. #00:08:50#) über Teamdynamik und wie man das Ganze findet, was man halt nebenbei einfach noch so macht, das kann von Pepper nie wertgeschätzt werden. #00:08:57#

**Interviewer:**

Okay. Also meinst du, er kann nicht wirklich nachvollziehen, zum Beispiel woher die Ideen kommen? #00:09:01#

**Interviewpartner:**

Genau. Woher die Ideen kommen, was du auch nebenbei neben deinem Job noch machst. Wenn jetzt irgendwer von einer anderen Abteilung kommt und sagt. „Hei du, hast du da noch eine Idee?“ oder so und du das halt noch schnell machst, deine eigene Arbeit liegen lässt, dann zeigt dir das Pepper nicht. #00:09:15#

**Interviewer:**

Okay. Kannst du Pepper als Führungskraft vertrauen? #00:09:20#

**Interviewpartner:**

Als Führungskraft vertrauen? Boah, das ist ja jetzt vage formuliert. Als Führungskraft vertrauen? Wie vertrauen? #00:09:33#

**Interviewer:**

Wenn er jetzt dein Chef ist: Normalerweise vertraut man ja gewissermaßen seinem Chef. Würdest du jetzt auch Pepper vertrauen? #00:09:43#

**Interviewpartner:**

Ja. #00:09:47#

**Interviewer:**

Inwiefern? Also nur, dass er die richtigen Entscheidungen trifft oder / #00:09:53#

**Interviewpartner:**

Also so meinst du jetzt vertrauen? Ich habe jetzt gemeint, vertrauen, wenn ich irgendwas sage, dass er jetzt nicht zu meinem Mitarbeiter geht und „Hej, (unv. #00:09:59#). Aber du kannst es besser.“ #00:10:01#

**Interviewer:**

Okay, ist auch gut. #00:10:02#

**Interviewpartner:**

Richtige Entscheidungen treffen? Vielleicht andere Bereiche, vielleicht nicht im Marketing, wie sich schon erwähnt habe. Allgemein vertrauen würde ich jetzt schon sagen, dass er sozusagen, wenn ich jetzt was sage, dass es meine Idee war, dass er nicht zu wem anders geht und dem das dann gibt als Aufgabe. #00:10:25#

**Interviewer:**

Okay. Also dass er deine Ideen oder deine / was du halt zu ihm sagst, auch vertraulich behandelt? #00:10:32#

**Interviewpartner:**

Genau, ja. #00:10:34#

**Interviewer:**

Kannst du auch vertrauen, okay. Welche Verhaltensweisen waren dir sympathisch? #00:10:40#

**Interviewpartner:**

Körperhaltung, wie schon erwähnt. Also dass er seinen ganzen Körper benutzt hat, dass es ein bisschen menschlicher gewirkt hat. (Wobei? #00:10:47#) es mir eigentlich egal ist, aber es ist einfach ein bisschen netter. Dass sie das relativ erklärt hat auch für Dummies. Was war sonst noch sympathisch? Das war es eigentlich. #00:11:04#

**Interviewer:**

Okay, also die Gestik und wie er es erklärt hat? #00:11:07#

**Interviewpartner:**

Ja. #00:11:08#

**Interviewer:**

Okay. Und was hat dich an seinem Verhalten gestört? #00:11:11#

**Interviewpartner:**

Ja, hin und wieder war er halt ein bisschen fordernd. Das war wahrscheinlich absichtlich. #00:11:17#

**Interviewer:**

Was meinst du mit fordernd? Also wie er / #00:11:19#

**Interviewpartner:**

Ja, „Schreib jeden Schritt auf, du darfst keine Fehler machen“ und so weiter. Also das ist jetzt nicht unbedingt das Beste für das Arbeitsklima. Das war störend. Und die Sprache war störend. #00:11:32#

**Interviewer:**

Okay. Wie würde es dir gehen, wenn Pepper dein Chef wäre? #00:11:37#

**Interviewpartner:**

In der Firma jetzt? #00:11:42#

**Interviewer:**

Ja, oder woanders. Allgemein eigentlich. #00:11:45#

**Interviewpartner:**

Erst mal würde ich wahrscheinlich überfordert sein, weil es eine völlig neue Situation ist für jeden. Und wahrscheinlich wäre ich ehrlich gesagt ein bisschen angepisst, weil ich jetzt nicht sehe, wie Pepper mir helfen kann zum Beispiel in meiner persönlichen Karriereentwicklung, wie er mir helfen kann. Weil ich sehe vielleicht Pepper jetzt als Chef für die Businessentscheidungen für die ganze Firma vielleicht nicht schlecht. Aber jetzt als Mentor oder so für mich selber habe ich jetzt Pepper noch gar nicht gesehen, dass jetzt die irgendwie da sozusagen mir sagt: „Hei, mit dem kannst du vielleicht arbeiten. Das kannst du arbeiten.“ Oder zum Beispiel auch so Sachen, sagen: „Ja, wenn es dir jetzt keinen Bock mehr bei uns macht, ich kenne da noch wen anders in einer anderen Firma. Vielleicht kannst du bei dem anfangen“ und so weiter, solche Sachen sind bei Pepper nicht vorhanden, weil die nur auf das Wohl der Firma und nicht auf das / #00:12:40#

**Interviewer:**

Okay. Also persönliche Entwicklung? #00:12:41#

**Interviewpartner:**

Genau. Persönliche Entwicklung ist halt, was eigentlich das Wichtigste ist, vor allem für viele Mitarbeiter in der heutigen Zeit, ist bei Pepper nicht gegeben. #00:12:49#

**Interviewer:**

Okay. Was meinst du, was hingegen gut funktionieren würde? #00:12:56#

**Interviewpartner:**

Wenn es weiterentwickelt ist, die Entscheidungsfindung auf jeden Fall, weil halt einfach eine Maschine bei vielen Sachen einfach besser Entscheidungen treffen kann rein von der CPU-Auslastung sozusagen. Dann das ganze Organisieren und so weiter würde natürlich viel besser laufen, weil das / du bräuchtet halt eigentlich keine Assistenz mehr in dem Sinne, weil das kann alles die Pepper selber machen: Teammeetings und blablabla und Projektplan und die ganzen anderen Sachen, die einfach halt bei einem Projekt anfallen, wo ich eigentlich irgendeinen depperten Werkstudenten oder Praktikanten abstellen muss, dass der das alles organisiert und monitort. Das kann Pepper eigentlich auch alles direkt daneben machen. Was auch gut ist, dass es halt einfach eine Arbeitskraft weniger ist, dir Kosten erspart hast. Vor allem, wenn es jetzt ein Chef ist, ist es halt einfach ein relativ großes Gehalt, das du dir sogar sparen kannst. Was ist sonst noch positiv? Das war es jetzt eigentlich schon. #00:13:54#

**Interviewer:**

Wenn er sehr strenges Feedback gibt: Wie würdest du damit umgehen? #00:14:01#

**Interviewpartner:**

Ich würde es zu Herzen nehmen und versuchen, mich zu verbessern. #00:14:07#

**Interviewer:**

Okay. Also dann auch akzeptieren und dran arbeiten? #00:14:09#

**Interviewpartner:**

Feedback ist immer relativ gut eigentlich, wenn es jetzt nicht persönlich ist, sondern halt nur auf die Arbeit bezogen ist, dann ja. #00:14:15#

**Interviewer:**

Okay. Und meinst du, er würde dann auch / #00:14:18#

**Interviewpartner:**

(unv. #00:14:18#) er bezieht es nur auf die Arbeit, weil es ein Computer ist. #00:14:21#

**Interviewer:**

Okay. Wie gut, meinst du, kann Pepper für die Zukunft seines Teams Pläne erstellen und Ziele setzen? #00:14:29#

**Interviewpartner:**

Pläne erstellen und Ziele setzen? (überlegt etwas) Das kommt drauf an, wie viel Inputvariablen Pepper zur Verfügung gestellt werden. Also es sind halt einfach auch viele Sachen wie Auslastung des Teams mit anderen Themen, die jetzt vielleicht nicht gerade am Schirm sind. Auch kompanieweite Ziele, die gesetzt werden. Einfach schnelle, akute Maßnahmen, die jetzt gerade getroffen werden müssen und solche Sachen. Wenn die Pepper zur Verfügung gestellt werden, dann glaube ich schon, relativ gut. Wenn aber die halt fehlen, dann relativ schlecht. #00:15:13#

**Interviewer:**

Okay. Also machst du es stark davon abhängig, wie viele Informationen man ihm gibt? #00:15:16#

**Interviewpartner:**

Ja. #00:15:18#

**Interviewer:**

Okay. Was würdest du vermuten: Wie erfolgreich wäre dein Team, das von Pepper geführt wird? #00:15:24#

**Interviewpartner:**

Erfolgreich in welchem Sinne? #00:15:27#

**Interviewer:**

Traditionell, im Sinne, also auch zum Beispiel wenn du zwei Teams nimmst, einmal von Pepper geführt, einmal von einem Menschen: Wer würde da besser abschneiden? #00:15:40#

**Interviewpartner:**

Das kommt auf den Bereich meines Erachtens an. Also wie gesagt, ich glaube, im Financebereich, Business Analytics, so was würde, glaube ich, das Team von Pepper ziemlich gut abschneiden. Aber in einem Team wie zum Beispiel Marketing oder HR, wo man vielleicht nicht alles quantifizieren kann, wo man sozusagen / wo man einfach ein bisschen – schwierig zu beschreiben -, aber / #00:16:10#

**Interviewer:**

Die weichen Faktoren? #00:16:12#

**Interviewpartner:**

Die weichen Faktoren, seelische Faktoren mit beeinflussen muss, dann glaube ich, ist das andere Team besser. #00:16:18#

**Interviewer:**

Okay. Dann sind wir schon durch.
