# Supplementary material for: Robot leadership–Investigating human perceptions and reactions towards social robots showing leadership behaviors
Source: PLoS One. 2023 Feb 16;18(2):e0281786. doi: 10.1371/journal.pone.0281786 (PMC9934409; doi:10.1371/journal.pone.0281786)
Supplement: S1 Dataset — (ZIP) [file pone.0281786.s002.zip › Interview_Participant6.doc]

**10 Interview**

**Interviewer:**

Was für Gedanken hast du dir gemacht, während Pepper mit dir geredet hat? #00:00:08#

**Interviewpartner:**

Was ich mir für Gedanken gemacht habe? Ich fand das ziemlich impressiv, also ziemlich impressive. Ich habe eigentlich zwei Hauptgedanken gehabt. Der erste Hauptgedanke war, dass man sie sehr schlecht verstanden hat. Das fand ich sehr schlecht. Es war sehr mechanisch, die Sprache. Deswegen hatte ich dadurch Probleme. Zweiter Gedanke war, dass es ziemlich geil ist, eigentlich auch zusammen mit diesem open gardener, dass man / oder wie auch immer dieses Startup heißt, dass eigentlich an sich die Idee schon ziemlich cool ist. Aber wenn man das dann auch verbindet zum Beispiel mit einem Roboter, der das dann macht, das im Endeffekt so was wie die Zukunft ist. Dass heute / Ich weiß nicht, ob heute der Markt für so was schon so groß ist. Aber später dann, wenn dann wirklich die Roboter gießen sollen, dann ist das, glaube ich, dann braucht man so was. Und dann / #00:01:11#

**Interviewerin:**

Du meinst quasi, wenn Pepper nicht nur jetzt (unv. #00:01:12#) irgendwie ist, sondern dass er tatsächlich auch das macht, was da das Produkt ist. #00:01:16#

**Interviewpartner:**

Genau, genau. Das war so meine Idee. Das waren die zwei Hauptideen, die mir gekommen sind. Ansonsten, ich fand das ziemlich beeindruckend, der Roboter, auch wie er sich bewegt hat. Ein bisschen künstlich, aber / #00:01:27#

**Interviewer:**

(unv. #00:01:28#), okay. Wie hat es sich jetzt für dich angefühlt, dass Pepper mit dir so geredet hat? #00:01:36#

**Interviewpartner:**

Sehr künstlich. Das war jetzt nicht / eher fast ein bisschen lächerlich. #00:01:46#

**Interviewer:**

Okay. Wieso lächerlich? Weil / #00:01:50#

**Interviewpartner:**

Ja, weil es so mechanisch geklungen hat und in keinster Weise irgendwie / Ja, ich will nicht sagen, nicht menschlich. Und ich meine, letztlich geht es ja darum, ob sie Führungskräfte / oder als Führungskraft taugen würde und da ist es eben so, finde ich, dass man das vielleicht doch machen würde, weil es Spaß macht irgendwie so am Anfang. Aber auf lange Zeit, glaube ich nicht. Fände ich es einfach lächerlich. #00:02:22#

**Interviewer:**

Okay. Für einen Roboter zu arbeiten? #00:02:25#

**Interviewpartner:**

Ja. #00:02:26#

**Interviewer:**

Okay. Was hast du gedacht, als Pepper „We do not tolerate any mistakes“ gesagt hat? #00:02:32#

**Interviewpartner:**

Da habe ich gar nichts gedacht (lacht). Wobei, ich fand, es war so ein bisschen, auch da ein bisschen künstlich. Es ist nicht so irgendwie / keine Ahnung. Es war nicht so / Ich habe es nicht als Strafe oder nicht als Bedrohung gesehen, sondern eher, ja, einfach / so einfach. #00:03:02#

**Interviewer:**

Nicht wirklich ernst genommen? #00:03:03#

**Interviewpartner:**

Nein. #00:03:05#

**Interviewer:**

Okay. Was denkst du, wie Pepper reagieren würde, wenn du einen Fehler machst? #00:03:12#

**Interviewpartner:**

(überlegt länger) Ich denke, er/sie/es würde eine moralische Rede halten. Das heißt, sozusagen nicht ermahnen oder sozusagen wirklich bestrafen, dass ich einen Fehler gemacht habe. (unv. #00:03:45#) dass das nicht okay ist. #00:03:48#

**Interviewer:**

Okay. Also schon ein bisschen so nachhaken, dass du einen Fehler gemacht hast? #00:03:54#

**Interviewpartner:**

Ja. Er würde darauf rumhacken und er würde darauf bestehen, dass man das in Zukunft vielleicht nicht mehr macht. #00:04:02#

**Interviewer:**

Okay. Pepper hat ja gesagt: „You should document what you do so that I am able to follow your steps and identify mistakes. This will allow me, go give you exact feedback on your work.” Hast du dich jetzt bewusst an seine Vorgaben gehalten? #00:04:21#

**Interviewpartner:**

Tatsächlich, das war, warum ich nachgefragt hatte, ob ich das auch erklären soll, was ich mache. Ja, also das habe ich dann schon, das hätte ich schon ernst genommen. Habe ich mich dran gehalten beim Aufschreiben der Sachen? Eigentlich nicht. Aber das lag dann an euch, weil ihr mir gesagt habt, ich soll (unv. #00:04:40#) Sonst hätte ich das gemacht. #00:04:43#

**Interviewer:**

Und was meinst du, wieso hast du ihn dabei ernst genommen? #00:04:47#

**Interviewpartner:**

Warum habe ich ihn dabei ernst genommen? Weil das einfach eine Beschreibung war, das war für mich so irgendwie die Taskbeschreibung. Im Endeffekt sozusagen als: Okay, das habe ich zu machen. Und die Bedrohung oder die Drohung, Androhung vorher, das war schon / das hat einfach, man bräuchte da Konsequenzen, die man so vielleicht beim Roboter nicht sieht. #00:05:17#

**Interviewer:**

Okay. Wenn Pepper ein sehr strenges Feedback dir gibt, wie würdest du damit umgehen? #00:05:26#

**Interviewpartner:**

Ich werde es wahrscheinlich (unv. #00:05:30#) würde aber wahrscheinlich im Unterbewussten das trotzdem (unv. #00:05:36#), also das heißt, ich würde beim nächsten Mal wahrscheinlich mich ein bisschen mehr anstrengen. Nicht so viel, wie wenn ein Mensch mir geben würde, aber durchaus schon ein bisschen. #00:05:46#

**Interviewer:**

Okay. Also auf den ersten Blick erst mal so ein bisschen amüsant finden, aber dann trotzdem berücksichtigen? #00:05:53#

**Interviewpartner:**

Ja. #00:05:54#

**Interviewer:**

Wie war dein Gesamteindruck von Pepper? #00:05:57#

**Interviewpartner:**

Amüsant, beeindruckt, aber nicht / ja, mehr eine Spielerei als was Ernstzunehmendes. #00:06:15#

**Interviewer:**

Okay. Wieso eine Spielerei? #00:06:21#

**Interviewpartner:**

Wieso eine Spielerei: Also die Bewegungen waren halt extrem, also sehr, sehr ruckartig. Deswegen hat es mir den Eindruck gemacht, das war noch ein sehr früher Entwicklungsstand für einen Roboter. Gleichzeitig auch irgendwie so versuchen, zu imitieren, aber halt da im Endeffekt, ja, wie ein schlechter Schauspieler. #00:06:48#

**Interviewer:**

Okay, ja. #00:06:49#

**Interviewpartner:**

Und das dann eher amüsant und nicht ernstnehmend. Genau. #00:06:55#

**Interviewer:**

Inwiefern hast du dich von Pepper motiviert gefühlt? #00:06:58#

**Interviewpartner:**

(lacht kurz auf). Vielleicht auf einer Skala von 0 bis 10 maximal eine 2. #00:07:06#

**Interviewer:**

Okay. Und wenn du dir jetzt vorstellst, Pepper wäre dein Chef, würdest du dich von ihm motiviert fühlen? #00:07:14#

**Interviewpartner:**

(lacht etwas). Jetzt glaube ich, machen wir das mal lieber aus, (unv. #00:07:22#) Spaß beiseite. Dann würde ich mich / nein. Nein. #00:07:31#

**Interviewer:**

Weißt du, wieso? #00:07:33#

**Interviewpartner:**

Eben, weil es nicht, wie sagt man, nicht – wie heißt das Wort? – nicht: enthusiastisch. Sondern nicht / Ja, vielleicht auch nicht enthusiastisch, halt einfach auch nicht / Wie nennt man das, wenn man / nicht glaubhaft. Wie heißt das Wort? #00:07:55#

**Interviewerin:**

Glaubwürdig. #00:07:56#

**Interviewpartner:**

Ja, aber auch da gibt es / nicht so menschlich, also irgendwie / #00:08:01#

**Interviewer:**

Authentisch. #00:08:01#

**Interviewpartner:**

Authentisch, genau, das wollte ich sagen: nicht authentisch. #00:08:05#

**Interviewer:**

Okay. Fühlst du dich von Pepper wertgeschätzt? #00:08:10#

**Interviewpartner:**

(lacht auf). Nein. Aber weil, ich glaube, das gibt es nicht. Also es gibt da keine Art der Wertschätzung. Das heißt, es ist nicht existent. Ich meine, um wertgeschätzt / um dass ich mich wertgeschätzt fühle, dazu muss ich das Gefühl haben, dass der Gegenüber auf meine Aktionen reagiert. Das würde sie tun oder er, es, würde es tun. Aber in einer menschlichen Art und Weise, ich sag jetzt mal, auf einer Gefühlsebene Wertschätzung. Und das würde ich nicht bekommen. Oder das Gefühl habe ich nicht, dass ich das bekommen würde. #00:08:52#

**Interviewer:**

Okay. Also dass Pepper irgendwie Gefühle entwickeln kann oder dir zeigen kann? #00:08:59#

**Interviewpartner:**

Ja. Oder dass ich halt / Es ist ja egal, ich meine, letztlich, ob es da Gefühle gibt oder nicht. Das sei mal dahingestellt. Aber letztlich, dass ich das Gefühl habe, dass sie/er/es Gefühle für mich hat. #00:09:11#

**Interviewer:**

Okay. Also es würde nicht so rüberkommen, egal, ob er jetzt tatsächlich Gefühle hat oder nicht? Okay. #00:09:16#

**Interviewpartner:**

Was nicht heißt, dass es nicht die Möglichkeit gibt bei einem Roboter. Also das stelle ich absolut nicht in Frage, dass es das mal geben wird. Und dann würde ich wahrscheinlich schon eher wertschätzen. #00:09:26#

**Interviewer:**

Denkst du, Pepper ist fair zu seinen Teammitgliedern? #00:09:30#

**Interviewpartner:**

Ich glaube, das Konzept gibt es nicht in dem Sinne. Also Fairness in dem Sinne. Ja, würde man es rational betrachten, ist es 100 % fair, weil er letztendlich jeden gleich behandelt. Also da 100 % Fairness. Aber im Sinne von menschlicher Fairness, das heißt, dass man durchaus auch die Umstände berücksichtigt und so was, das würde es nicht geben. Ist die Frage, ob das dann noch fair ist. #00:10:03#

**Interviewer:**

Also würde er objektiv fair sein, ganz nüchtern. Aber halt nicht / #00:10:08#

**Interviewpartner:**

(unv. #00:10:07#) fair halt, ja. Sondern ja. #00:10:12#

**Interviewer:**

Kannst du Pepper als Führungskraft vertrauen? #00:10:15#

**Interviewpartner:**

Dazu würde ich gerne die Programmierer zuerst kennenlernen. Kann ich vertrauen? Nein. Was (unv. #00:10:33#) vertrauen in dem Sinne? Es ist, glaube ich, dann auch wieder so eine Definitionssache, so wie ich es definiere halt. #00:10:39#

**Interviewer:**

Okay. Und wie? #00:10:42#

**Interviewpartner:**

Weil Vertrauen auch wieder im Endeffekt Feedback benötigt im Sinne von menschlichem Feedback oder Gefühlsfeedback. Das ist für mich bei Vertrauen sehr wichtig, dass ich einschätzen kann. Und das, glaube ich, könnte ich nicht bei einem Roboter. #00:10:58#

**Interviewer:**

Welche seiner Verhaltensweisen waren dir sympathisch? #00:11:02#

**Interviewpartner:**

Das Blinken. Der hat es mit den Augen so gemacht, das fand ich ziemlich cool. Und die Hände fand ich richtig cool, also dass sie die Finger auch so bewegt hat. Das fand ich sehr cool. #00:11:13#

**Interviewer:**

Und wieso du das jetzt sympathisch findest oder / #00:11:18#

**Interviewpartner:**

Es machte einen sehr menschlichen Eindruck. #00:11:20#

**Interviewer:**

Okay. Also menschlich. Was hat dich an seinem Verhalten gestört? #00:11:25#

**Interviewpartner:**

Die ruckartigen Bewegungen des Körpers, des Oberkörpers. Dann hat er die ganze Zeit dich fokussiert und nicht mich (lacht etwas), mit den Augen. Das war ein bisschen / weil ich ja letztlich der Empfänger eigentlich der Aufgabe war. Und ansonsten? Ja, das waren eigentlich die beiden unsympathischen Sachen. #00:11:53#

**Interviewer:**

Wie würde es dir gehen, wenn Pepper jetzt dein Chef wäre? Was würde besonders gut oder nicht so gut funktionieren? #00:12:00#

**Interviewpartner:**

Ich wüsste ganz genau, was ich machen müsste. Ich würde aber auch sehr wenige Freiheiten haben wahrscheinlich, so würde ich es einschätzen. Das heißt, ich habe keine / ich kann mich selber nicht entfalten. Und ja, es wäre wahrscheinlich ein sehr trister Alltag. #00:12:26#

**Interviewer:**

Okay. Also meinst du, er würde ganz streng vorgeben, was man machen soll und wie? #00:12:30#

**Interviewpartner:**

Ja, sehr, man würde sagen: sehr korinthisch wahrscheinlich. Also sehr, sehr kleinteilig. Wie so ein kleiner Korinthenkacker. #00:12:41#

**Interviewer:**

(lacht). Okay. Wie gut, meinst du, kann Pepper für die Zukunft seines Teams Pläne erstellen und Ziele setzen? #00:12:51#

**Interviewpartner:**

Ich nehme an, das kommt ganz stark auf die Aufgabe drauf an. Wenn es rein mechanische Aufgaben sind oder Aufgaben, die in einer strengen Reihenfolge folgen müssen, dann / #00:13:08#

**Interviewerin:**

Du kannst gerne genau an die Aufgaben, von denen er gesprochen hat, denken. Sozusagen erst Marketingmanager und was ist sozusagen seine Aufgabe, sich zu überlegen zusammen mit seinem Team, wie man das jetzt macht. #00:13:21#

**Interviewpartner:**

Mit Marketing. Gar nicht. Da würde ich sagen, könnte er nicht machen. Weil es ein sehr, sehr kreativer Prozess ist und ich denke, dass das letztlich Extrapolierung ist von Daten, von Gedankengängen. Und das kann er nicht. Würde ich nicht einschätzen. #00:13:47#

**Interviewer:**

Okay. Also kann er nicht planen, Ziele setzen auch nicht? #00:13:50#

**Interviewpartner:**

Ziele setzen schon, aber Ziele: Okay, wir wollen den und den Umsatz machen, wir wollen den und diesen Impact generieren im Sinne von Marketing; wir wollen den und diese Anzahl von Konsumenten oder Kunden erreichen. Ja, das schon. Aber planen im Sinne: Wie kommen wir dahin? Nein. #00:14:16#

**Interviewer:**

Aber könnte Ziele setzen? #00:14:18#

**Interviewpartner:**

Ja. Wie gesagt, sofern es wirklich sozusagen Ziele sind, die sich mit Zahlen festhalten lassen. Qualitative Ziele eher nicht. #00:14:28#

**Interviewer:**

Was würdest du vermuten: Wie erfolgreich wäre ein Team, das von Pepper geführt wird? #00:14:34#

**Interviewpartner:**

Wie: erfolgreich? Bezogen auf die Aufgabe nicht sehr erfolgreich. In Bezug auf andere Aufgaben durchaus erfolgreich. #00:14:47#

**Interviewer:**

Okay. Was für Aufgaben? #00:14:48#

**Interviewpartner:**

Was kam mir jetzt als erstes in den Kopf? So was wie Amazon irgendwie, so Verteilerzentrum von Amazon zum Beispiel, da würde ich das als durchaus erfolgreich sehen. Forschung sicher nicht erfolgreich, so wissenschaftliche Arbeit nicht erfolgreich. Aber so was zum Beispiel wie bei Pizza-Lieferservice oder so, wo eben dann eingeteilt wird, wer was zu machen hat, bis wann zu machen ist und so: da ja. Genau, solche Aufgaben. #00:15:23#

**Interviewer:**

Also eher so nüchterne Bereiche? #00:15:27#

**Interviewpartner:**

Wo man einfach einen konkreten Prozess angeben kann und dann, wo Pepper eben sagt: „Hei, das sind die Prozessziele, das sind die Prozessschritte und der und der macht das.“ Da würde ich, glaube ich, (unv. #00:15:39#) führen können. #00:15:41#

**Interviewer:**

Das waren jetzt schon alle Fragen. #00:15:45#

**Interviewerin:**

Hast du noch was hinzuzufügen, was wir nicht gefragt haben oder was dir irgendwo / #00:15:48#

**Interviewpartner:**

(überlegt länger) Du hattest mir erzählt, du würdest eine Führungskraft damit / so was in Richtung Respekt oder so was, würde ich vielleicht fragen. #00:16:04#

**Interviewerin:**

Was würdest du antworten? #00:16:05#

**Interviewpartner:**

Wie respektiert er? Was würde ich antworten? Kein Respekt, ich habe persönlich keinen Respekt. Ja, doch, vielleicht so ein bisschen, so ein bisschen. Aber auf eben / weil er durch so eine Nüchternheit und so eine / ein bisschen Respekt erarbeitet hat. Er hat sich ja nicht Respekt erarbeitet.

(Lachen allseits)

Doch, doch, doch, also würde ich schon sagen. Danach wollte ich fragen. Das ist letztlich vielleicht auch etwas, was für Führungskräfte sehr wichtig ist. Also ich meine, da sind eher die Experten (unv. #00:16:44#) einfach das, was ich persönlich empfinde als wichtig. Vielleicht noch in die Richtung: Wie würde, und das ist, glaube ich, für eine Führungskraft sehr, sehr wichtig, er nicht sozusagen mit Problemen umgehen, sondern mit Situationen, die vielleicht nicht vorhersehbar sind? Also sozusagen, wie würde man mit unvorhergesehenen Situationen umgehen und kann das Pepper? Da würde ich antworten: nein. Aber ich denke, es ist eine sehr wichtig Führungskrafteigenschaft, die mit fünfmal gesehenen, zufälligen Sachen / drauf einzugehen und sei es zum Beispiel, dass ein Mitarbeiter krank wird. Ich nehme an, das würde halt zum Beispiel Pepper als Fehler oder als Fehlerfolg oder wie auch immer einordnen. Wohingegen eine gute Führungskraft das halt eben mit berücksichtigt, Umstände eben mit berücksichtigt und das nicht als Fehler, sondern als, ja, wie soll man sagen, Zufall (unv. #00:17:52#) Umstände einstufen würde. Genau. #00:17:56#

**Interviewerin:**

Cool. Vielen Dank dir. #00:17:58#

**Interviewer:**

Noch ein paar persönliche Fragen: Wie alt bist du? #00:18:01#

**Interviewpartner:**

Jetzt muss ich selber überlegen. 29. #00:18:05#

**Interviewer:**

Was bist du? Also Student? #00:18:10#

**Interviewpartner:**

Promovierter Mitarbeiter. #00:18:12#

**Interviewer:**

Studiengang? Also eher, was für einen / #00:18:20#

**Interviewpartner:**

Mathe habe ich gemacht. (unv. #00:18:23#)

**Interviewer:**

Hattest du schon mal Erfahrung mit Robotern, AI, solche Sachen? #00:18:29#

**Interviewpartner:**

Ja. Roboter, ja. So mit Kuka und so habe ich zum Beispiel gesehen oder bei BMW eben auch zum Beispiel in den Werken die eingesetzten gesehen. Also aktiv jetzt nicht damit gearbeitet. AI: Ich mache in meiner Freizeit viel Maschinenlearning. Also viel nicht, aber ich mache Maschinenlearning. #00:18:51#

**Interviewer:**

Okay, perfekt.
