# Supplementary material for: Robot leadership–Investigating human perceptions and reactions towards social robots showing leadership behaviors
Source: PLoS One. 2023 Feb 16;18(2):e0281786. doi: 10.1371/journal.pone.0281786 (PMC9934409; doi:10.1371/journal.pone.0281786)
Supplement: S1 Dataset — (ZIP) [file pone.0281786.s002.zip › Interview_Participant7.doc]

**13 Interview**

**Interviewer:**

Also wenn du dir jetzt so als Prämisse vorstellst, du arbeitest für Pepper oder in seinem Team halt. Was für Gedanken sind dir durch den Kopf gegangen, während Pepper mit dir geredet hat? #00:00:16#

**Interviewpartnerin:**

Also es ist eine sehr schöne Idee, mit Pepper (unv. #00:00:24#) zu betreiben, weil es mal was anderes ist. Es war ganz nett, dass er sich auch dazu bewegt hat und dass er dich so genau angeschaut hat sozusagen. Ich fand teilweise es ein bisschen unverständlich, was er gesagt hat; das ist mir aufgefallen. Ja. Ja. #00:00:42#

**Interviewer:**

Und wie hat es sich so angefühlt, von Pepper jetzt angesprochen zu werden? #00:00:49#

**Interviewpartnerin:**

Ja, ein bisschen komisch, weil es natürlich kein Mensch ist. Aber sonst ganz normal. #00:00:57#

**Interviewer:**

Und inwieweit komisch? #00:01:00#

**Interviewpartnerin:**

Na ja, es ist halt komisch, mit einem Ding zu sprechen und das war halt ein bisschen ungewohnt. Auch dass er so wie ein Mensch agiert, sag ich mal, und dich auch so anschaut, sich bewegt. Also das war so ein bisschen ungewohnt. Aber ja. #00:01:15#

**Interviewer:**

Was hast du gedacht, als Pepper gesagt hat: „We do not tolerate any mistakes.“ #00:01:23#

**Interviewpartnerin:**

Da dachte ich mir, dass es nicht funktionieren kann, weil es ist ja ganz menschlich. Okay, er ist jetzt kein Mensch, aber generell ist es normal, dass Fehler passieren und das Wichtige ist ja, dass man aus Fehlern lernt. Und es ist vielleicht auch wichtig, dass mal nicht alles richtig läuft, damit man merkt, wo man sozusagen sich noch verbessern kann und was man ändern sollte und was schon gut ist. #00:01:47#

**Interviewer:**

Okay. Also dass er keine Fehler toleriert, wie findest du das? #00:01:51#

**Interviewpartnerin:**

Ich finde das schwierig persönlich, weil vielleicht dann auch viele Leute abgeschreckt sind, für ihn zu arbeiten, weil sie sagen: Fehler sind menschlich. Sag ich mal. Und ja, es ist auch unrealistisch. #00:02:06#

**Interviewer:**

Was denkst du, wie Pepper reagieren würde, wenn du jetzt einen Fehler machst? #00:02:14#

**Interviewpartnerin:**

Vielleicht erscheint dann so ein Warnsignal (lacht) oder so. #00:02:21#

**Interviewer:**

Okay. #00:02:23#

**Interviewpartnerin:**

Oder er erklärt mir, wie man es besser machen könnte beziehungsweise weist mich, sobald er merkt, dass ich einen Fehler mache, drauf hin und versucht den abzuwenden. So was vielleicht. Also wenn er das kann. #00:02:35#

**Interviewer:**

Direkt, er würde direkt eingreifen? #00:02:38#

**Interviewpartnerin:**

Genau. #00:02:39#

**Interviewer:**

Damit es halt nicht zu einem größeren Fehler kommen könnte. #00:02:42#

**Interviewpartnerin:**

Genau. #00:02:43#

**Interviewer:**

Pepper hat auch gesagt: „You should document what you do so that I am able to follow your steps and identify mistakes. This will allow me, go give you exact feedback on your work.” Hast du dich bewusst an diese Vorgabe gehalten?

**Interviewpartnerin:**

Wie meinst du? #00:03:02#

**Interviewer:**

Er hat ja gemeint, man soll seine Arbeit, also die Arbeitsschritte dokumentieren und halt ziemlich genau arbeiten. Hast du dich dran gehalten? #00:03:13#

**Interviewpartnerin:**

Jetzt gerade? #00:03:15#

**Interviewer:**

Ja. #00:03:16#

**Interviewpartnerin:**

Ja, eigentlich schon. #00:03:20#

**Interviewer:**

Bewusst oder einfach nur / #00:03:21#

**Interviewpartnerin:**

Unbewusst, aber / #00:03:24#

**Interviewer:**

Okay. Aber könnte auch einfach an der Aufgabe liegen? #00:03:28#

**Interviewpartnerin:**

Genau. #00:03:31#

**Interviewer:**

Okay. Wie war so dein Gesamteindruck von Pepper? #00:03:35#

**Interviewpartnerin:**

Ja, eigentlich ganz gut. Also es war eine, ja, schöne Erfahrung, mit einem Roboter zu sprechen. Und ich fand auch die Idee mit dem IPad ganz schön, dass man zusätzlich auch noch veranschaulicht bekommen hat, was er so erklärt hat. Und dass man auch schon so gesehen hat, wie das Produkt letztendlich aussieht und wie es funktioniert. Und es war sehr schön bildlich dargestellt. Also ich fand es eine gute Idee. #00:04:04#

**Interviewer:**

Okay. Also durchgängig positiv? #00:04:06#

**Interviewpartnerin:**

Genau. #00:04:07#

**Interviewer:**

Inwiefern hast du dich von Pepper motiviert gefühlt? #00:04:11#

**Interviewpartnerin:**

Also ich würde sagen, eigentlich normal. Also ob es jetzt ein Mensch gewesen wäre oder Pepper, hätte keine Unterschiede gemacht, denke ich. #00:04:24#

**Interviewer:**

Okay. Und wenn du dir jetzt vorstellst, du arbeitest für ihn in seinem Team: Würdest du dich von ihm motiviert fühlen? #00:04:33#

**Interviewpartnerin:**

Ja, also es kommt drauf an, was er zu mir sagt. Also wenn er motivierend wäre, dann ja. Aber ich glaube, es gäbe auch ein Problem sozusagen, dass er so die Autoritätsperson zwischen uns beiden wäre. #00:04:47#

**Interviewer:**

Wieso wäre das ein Problem? #00:04:49#

**Interviewpartnerin:**

Ja, weil er eben ein Ding ist und ich finde, Dinge stehen unter Menschen und dann ist es eben schwierig, sich von ihm was sagen zu lassen. #00:04:58#

**Interviewer:**

Und wenn er dir jetzt Anweisungen gibt, wie wäre es für dich? #00:05:02#

**Interviewpartnerin:**

Na ja, im Endeffekt wäre es nicht so schlimm, weil dann würde ich halt meine Aufgaben quasi von ihm bekommen. Aber ich würde ihn nicht als gleichwertige Person akzeptieren, sag ich mal. Also so / Weißt du, was ich meine? #00:05:16#

**Interviewer:**

Okay, also Arbeitsanweisungen schon von ihm akzeptieren, aber irgendwie nicht / #00:05:23#

**Interviewpartnerin:**

Nicht so wertschätzen als Persönlichkeit. #00:05:27#

**Interviewer:**

Okay. Fühlst du dich von Pepper wertgeschätzt? #00:05:32#

**Interviewpartnerin:**

Na ja, dadurch, dass er eben gesagt hat, er akzeptiert keine Fehler und so. Ja, und wir noch nicht so viel Kontakt haben, kann ich es noch nicht so beurteilen. Aber ich denke, wenn er dann vielleicht auch Feedback zu meiner Arbeit dann gibt und er vielleicht auch mal was Nettes sagt oder konstruktive Kritik äußert, dann, ja, schon. #00:05:56#

**Interviewer:**

Also du kannst dir auch gut vorstellen, dass er dann auch dich wertschätzt? #00:06:03#

**Interviewpartnerin:**

Ja, schon. #00:06:04#

**Interviewer:**

Denkst du, Pepper ist fair zu seinen Teammitgliedern? #00:06:09#

**Interviewpartnerin:**

Ich denke, dass Pepper vielleicht sehr rational handeln wird und vielleicht nicht so auf Emotionen von den Einzelnen eingehen kann. Oder wenn mal vielleicht was von seinem, ja, sag ich mal, optimalen Plan abweicht, dass er dann nicht so weiß, wie er drauf reagieren soll beziehungsweise falsch reagiert. Also das heißt, wenn irgendwas halt / Es werden ja immer so Standardsachen programmiert wahrscheinlich in ihm. Und wenn eben entweder etwas fehlt oder jemand vielleicht mal anders reagiert, als er eingespeichert hat, dass er dann eben falsch reagiert oder nicht weiß, was er machen soll oder so. Also das kann ich mir vorstellen. #00:06:50#

**Interviewer:**

Okay. Also er würde sehr rational handeln? #00:06:53#

**Interviewpartnerin:**

Ja. #00:06:54#

**Interviewer:**

Und solange alles nach Plan läuft, funktioniert auch alles gut. #00:06:58#

**Interviewpartnerin:**

Genau. Und sobald mal was abweicht, könnte es sein, dass es eben schwierig wird. #00:07:04#

**Interviewer:**

Okay. Und meinst du, er bleibt dann fair zu den Teammitgliedern oder / #00:07:13#

**Interviewpartnerin:**

Also ich denke nicht, dass er unfair wird, weil ich hoffe mal, dass es nicht programmiert wurde, dass er jemanden beschimpft oder so oder unfair wird. Also ich denke, er bleibt weiterhin sachlich, objektiv. Und ja, weniger emotional, hoffe ich mal. #00:07:30#

**Interviewer:**

Okay. Also meinst du, er bleibt objektiv, fair? #00:07:33#

**Interviewerpartnerin:**

Ja. #00:07:35#

**Interviewer:**

Hast du oder kannst du Pepper als Führungskraft vertrauen? #00:07:39#

**Interviewpartnerin:**

Vertrauen? #00:07:43#

**Interviewer:**

Ja. #00:07:43#

**Interviewpartnerin:**

Mhm (überlegend). Also ich finde das immer schwierig, weil es ist ja ein Roboter und irgend jemand hat den ja programmiert. Und dann ist es ja mehr so die Frage: Kann ich dem, der ihn programmiert hat, vertrauen und nicht Pepper direkt? #00:07:55#

**Interviewer:**

Okay. #00:07:55#

**Interviewpartnerin:**

Also es ist mehr so eine indirekte Sache, würde ich sagen, in dem Fall. #00:08:01#

**Interviewer:**

Okay. Aber ich meine, du kannst dir ja vorstellen, durch machine learning, AI, solche Sachen lernt der Roboter eigentlich auch gewissermaßen autonom. Und meinst du, es liegt aber halt immer noch daran, wie er ursprünglich programmiert wurde, ob man ihm vertrauen kann? #00:08:22#

**Interviewpartnerin:**

Na, ich denke, wenn ursprünglich sozusagen schon mal was falsch lief, dann würde ich ihm eher vielleicht nicht so vertrauen. Aber durch dieses autonome Lernen und so weiter, also da denke ich nicht, dass er sich irgendwas Falsches aneignet. Also da würde ich ihm dann weiterhin schon vertrauen. #00:08:40#

**Interviewer:**

Okay. Und wenn du ihn jetzt so als Chef auf den ersten Eindruck hättest, würdest du da vertrauen oder eher abschrecken und erst mal Distanz halten? #00:08:53#

**Interviewpartnerin:**

Also ich würde ihm nicht sofort vertrauen, auch erst mal Distanz halten, weil es eben komisch ist, dass er ein Ding ist und kein Mensch. #00:08:59#

**Interviewer:**

Und dann erst mal sehen, wie er sich verhält? #00:09:01#

**Interviewpartnerin:**

Ja, genau. Und dann eben sich ein Bild machen. #00:09:04#

**Interviewer:**

Welche Verhaltensweisen waren dir sympathisch an ihm? #00:09:07#

**Interviewpartnerin:**

Also ich fand seine helle, hohe Stimme ganz sympathisch. Dass er immer Augenkontakt gehalten hat. Dass er sich auch bewegt hat, obwohl er sehr stark gewippt hat, was mich auch so ein bisschen irritiert hat. #00:09:24#

**Interviewer:**

So nach vorne und hinten? #00:09:25#

**Interviewpartnerin:**

Ja, das war so ein bisschen komisch. Ja, aber sonst? Dass er auch so seine Hände bewegt hat, war ganz sympathisch und so versucht hat, ja, mit Gestik und Mimik seine Sprache zu unterstützen. #00:09:39#

**Interviewer:**

Okay. Und was hat dich an seinem Verhalten gestört? #00:09:42#

**Interviewpartnerin:**

Eben dieses Wippen, das war ein bisschen extrem. Dann ja, dass er / Ich weiß nicht, ob das dazugehört, aber mit dem, wie soll ich jetzt sagen? Genau, dass er eben so unverständlich teilweise gesprochen hat. Also das fand ich auch ein bisschen schwierig. Aber sonst. #00:10:07#

**Interviewer:**

Also dass ein bisschen die Stimme / #00:10:09#

**Interviewpartnerin:**

Es war schwierig, ihn akustisch zu verstehen. Also anfangs fiel es mir schwerer; als er dann angefangen hat zu reden, ging es ganz gut. Aber teilweise war es ein bisschen schwierig, fand ich. #00:10:19#

**Interviewer:**

Okay. War es dann undeutlich, wie er geredet hat? #00:10:23#

**Interviewpartnerin:**

Ich kann es gar nicht so sagen. Also ich glaube tatsächlich, dass er / es ist undeutlich rausgekommen so (unv. #00:10:28#) aus ihm. Also es war jetzt nicht, dass irgendwie ich die Wortwahl oder so nicht verstanden hätte und es war auch nicht zu leise oder so. Sondern es war so ein bisschen – weiß ich nicht. Vielleicht auch zu schnell. #00:10:39#

**Interviewer:**

Wie würde es dir jetzt gehen, wenn Pepper dein Chef wäre? #00:10:45#

**Interviewpartnerin:**

Wie es mir gehen würde? Also es wäre natürlich im ersten Moment schon ein bisschen komisch. Wie gesagt, weil er ist halt keine so richtige Autoritätsperson für mich, weil er ja ein Roboter ist. Und deswegen wäre es, glaube ich, schwierig. #00:11:06#

**Interviewer:**

Okay. Einfach nur, weil er ein Roboter ist? #00:11:09#

**Interviewpartnerin:**

Ja, und weil man es halt nicht so gewöhnt ist bis jetzt. #00:11:13#

**Interviewer:**

Okay. Aber meinst du, er könnte da irgendwie Autorität gewinnen mit der Zeit irgendwie durch Erfahrungen? #00:11:22#

**Interviewpartnerin:**

Also für mich wäre es schwierig; ich glaube nicht. Weil er ist erstens ja auch nicht so groß. Er hat irgendwie auch so eine Kinderstimme. Also er wirkt jetzt nicht so wie so ein Erwachsener, der weiß, wo es lang geht, sag ich mal. Sondern mehr so, ja, wie ein kleines Kind. Also es kann gut sein, dass er sehr kompetent ist, aber vom äußeren Erscheinungsbild wirkt er eben nicht so. #00:11:46#

**Interviewer:**

Okay. Also kommt er nicht so / #00:11:47#

**Interviewpartnerin:**

Kommt er nicht so rüber, ja. #00:11:49#

**Interviewer:**

Was meinst du, was mit ihm als Chef gut oder schlecht funktionieren würde? #00:11:54#

**Interviewpartnerin:**

Na ja, wie gesagt, so die Autoritätssache, das würde, glaube ich, vielleicht für mehrere ein Problem darstellen so. Gut laufen könnte, dass er eben objektiv handelt, so würde ich ihn jetzt einschätzen; dass er nicht wertet oder auch Personen einfach nicht mag und dann die schlechter behandelt als andere. Dass er auch vielleicht eine objektive Aufgabenverteilung hat, dass er da auch nicht so nach, ja, persönlichen Sachen entscheidet, wer jetzt welche Aufgaben bekommt. #00:12:29#

**Interviewer:**

Also nicht irgendwie jemanden bevorzugt? #00:12:31#

**Interviewpartnerin:**

Genau, dass er keinen bevorzugt oder benachteiligt. Genau so. Und ich glaube auch, dass er ein strenger Chef wäre, sag ich mal. Also er wird, glaube ich, schon relativ viel fordern und auch gutes Feedback geben, was ja auch wichtig ist. Und sozusagen versuchen, auch die Fehler zu vermeiden, bevor sie so richtig gravierend werden, was ja auch nicht schlecht ist. #00:12:57#

**Interviewer:**

Okay. Also auch frühzeitig eingreifen? #00:12:59#

**Interviewpartnerin:**

Genau. #00:13:00#

**Interviewer:**

Wenn er dir jetzt sehr strenges Feedback gibt, wie würde es dir damit gehen? #00:13:07#

**Interviewpartnerin:**

Also ich persönlich fühle mich von Feedback nie negativ irgendwie angesprochen oder so, wenn sie wirklich / also wenn das wirklich konstruktive Kritik ist. Also wenn wirklich was falsch war, dann finde ich Feedback sehr wichtig und nehme es auch gerne an, damit ich halt das in Zukunft besser machen kann. Und ich meine, wenn er berechtigt ist, mir strenges Feedback zu geben, fände ich es auch nicht schlimm. #00:13:31#

**Interviewer:**

Okay, ist gut. Wie gut, meinst du, kann Pepper für die Zukunft seines Teams Pläne erstellen oder Ziele setzen? #00:13:43#

**Interviewpartnerin:**

Also ich denke, er wird es so theoretisch gut machen, aber ich glaube, praktisch ist das ja immer mal was anderes. Also wenn man mal sieht, wenn man einen Businessplan für irgendwas aufstellt, dann merkt man ja, dass man überall Puffer einplanen muss, weil immer irgendwo was nicht so läuft, wie es sollte. Und ich glaube, da bräuchte er halt vielleicht auch ein bisschen Anlaufzeit, das er erst mal ein paar Fehler macht sozusagen und merkt: Okay, es könnte sein, dass irgendwas nicht reibungslos abläuft. Und dann quasi schon vorausschauend irgendwie ein bisschen was mit einplant. Also ich denke, er bräuchte da, ja, schon einen Lernprozess. #00:14:22#

**Interviewer:**

Okay. Also müsste er schon mal ein paar Projekte durcharbeiten? #00:14:28#

**Interviewpartnerin:**

Genau. #00:14:30#

**Interviewer:**

Also am Anfang würde er Sachen falsch einschätzen? #00:14:33#

**Interviewpartnerin:**

Genau. #00:14:35#

**Interviewer:**

Wie viel Zeit man braucht, solche Sachen. #00:14:36#

**Interviewpartnerin:**

Mhm (zustimmend). #00:14:37#

**Interviewer:**

Aber sonst würde es schon gehen? #00:14:39#

**Interviewpartnerin:**

Ja, oder vielleicht vergisst er auch einzelne Schritte oder so anfangs und wenn er das dann sozusagen selbst erlernt hat, dann denke ich, wird er das ganz gut machen. #00:14:49#

**Interviewer:**

Okay, also mit der Zeit wird es dann immer besser und wird dann auch passen. #00:14:53#

**Interviewpartnerin:**

Genau. #00:14:53#

**Interviewer:**

Was würdest du vermuten: Wie erfolgreich wäre ein Team, das von Pepper geführt wird? #00:14:59#

**Interviewpartnerin:**

Also das ist schwierig zu sagen, da das ja nicht nur von Pepper abhängt, würde ich sagen. Also es hängt ja vom ganzen Team ab und auch vielleicht von den Ideen, die das Team dann hat. Und ja, wie gut sie Sachen rüberbringen. Deswegen finde ich, kann man das überhaupt nicht sagen, also ob er jetzt erfolgreicher wäre als mit einem menschlichen Chef, sag ich mal. Also finde ich, kann man nicht so im voraus sagen. #00:15:29#

**Interviewer:**

Wollte ich gerade sagen: Wenn du dir jetzt vorstellst, zwei äquivalente Teams, einmal mit Pepper, einmal mit einem Menschen als Führungskraft. Was würdest du da sagen, wer erfolgreicher wäre? #00:15:41#

**Interviewpartnerin:**

Also ich würde sagen, wahrscheinlich wäre Peppers Team erfolgreicher, weil der ja rational wahrscheinlich handelt und auch objektiv ist oder objektive Sichtweisen hat. Und, ja, deswegen denke ich, dass Pepper da härter durchgreifen würde und nicht so Rücksicht auf Kollegen oder sonstige Sachen nehmen würde. Und deshalb denke ich, dass der dann am Ende auch erfolgreicher wäre. #00:16:09#

**Interviewer:**

Okay. Also dadurch, dass er rational arbeitet, wenig Rücksicht auf Verluste nimmt, dadurch wäre er dann erfolgreicher? #00:16:18#

**Interviewpartnerin:**

(unv. #00:16:20#)

**Interviewer:**

Okay. Dann sind wir auch schon durch.
