# Supplementary material for: Robot leadership–Investigating human perceptions and reactions towards social robots showing leadership behaviors
Source: PLoS One. 2023 Feb 16;18(2):e0281786. doi: 10.1371/journal.pone.0281786 (PMC9934409; doi:10.1371/journal.pone.0281786)
Supplement: S1 Dataset — (ZIP) [file pone.0281786.s002.zip › Interview_Participant8.doc]

**17 Interview**

**Interviewpartner:**

Also ich soll das jetzt noch mal erklären oder wie? #00:00:03#

**Interviewer:**

Ich stelle jetzt ein paar Fragen. #00:00:05#

**Interviewpartner:**

Ach so, okay. #00:00:06#

**Interviewer:**

So als Prämisse sollst du dir vorstellen, du arbeitest jetzt in einem Team mit Pepper als Führung, so als Prämisse. Welche Gedanken sind dir durch den Kopf gegangen, während Pepper mit dir geredet hat? #00:00:20#

**Interviewpartner:**

Die Qualität des Lautsprechers ist grottenschlecht. Überhaupt ist das / das ist echt schade, muss ich sagen, bei diesem Pepper. Der ist ja weltweit im Einsatz auch, in Hotels und so. Ich hatte schon mal eine Begegnung mit ihm. Ich hoffe, es ist nicht schlimm, oder? Das verfälscht jetzt nicht die Ergebnisse? Ich war mal bei Sylvia im Büro und da hat sie das Ding nämlich angemacht. #00:00:44#

**Interviewer:**

Ich nehme es dann nachher auf, aber / #00:00:47#

**Interviewpartner:**

Aber was ich überhaupt nicht verstehe: Jedes durchschnittliche Laptop hier, das hat einen besseren Sound oft, solche Dinger, wie hier. Die haben ja teilweise schon Stereoanlagenqualität und da sind so billige Lautsprecher, das versteht man kaum. Also ich verstehe nicht, warum man da / warum das so eine billige Lösung ist? Weil es ist ja nicht teuer, einen gescheiten Lautsprecher zu machen. Und auch das Plastik ist sehr billig. Also ich glaube, über die Zeit wird das Risse kriegen und so. Also verglichen damit, dass das Teil ja sehr teuer ist, ist es ein Witz von der Qualität. Und sonst: Man muss halt genau hinhören, weil es wahnsinnig knarzt und die Piepsstimme ist auch nicht sehr angenehm, finde ich. Also man könnte auch überlegen, eine tiefere Stimme da zu machen. #00:01:38#

**Interviewer:**

Also sehr drauf geachtet, wie / Pepper, die Qualität vom Roboter an sich und die Stimme auch sehr? #00:01:49#

**Interviewpartner:**

Ja. #00:01:49#

**Interviewer:**

Wie hat es sich für dich angefühlt, von Pepper angesprochen zu werden? #00:01:55#

**Interviewpartner:**

Das war soweit in Ordnung. Ich meine, gerade diese Piepsstimme, sagen wir mal, dadurch hat man schon den Eindruck, als würde man mit einem Kind reden oder es ist ein Kind oder so. Also das ist ein bisschen merkwürdig vielleicht, ein bisschen irritierend. Ansonsten gut, ich meine, angesichts der Tatsache, dass man sich in Zukunft an so was gewöhnen wird müssen, war es in Ordnung so weit. Ich habe mich der Aufgabe gestellt und ihn damit natürlich auch ernst genommen. #00:02:39#

**Interviewer:**

Ja, klar. Ja. Also du hast ein bisschen gedacht: Wieder die Stimme und so das Gefühl, das ist ein bisschen kindlich. Aber trotzdem halt (unv. #00:02:51#)

**Interviewpartner:**

(unv. #00:02:53#) ich habe mich auf den Inhalt konzentriert, um dann da die Aufgabe zu lösen. #00:02:57#

**Interviewer:**

Sehr gut. Welche Gedanken hast du dir gemacht, als Pepper gesagt hat: „My vision ist hat we as them marketing department can make people appassionate about our product.“? #00:03:11#

**Interviewpartner:**

Nichts Besonderes. Das ist ja das Ziel einer Marketingabteilung. #00:03:18#

**Interviewer:**

Okay, einfach nur als Statement angenommen und auch nicht überraschend gefunden. #00:03:22#

**Interviewpartner:**

Nein. #00:03:25#

**Interviewer:**

Wie gut hat Pepper seine Vision für die Zukunft definiert und präsentiert? #00:03:31#

**Interviewpartner:**

Ich finde, gut. Durchschnittlich gut. Es war jetzt nicht wahnsinnig überraschend, aber ich finde, er hat es gut gemacht. Er hat es begründet, dass es ja doch irgendwie für fast jeden geeignet sei und so. Also die Vision war da. #00:04:02#

**Interviewer:**

Auch gut präsentiert? #00:04:06#

**Interviewpartner:**

Im Rahmen seiner eingeschränkten Möglichkeiten, ja. #00:04:10#

**Interviewer:**

Pepper hat auch gesagt: „As a leader I always want to be a role model.“ Was hältst du davon? Inwiefern meinst du, dass Pepper ein Vorbild für seine Mitarbeiter sein kann? #00:04:22#

**Interviewpartner:**

Tja, schwer zu beurteilen. Man weiß ja nicht, welche Empathien er dann wirklich hat. Das ist schwer zu sagen. Hier ist ja irgendwie Aufgabenstellung und Präsentieren einer Vision, daraus lässt sich ja nicht schließen, ob er jetzt auch die empathischen Qualitäten hat einer modernen Führungskraft. #00:04:48#

**Interviewer:**

Wenn du jetzt vermuten müsstest, was würdest du da sagen? #00:04:56#

**Interviewpartner:**

Vor dem Hintergrund, dass er ja auch immer schlauer wird oder in Zukunft die Roboter auch immer schlauer werden: Wahrscheinlich würde es tatsächlich / würde so ein Roboter, wenn er dann wirklich als Führungskraft eingesetzt wird, dann wird er Möglichkeiten haben oder Möglichkeiten dazulernen, wie er tatsächlich auf die Mitarbeiter eingeht, um deren Problem zu lösen. Ja, möglich. #00:05:33#

**Interviewer:**

Also auf lange Sicht schon möglich? #00:05:40#

**Interviewpartner:**

Wir wissen, ab 2045 – du wirst es auch wissen – werden die Maschinen schlauer als die Menschen, also so geht die Prognose. Dass die Rechenleistung dann die Gehirnleistung des menschlichen Gehirns übersteigt oder gleichzieht. Gut, die Frage ist, inwieweit solche Themen wie Ironie, Subtilität des Ausdrucks etc. von einer Maschine abgedeckt werden können. Aber im Grunde, natürlich wird es kommen. Da wird es gar keinen Ausweg geben, befürchte ich. Ich sehe das nicht besonders rosig, aber es wird halt so laufen. #00:06:19#

**Interviewer:**

Wieso siehst du es eher negativ? #00:06:23#

**Interviewpartner:**

Ja gut, ich meine, meine Prognose ist: Wenn die Roboter schlauer werden als die Menschen, wirklich die intellektuelle Kapazität die einfach übersteigt, dann sind sie uns überlegen. Und dann wird es spannend. Dann müssen wir uns warm anziehen. Es gibt in der Literatur, in der Kultur so weit ja zahlreiche Distopien, die da entwickelt werden. Die halte ich auch für realistisch – leider. Also ich sehe das sehr pessimistisch alles, muss ich sagen. Ich bin Pessimist, der Recht hat; der Optimist hat das schönere Leben. Das ist so bei mir: Ich weiß das, aber meiner Meinung nach ist es klar: Wenn die schlauer werden als wir, sind wir wahrscheinlich denen nur noch lästig und werden uns relativ schnell ausklinken auf eine Art. Der Film „Matrix“ ist – ich weiß nicht, ob du den noch kennst / der ist schon ein paar Jahre alt jetzt, diese Matrix-Trilogie, ist ein realistisches Szenario, eines von mehreren. #00:07:24#

**Interviewer:**

(unv. #00:07:26#) okay. #00:07:26#

**Interviewpartner:**

Ich meine, die könnten ja auch alles selber machen. (unv. #00:07:30#) mit Energie versorgen, wäre ja kein Problem. Mit Solar oder was. Brauchen uns nicht mehr. #00:07:34#

**Interviewer:**

Okay. Das ist eine etwas pessimistische Aussicht. #00:07:40#

**Interviewpartner:**

Ja, sehr pessimistisch. Ja gut, aber bei nüchterner Betrachtung. Digitalisierung heißt auch Verlust von Autonomie des Menschen, Entmündigung und Kontrolle. Schau dir an: Du kriegst ständig Mails; wo ist da die Freiheit an deinem Arbeitsplatz, wenn ständig im Minutentakt Aufgaben kommen. Mails heißt Aufgaben. War früher nicht so. Da saßt du in deinem Büro und dass jede Minute der Chef reinkam mit irgendeinem Auftrag, war auch unwahrscheinlich. Dass auch ständig das Telefon geklingelt hätte mit irgendwas, war weniger wahrscheinlich als jetzt im Minutentakt E-Mails. #00:08:25#

**Interviewer:**

Okay, ja, stimmt schon. Ja. Wenn du jetzt in einem Team wärst mit Pepper als Führungskraft: Würdet ihr gemeinsame Ziele anstreben, die eventuell auch von Pepper gesetzt werden? #00:08:44#

**Interviewpartner:**

Wenn wir als Team Pepper als Führungskraft akzeptieren, dann würden wir das machen. Ich meine, ich könnte mir natürlich / persönlich könnte ich mir nicht vorstellen, jetzt wirklich einen Roboter als Chef zu haben. Aber zum Beispiel jetzt in speziellen Projekten, wo einfach der Roboter da mehr weiß oder es gibt ja jetzt schon Kontexte, wo man sich auf den Roboter verlässt, dann ist es natürlich sinnvoll, wenn sich das Team an die Weisungen oder Empfehlungen des Roboters hält. #00:09:27#

**Interviewer:**

Du persönlich würdest es aber eher nicht so akzeptieren? #00:09:34#

**Interviewpartner:**

Als dauerhaften Chef einen Roboter: nein. Das hoffe ich, da bin ich dann schon in Rente, wenn es soweit ist. Aber für teambezogene oder aufgabenbezogene Sachen, also wäre ich jetzt Bombenentschärfer, wäre es gut, einen Roboter da vorzuschicken, der das prüft und dann natürlich auch sichere Ergebnisse liefern kann. #00:09:58#

**Interviewer:**

Ja. Aber ich meine, in so einer Situation ist dann der Roboter dem Menschen untergestellt? #00:10:04#

**Interviewpartner:**

In dem Fall ist der Mensch ja dem Roboter untergestellt. #00:10:08#

**Interviewer:**

Jetzt hier, ja. #00:10:08#

**Interviewpartner:**

Ja. Und auch beim Bomben-Entschärfen. Da ist ja maßgeblich erst mal, was der Roboter sieht, sagt, empfiehlt, was zu machen ist mit der Bombe. #00:10:16#

**Interviewer:**

Ja, aber dahinter ist halt zumindest jetzt noch in der Regel ein Mensch, der ihn steuert. #00:10:24#

**Interviewpartner:**

Steuert, genau, genau. #00:10:26#

**Interviewer:**

Aber ich meine, jetzt in diesem Szenario wäre ja der Pepper jetzt als Chef. Und da würdest du sagen, projektbezogen könnte es Sinn machen oder auch gut sein. #00:10:44#

**Interviewpartner:**

Ja. #00:10:44#

**Interviewer:**

Aber so dauerhaft / #00:10:46#

**Interviewpartner:**

Nicht als Chef, der jetzt personalrechtlich oder was weiß ich / dem man personalrechtlich unterstellt ist. #00:10:57#

**Interviewer:**

Wieso? #00:11:00#

**Interviewpartner:**

Ja gut, das wäre halt / fände ich, ist zu krass. Also da hätte ich ethisch-moralische Bedenken jetzt auch für mich selber. Das würde nicht / Das wäre kein wertschätzender Umgang, wenn ein Arbeitgeber / Also heute ist es so, das kann sich alles ändern. Aber heute, wenn ein Arbeitgeber sagt: „So, du kriegst jetzt einen Roboter als Chef“ ist nach meiner Meinung, nach jetzigen Maßstäben ethisch und moralisch und so weiter nicht kompatibel. Wie gesagt, es wäre kompatibel für einzelne Projekte, für einzelne Aufgaben, einzelne Projekte. Dann ja. #00:11:43#

**Interviewer:**

Und allgemein: Woran liegt es da? Weil dann Roboter eigentlich Entscheidungen über einen Menschen / #00:11:52#

**Interviewpartner:**

Genau. Und dann sind wir wieder beim Thema Autonomie. Denn Autonomie sollte der Mensch behalten. Trotz allem. Das wäre ja die komplette Konterkarierung. #00:12:04#

**Interviewer:**

Ja, schon. Okay. Inwiefern hast du das Gefühl, dass Pepper hohe Erwartungen an euch als Team hätte? #00:12:14#

**Interviewpartner:**

Die sind durchschnittlich, würde ich sagen, die Erwartungen. Die sind schon, denke ich, erfüllbar. Andererseits, gut: Mein zweiter Gedanke ist natürlich: Dadurch, dass er natürlich auch viel weiß, er hat ja Verbindung zum Internet, er hat einen Algorithmus, der dazu lernt, etc., etc., also das kann sich auch ändern. Er kann auch davon ausgehen, dass man unbegrenzte Ressourcen hat, so wie er und dann, wenn das Maßstab ist, das kann es natürlich dann nicht sein. Also dann wären die Anforderungen zu hoch. Wir müssen schlafen, wir müssen essen und so weiter, wir können nicht immer / Also klar, wir können auch mal schnell ins Internet gehen, aber er würde es wahrscheinlich tatsächlich schneller finden als wir mit Google eintippen und so weiter. Also wir wären langsamer und wir bräuchten Pausen im Gegensatz zu ihm. #00:13:12#

**Interviewer:**

Und du meinst, er würde das nicht berücksichtigen? #00:13:16#

**Interviewpartner:**

Kommt drauf an. Das kann man / Er würde es erst mal wahrscheinlich nicht berücksichtigen. Er würde dann dazulernen: Hoppla, die Menschen brauchen auch Pausen. #00:13:29#

**Interviewer:**

Dann würde er mit der Zeit halt lernen, dass / #00:13:32#

**Interviewpartner:**

Wahrscheinlich. Das würde ich jetzt mal einfach naiv annehmen, weil ich meine, es müssen ja Ergebnisse rauskommen. Und die müssen in vernünftigem Rahmenbedingungen auch gesetzt werden, die Ziele. #00:13:50#

**Interviewer:**

Weil sonst jeder / #00:13:51#

**Interviewpartner:**

Und da gehört ja das dazu. Genau. #00:13:53#

**Interviewer:**

In welchem Ausmaß, glaubst du, dass Pepper dich als Teammitglied angemessen in deiner Arbeit unterstützen kann? #00:14:02#

**Interviewpartner:**

Das wird er sicher können. Er könnte schnell Informationen liefern. Das mit Sicherheit. #00:14:17#

**Interviewer:**

Okay, Informationen liefern. Meinst du, er würde da auch an irgendwelche Grenzen stoßen irgendwann? #00:14:24#

**Interviewpartner:**

Möglicherweise ja. #00:14:25#

**Interviewer:**

Bei welchen Sachen? #00:14:28#

**Interviewpartner:**

Ich meine, gerade zum Beispiel bei diesem Gartenthema. Da gibt es ja auch viel Erfahrungswissen. Vorher habe ich schon die Oma genannt, die das halt alles weiß, die hat einen grünen Daumen, die weiß, wie man mit Pflanzen umgeht. Das Wissen wird ja auch weitergegeben an die Kinder, an die Enkel, etc. Und das ist ein Erfahrungswissen, das ersetzt der Roboter nicht so leicht, glaube ich, so mit einem Schwung. Ich glaube, Pepper könnte sich nicht sofort in diesen Sensor reinversetzen zum Beispiel. Wobei: Wer weiß? Wenn die verbunden sind miteinander übers Netz? Dann ja. Schwer zu sagen. Aber es gibt Erfahrungswissen, was jetzt nicht 1 : 1 im Internet abbildbar ist oder nicht 1 : 1 im Internet zu finden ist. Dazu gehört, glaube ich, das. Natürlich gibt es auch unendliche Homepages und Blogs über Garten und Gärtnern, zu jeder Pflanze wird es da was geben. Aber trotzdem ist es etwas anderes, als wenn man es wirklich selbst gemacht hat. Und das hat ja der Pepper nicht in dem Fall; der hat nicht gegartelt bis jetzt. #00:15:39#

**Interviewer:**

Also das sind dann so Informationen, woran er nicht wirklich kommt, so Erfahrungswerte. #00:15:47#

**Interviewpartner:**

Er kann ja einen Text vorlesen zu dieser Pflanze, die da ist, keine Ahnung, was das ist. Da würde er mir einen Text vorlesen mit der besten Pflege, was man machen muss. Aber trotzdem, das ist nicht zu schlagen durch Erfahrungswissen. #00:16:02#

**Interviewer:**

Das sind dann immer noch Informationen, die nicht wirklich dokumentiert sind und dann er auch nicht hat? #00:16:05#

**Interviewpartner:**

Genau, ja. #00:16:07#

**Interviewer:**

Inwiefern hast du das Gefühl, dass sich Pepper an dich und Andere anpasst? #00:16:15#

**Interviewpartner:**

Das würde er sicher tun. Ja, also das würde er tun, einfach, weil der Algorithmus dazulernt. Also ich denke schon, er würde auch im Umgang mit den einzelnen Teammitgliedern und mir dazulernen, natürlich. #00:16:36#

**Interviewer:**

Und dann auch so persönliche Besonderheiten oder Bedürfnisse dann auch lernen und sich da anpassen? #00:16:45#

**Interviewpartner:**

Ja. #00:16:46#

**Interviewer:**

Inwiefern hat dich Pepper angeregt, neue und innovative Ideen zu produzieren? #00:16:57#

**Interviewpartner:**

Ich würde sagen, also vielleicht auf einer Skala 1 von 100: 25, also ein Viertel so. So wahnsinnig angeregt hat er mich nicht, da war es auch zu kurz. #00:17:20#

**Interviewer:**

Und selbe Richtung. Denkst du, dass Pepper dich ermutigen würde, den Status quo anzuzweifeln? #00:17:30#

**Interviewpartner:**

Wie jetzt Status quo? Dass er die Führungskraft ist und ich sein Mitarbeiter? Das ist der Status quo. Den würde ich anzweifeln. #00:17:40#

**Interviewer:**

Nein, also eher so gedacht. Arbeitsweisen, Prozesse in der Arbeit, solche Sachen. Würde er dich da anregen, das zu hinterfragen, neue Ansätze zu suchen? #00:17:56#

**Interviewpartner:**

Ich denke nicht, weil er hat ja auch erst mal das Schema F adaptiert wahrscheinlich. Und würde erst mal sich daran orientieren. Ich glaube nicht, dass er gleich zum Hinterfragen von irgendwelche Regeln, Strukturen, sonst was aufrufen würde. Nein. #00:18:17#

**Interviewer:**

Erst einmal. Aber / #00:18:19#

**Interviewpartner:**

Glaube ich nicht, nein. Er folgt ja der Logik der Algorithmen. #00:18:25#

**Interviewer:**

Und dadurch, dass du es so formuliert hast: erst einmal. Meinst du, dann mit der Zeit, dass er damit anfangen würde, Sachen zu hinterfragen und / #00:18:37#

**Interviewpartner:**

Dass er wirklich grundsätzliche Sachen hinterfragt, kann ich mir nicht vorstellen. Dass er Prozesse hinterfragt: ja, okay. Vor allem dann, wenn der Output nicht so ist, wie er es sich vielleicht vorstellt oder wie er es errechnet hat. Von Vorstellung wollen wir vielleicht nicht sprechen. #00:19:01#

**Interviewer:**

Dann vielleicht schon? #00:19:03#

**Interviewpartner:**

Ja, in eingeschränktem Maße zu einem späteren Zeitpunkt, ja. #00:19:08#

**Interviewer:**

Würdest du für Pepper oder halt für die Ziele, die von Pepper gesetzt werden, mehr als das zwingend Notwendige tun? #00:19:20#

**Interviewpartner:**

Gute Frage. Kommt auf die Aufgabe an. Kommt auf die sinnstiftende Art oder, ja, die Sinnhaftigkeit der Aufgabe an. Wenn ich keinen Sinn sehe in der Aufgabe, würde ich garantiert nicht mehr machen, als es unbedingt von Pepper verlangt wird. #00:19:51#

**Interviewer:**

Aber wenn du die / #00:19:52#

**Interviewpartner:**

Wahrscheinlich weniger auch, kommt drauf an. Je nachdem, welche Konsequenzen da bestehen. Wenn die Aufgabe sinnhaft ist für mich, würde ich mehr machen. Das mache ich ja jetzt auch in meinem Kontext ohne Roboter. #00:20:10#

**Interviewer:**

Also würdest du es nicht so an der Führungskraft festmachen, sondern an der Aufgabe an sich, ob die sinnvoll ist oder nicht? #00:20:16#

**Interviewpartner:**

Ja. #00:20:18#

**Interviewer:**

Wie war dein Gesamteindruck von Pepper? #00:20:23#

**Interviewpartner:**

Es war halt ein Roboter, der jetzt noch nicht so viel kann und so viel weiß. Aber es ist klar, dass sich das ändern wird. #00:20:48#

**Interviewer:**

Okay. Also erst mal die Fähigkeiten als nicht so weit empfunden, aber trotzdem so ein bisschen Zukunftsblick? #00:20:57#

**Interviewpartner:**

Ja, ja, genau. #00:21:00#

**Interviewer:**

Inwiefern hast du dich von Pepper motiviert gefühlt? #00:21:04#

**Interviewpartner:**

Ja gut, durchschnittlich. Ich meine, ich habe mich jetzt bereit erklärt, bei diesem Test mitzumachen. Dann macht man es halt. Und es ist natürlich auch ein dankbares Thema, mit dem man was anfangen kann: Garten und so, Gärtnern. #00:21:26#

**Interviewer:**

Und wenn du dir jetzt vorstellst, du würdest für ihn arbeiten: Würdest du dich von ihm motiviert fühlen oder motivieren lassen? #00:21:34#

**Interviewpartner:**

Ja, das schon. Ich meine, das hängt ja wie gesagt, ab von der Sinnhaftigkeit der Aufgabe. Aber ja. #00:21:43#

**Interviewer:**

Wieder von der Aufgabe abhängig? #00:21:43#

**Interviewpartner:**

Ja. #00:21:43#

**Interviewer:**

Fühlst du dich von Pepper wertgeschätzt? #00:21:47#

**Interviewpartner:**

Nein, eigentlich nicht. Er hat ja kein Bewusstsein in dem Sinn. Es ist eine Maschine. Also das kann man schlecht sagen. Wir haben ja jetzt nicht interagiert, er hat ja nur seinen Text da runtergespielt, den ihm auch jemand gesagt hat. Also da ist ja alles noch in Anfängen, das ist noch ganz eindeutig nur die Maschine. Noch, wie gesagt, noch. Aber jetzt im Moment: Nein, kann man nicht davon ausgehen, dass da echte Wertschätzung entgegenkommt. Das wird immer künstlich sein, auch wenn der Roboter noch so schlau ist. #00:22:43#

**Interviewer:**

Wollte ich gerade sagen. Wenn du dir jetzt vorstellst, du arbeitest für ihn und machst deine Arbeit, reichst sie ein und dann sagt er. Hey, gut gemacht. #00:22:52#

**Interviewpartner:**

(unv. #00:22:52#), weil er halt so programmiert ist oder dass er sich das beigebracht hat. Das ist ja im Endeffekt das Gleiche: Es ist nur eine Maschine. #00:23:02#

**Interviewer:**

Okay. Also dann auch nicht wirklich ernst / würdest du nicht wirklich ernst nehmen und auch nicht wertgeschätzt fühlen? #00:23:09#

**Interviewpartner:**

Nein. Ich weiß halt: Okay, es ist Bestandteil seines Aufgabenspektrums als Führungskraft, dass er sein Team auch mal lobt. Gut. Ich kann da keine emotionale Bindung aufbauen und würde dann dort (unv. #00:23:34#) wertgeschätzt fühlen. #00:23:34#

**Interviewer:**

Einfach so gesehen: Er ist halt so programmiert und ist halt so. #00:23:40#

**Interviewpartner:**

Er ist halt so, genau. Er macht (unv. #00:23:42#)

**Interviewer:**

Denkst du, Pepper ist fair zu seinen Teammitgliedern? #00:23:46#

**Interviewpartner:**

Ja, das denke ich schon. #00:23:51#

**Interviewer:**

Wieso? #00:23:51#

**Interviewpartner:**

Na ja, ich meine, auch da wird er ja nach Logik vorgehen; mathematisch oder Logik der Algorithmen. Und da wird man ihm schon empfohlen haben, dass er gerecht ist. #00:24:19#

**Interviewer:**

Also dadurch, dass er so nüchtern und objektiv ist, wird er dann auch so handeln oder hal so werten? #00:24:29#

**Interviewpartner:**

Ja, genau. #00:24:31#

**Interviewer:**

Kannst du Pepper als Führungskraft vertrauen? #00:24:34#

**Interviewpartner:**

Nein. #00:24:35#

**Interviewer:**

Wieso? #00:24:35#

**Interviewpartner:**

Wie gesagt, es ist auch nur eine Maschine. Was man ihm anvertraut, wandert halt in seinen Algorithmus, vielleicht ins Internet oder was immer. Also man weiß ja nicht, wo das hinwandert alles, was man ihm sagt. #00:24:54#

**Interviewer:**

Also würdest du ihm nichts anvertrauen? #00:24:57#

**Interviewpartner:**

Nein. Ich bin auch nicht bei Facebook und solche Scherze. #00:25:01#

**Interviewer:**

Und wenn du ihn jetzt als Chef hättest, würdest du vertrauen, dass er die richtige Entscheidung trifft, Aufgaben / #00:25:10#

**Interviewpartner:**

Wenn es nach Logik geht, ja natürlich. Wenn es nach sachlicher Logik geht, würde er sicherlich die richtigen Entscheidungen treffen. #00:25:22#

**Interviewer:**

Also auf sachlicher Ebene / #00:25:24#

**Interviewpartner:**

Genau. Bei zwischenmenschlichen Dingen wohl kaum. Also würde er nicht immer treffsicher da die richtigen Entscheidungen (treffen). Aber da können normale Führungskräfte auch nicht unbedingt. #00:25:42#

**Interviewer:**

Welche Verhaltensweisen waren dir sympathisch? #00:25:48#

**Interviewpartner:**

Na, immerhin: Er hat einen angeschaut. Er hat die Arme geöffnet und so. Das war nett programmiert. #00:26:07#

**Interviewer:**

Also so ein bisschen die Gestik? #00:26:09#

**Interviewpartner:**

Ja, ja, gut, ja. Aber wie gesagt, immer im Bewusstsein, es ist halt so einprogrammiert von der Maschine. #00:26:18#

**Interviewer:**

Also immer mit dem Hintergedanken, es wurde so gesagt. Was hat dich an seinem Verhalten gestört? #00:26:29#

**Interviewpartner:**

Ja, wie gesagt, schon der schlechte Sound und diese knarzende Etwas. Das ist wirklich erstaunlich schlecht, also keine Ahnung, ob das dem Hersteller bewusst ist oder wie auch immer. Es ist wirklich ein Rätsel, warum der Sound so grottenschlecht ist. #00:26:54#

**Interviewer:**

Wie würde es dir gehen, wenn Pepper dein Chef wäre? #00:27:00#

**Interviewpartner:**

Nicht gut. Das würde ich nicht mitmachen. Also wie schon gesagt: Für Einzelprojekte okay, aber nicht als Chef, als dauerhafter Chef. #00:27:11#

**Interviewer:**

Würdest du ablehnen? #00:27:13#

**Interviewpartner:**

Würde ich nicht machen. #00:27:15#

**Interviewer:**

Du würdest eventuell dann das Unternehmen wechseln oder / #00:27:19#

**Interviewpartner:**

Wo es keinen Roboter als Chef gibt, ja. Klar, man kann so ein Projekt wie Marketing, auch das wäre ein Einzelprojekt, das Marketing für diesen Pflanzenfühler da / Okay, das wäre alles noch in Ordnung, aber nicht als Chef. #00:27:40#

**Interviewer:**

So Projektleiter für einzelne Projekte? #00:27:41#

**Interviewpartner:**

Ja, das von mir aus. #00:27:45#

**Interviewer:**

Aber so dauerhaft nicht. Wenn er sehr strenges Feedback gibt, wie würdest du damit umgehen? #00:27:51#

**Interviewpartner:**

Wenn er strenges Feedback gibt? (überlegt länger) Ich würde es weniger ernst nehmen, als wenn es wirklich von einem Menschen käme, das schon. #00:28:14#

**Interviewer:**

Okay. Wieso? #00:28:14#

**Interviewpartner:**

Ja, wie gesagt, es ist eine Maschine und ich kann ja dann auch wieder / vielleicht muss ich davon ausgehen, dass er tatsächlich dann eben nicht weiß, wie leistungsfähig der Mensch ist. Beziehungsweise muss man ja besser sagen: Wie eingeschränkt der Mensch ist in seiner Leistungsfähigkeit, was jetzt Ausdauer und solche Themen betrifft, wie ein Roboter. Also mit diesem Bewusstsein kann man natürlich das Feedback nicht so ernst nehmen, also wenn man davon ausgeht, dass er halt denkt, man arbeitet wie er. #00:29:03#

**Interviewer:**

Also würdest du es nicht so ernst nehmen und auch nicht / #00:29:05#

**Interviewpartner:**

Ich würde es nicht so ernst nehmen. #00:29:07#

**Interviewer:**

Und auch nicht wirklich dran arbeiten, was er gesagt hat? #00:29:09#

**Interviewpartner:**

Ich würde mir überlegen, wo er vielleicht Recht hat. Das schon. #00:29:14#

**Interviewer:**

Also meinst du, er würde auch teilweise unberechtigtes Feedback geben? #00:29:21#

**Interviewpartner:**

Ja, klar. #00:29:24#

**Interviewer:**

Wie gut, meinst du, kann Pepper für die Zukunft seines Teams Pläne erstellen und Ziele setzen? #00:29:33#

**Interviewpartner:**

Ja, auch hier wieder: Nach sachlicher Logik würde er es sicher gut hinkriegen. Die Frage ist halt, ob er die Kapazität des Menschen im Auge hat, die Leistungsfähigkeit. Aber angenommen, er würde das alles berücksichtigen, was ja auch nur eine Frage des Lernens ist, des Programmierens oder wie auch immer, des Datenaustausches letztlich, dann würde er wahrscheinlich tatsächlich realistische Leistungspläne erstellen können. Aber wie gesagt, unter dem Vorbehalt, dass er es halt weiß, dass er die Parameter kennt. Und da habe ich halt, wie schon gesagt, Zweifel am Anfang. Das gilt auch für das Feedback. Auch für die Aufgabenstellung generell ist halt dann immer die Frage: Kann er das Leistungsvermögen des Menschen richtig einschätzen? #00:30:42#

**Interviewer:**

Und da sagst du, am Anfang wird er falsch einschätzen, aber mit der Zeit würde er es dann lernen? #00:30:48#

**Interviewpartner:**

Wahrscheinlich. #00:30:51#

**Interviewer:**

Letzte Frage: Was würdest du vermuten, wie erfolgreich wäre ein Team, das von Pepper geführt wird? #00:30:57#

**Interviewpartner:**

Hängt ab von den Teammitgliedern. Wenn die das akzeptieren. Hängt ab echt von den Teammitgliedern. #00:31:08#

**Interviewer:**

Also wenn die Teammitglieder ihn als Chef akzeptieren? #00:31:14#

**Interviewpartner:**

Kann sein. Man hat ja oft so die Erfahrung mit jüngeren Leuten, ich rede jetzt von mir, die halt frisch von der Uni sind: Die sind total ehrgeizig. Die machen dann hier ihre Doktorarbeit oder irgendwas oder arbeiten auch bei unserem Team. Sie haben totalen Ehrgeiz und eine hohe Leistungsfähigkeit im Gegensatz zu mir. Ich bin 40, ich merke das natürlich. Und jetzt so ein Team von lauter 23-Jährigen frisch mit Abschluss oder die halt wirklich jetzt ihre erste berufliche Arbeit haben, die würden natürlich wahrscheinlich / die auch näher an der Technik sind als digital natives im Gegensatz zu den (unv. #00:32:02#), die würden da wahrscheinlich tatsächlich / die hätten da Lust drauf vielleicht, also nehmen wir es mal an. Und würden da sicher sehr gute Ergebnisse liefern. Die wären wahrscheinlich auch bereit dann, wenn Pepper da zu hohe Ziele setzt, das irgendwie aufzuholen; nachts zu arbeiten oder so was. Wenn die richtig motiviert sind, wenn man denen – keine Ahnung – Belohnungen am Ende in Aussicht stellt oder wie auch immer, dann würden sich da Teams finden, die extrem gute Leistungen machen würden. Aber ich glaube nicht, wenn es eine Vergleichsgruppe gäbe oder es wäre ein Chef da, der mit ganz viel Charisma da die Leute auch pushen würde bis aus Letzte, dann wäre die Kontrollgruppe von mir aus genauso gut, vielleicht noch besser. Vielleicht noch besser. Also das kann man wirklich so nicht sagen. Das hängt ab einerseits von der Führungskraft, egal ob Roboter oder nicht, und von dem Team. Ein Topf von 60-jährigen Managern, die hätten da keinen Bock drauf auf so was. #00:33:17#

**Interviewer:**

Okay, ja. Das ist interessant. Also Jüngere, kannst du dir vorstellen, dass es funktionieren könnte. #00:33:25#

**Interviewpartner:**

Ja, die Affinität haben zu Robotern. #00:33:30#

**Interviewer:**

Dann würde es gehen. Okay. #00:33:33#

**Interviewpartner:**

Es ist echt stark abhängig von den Beteiligten, würde ich sagen. #00:33:37#

**Interviewer:**

Okay. Sehr gut, vielen Dank.
